# 6-Thioguanosine Monophosphate Prodrugs Display Enhanced Performance against Thiopurine-Resistant Leukemia and Breast Cancer Cells

**DOI:** 10.1021/acs.jmedchem.2c01010

**Published:** 2022-11-14

**Authors:** Sarah Moreno, Magdalena Fickl, Ingo Bauer, Melanie Brunner, Anna Rázková, Dietmar Rieder, Isabel Delazer, Ronald Micura, Alexandra Lusser

**Affiliations:** † Institute of Organic Chemistry, Center for Molecular Biosciences Innsbruck, University of Innsbruck, Innrain 80-82, 6020 Innsbruck, Austria; ‡ Institute of Molecular Biology, Biocenter, Medical University of Innsbruck, Innrain 80-82, 6020 Innsbruck, Austria; § Institute of Bioinformatics, Biocenter, Medical University of Innsbruck, Innrain 80-82, 6020 Innsbruck, Austria

## Abstract

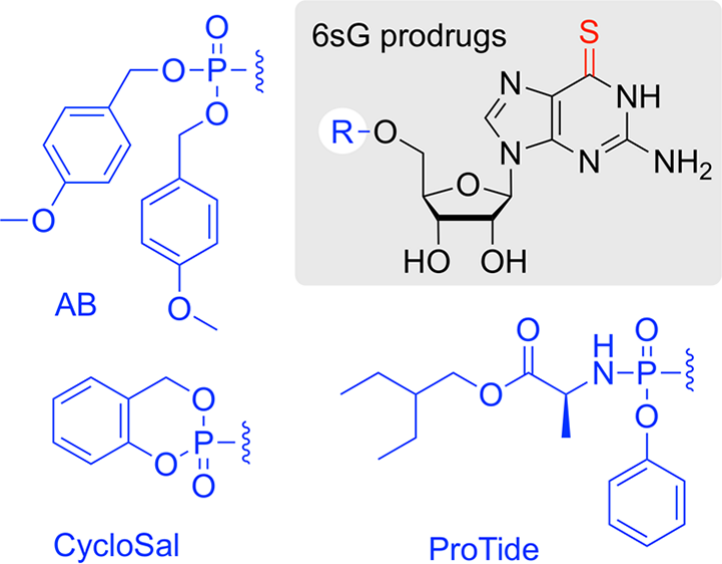

Thiopurines are in widespread clinical use for the treatment of immunological disorders and certain cancers. However, treatment failure due to resistance or adverse drug reactions are common, asking for new therapeutic strategies. We investigated the potential of 6-thioguanosine monophosphate (6sGMP) prodrugs to overcome resistance to 6-thioguanine. We successfully developed synthetic routes toward diverse 6sGMP prodrugs, tested their proliferation inhibitory potential in different cell lines, and examined their mode of action. Our results show that 4-acetyloxybenzyl- and *cyclo*Saligenyl-derivatized 6sGMP prodrugs are effective antiproliferative compounds in cells that are resistant to thiopurines. We find that resistance is related to the expression of salvage pathway enzyme HGPRT. Using TUC-seq DUAL, we demonstrate the intracellular conversion of 6sGMP prodrugs into bioactive 6sGTPs. Thus, our study offers a promising strategy for thiopurine therapy by using 6sGMP prodrugs, and it suggests TUC-seq DUAL as a simple and fast method to measure the success of thiopurine therapy.

## Introduction

**Figure 1:**
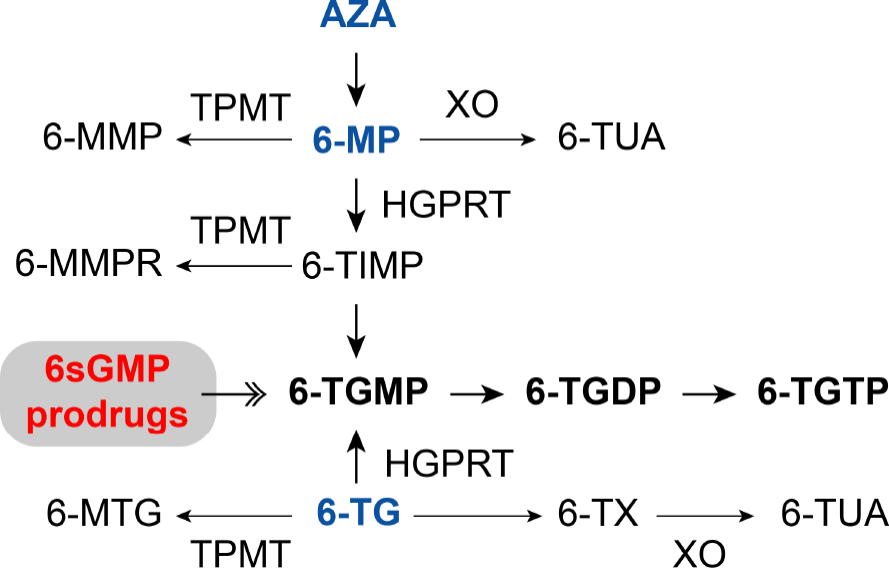
Simplified scheme of thiopurine metabolism and hypothesis for 6sGMP prodrug entry.^35^ Azathioprine (AZA), 6-mercaptopurine (6-MP), xanthine oxidase (XO), 6-thiouric acid (6-TUA), thiopurine *S*-methyltransferase (TPMT), 6-methylmercaptopurine (6-MMP), hypoxanthine-guanine phosphoribosyl transferase (HGPRT), 6-thioinosine 5′-monophosphate (6-TIMP), 6-methylmercaptopurine ribonucleotides (6-MMPR), 6-thioguanosine 5′-monophosphate (6-TGMP), 6-methylthioguanine (6-MTG), 6-thioguanosine diphosphate (6-TGDP), 6-thioguanosine triphosphate (6-TGTP), 6-thioxanthine (6-TX).

Since their development in the 1950s, thiopurines have a history as anticancer and immunosuppressant drugs.^1,2^ 6-Mercaptopurine (6-MP), its derivative azathioprine (AZA), and 6-thioguanine (6-TG) have remained in broad use for the treatment of acute lymphoblastic leukemia in children and for inflammatory diseases, such as inflammatory bowel disease (IBD).^3,4^ Although thiopurine therapy is often linked to toxicity and poor response, clinical guidelines are supportive of its use for maintenance therapy.^5,6^ Thiopurines are precursors of the actual bioactive compounds (“prodrugs”) and must be converted by enzymes of the purine salvage pathway to the metabolically active thioguanine nucleotides (TGN). Whereas several enzymatic steps are involved in the case of 6-MP and AZA, 6-TG activation requires only the enzyme hypoxanthine-guanine phosphoribosyl transferase (HGPRT) that catalyzes its conversion to 6-thioguanosine monophosphate (6-TGMP) (Figure 1). The biologically active compounds are deoxythioguanosine triphosphate (TdGTP) and thioguanosine triphosphate (TGTP). TdGTP is incorporated into DNA, which ultimately causes DNA strand breaks due to faulty repair and perturbs RNA transcription.^7^ The inhibitory association of the TGTP ribonucleoside with the Rac1 GTPase is mostly responsible for the cytotoxic effect in T-cells.^7^ The latter mode of action is considered the major mechanism by which thiopurines are effective in the treatment of IBD.^4^ However, methylation of all thiopurine bases, nucleosides, and nucleotides by the enzyme thiopurine methyltransferase (TPMT), which is associated with hepatotoxicity and myelotoxicity, or the conversion into 6-thiouric acid by xanthine oxidase (XO) results in reduced efficacy of these drugs (Figure 1). Of note, combination treatment with the XO inhibitor allopurinol favored the generation of TGNs over methylmercaptopurine^8^ and increased the activity of HGPRT.^9^ Nevertheless, a considerable number of IBD patients experience thiopurine therapy failure due to adverse effects or resistance.^10^ While 6-TG appears to be useful as rescue therapy for patients with failed 6-MP and AZA treatment, long-term efficacy was eventually reduced.^11^

**Figure 2:**
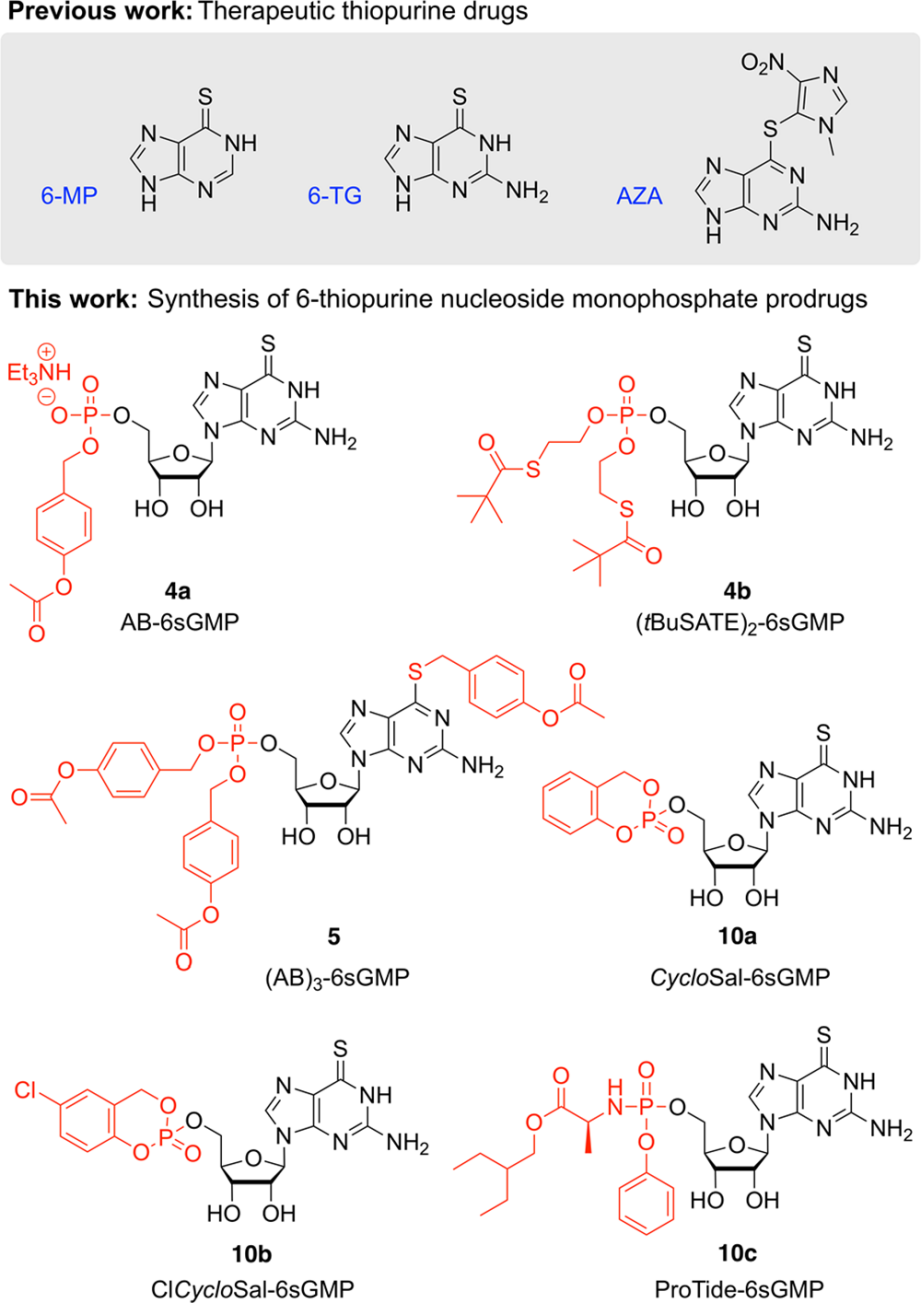
Background and overview of the work described. Chemical structures of therapeutic thiopurine drugs and the 5′-monophosphate 6sGMP prodrugs introduced in this study. 6-Mercaptopurine 6-MP, 6-thioguanine 6-TG, azathioprine AZA.

Therefore, we hypothesized that direct delivery of 6-TGMP instead of 6-TG into the cell might overcome issues with skewed thiopurine metabolism toward methylated products or resistance due to decreased HGPRT levels. Because nucleotides have a hydrophilic nature and therefore cannot cross the cellular lipid membrane, diverse approaches have been taken in the past to mask the 5′-*O*-monophoshate group with biolabile protection groups generating prodrugs that can enter the cells.^12,13^ These protection groups can be released by different cellular pathways to yield the free 5′-monophosphate nucleotide.^14^ In this work, we established the synthesis of 6-thioguanosine (6sG) monophosphate prodrugs with different protection patterns (Figure 2). We found superior efficacy of several 6sGMP prodrugs on 6-TG and/or 6sG-resistant populations of K-526 leukemia and SK-BR-3 breast cancer cells, we show the successful delivery and incorporation of the 6sGMP prodrugs into RNA, and we provide evidence for a thiopurine resistance mechanism involving *HGPRT* expression.

## Results and Discussion

### Nucleoside 5′-*O*-Monophosphate Prodrugs

Nucleoside analogs of therapeutic use are often applied as prodrugs, which represent precursors of the actual bioactive compound.^12^ They have favorable properties with respect to cellular uptake and pharmacokinetics. One concept specific for nucleoside prodrugs relies on 5′-*O*-monosphosphate derivatization.^13^ The idea is to take a shortcut in the route to the biologically active nucleoside triphosphate by providing the nucleoside monophosphate. However, the use of free nucleoside 5′-*O*-monophosphates is inefficient because of their high polarity thwarting penetration of cellular membranes. Therefore, the negatively charged oxygen atoms of the 5′-*O*-monophosphate group are masked with ‘biolabile’ groups that are released after cellular uptake.

For our undertaking toward 6sG 5′-*O*-monosphosphate prodrugs, we concentrated on established prodrug patterns, including 4-acetyloxybenzyl (AB),^15,16^*S*-pivaloyl-2-thioethyl (*t*Bu-SATE),^17,18^ and *cyclo*Saligenyl (*Cyclo*Sal) phosphates^19^ as well as phosphoramidates consisting of an amino acid ester promoiety linked via a P–N bond to the nucleoside aryl phosphate (ProTide).^20,21^ Depending on the prodrug pattern, different cellular pathways to release the free 5′-monophosphate nucleotide have been described.^14^ Surprisingly, none of these established phosphate prodrug patterns has been synthesized in context with 6-thioguanosine so far and hardly anything is known about the therapeutic potential of 6sG 5′-*O*-monophosphate prodrugs.^22,23^

### Synthesis of 6sG 5′-*O*-Monophosphate Prodrugs

**Scheme 1:**
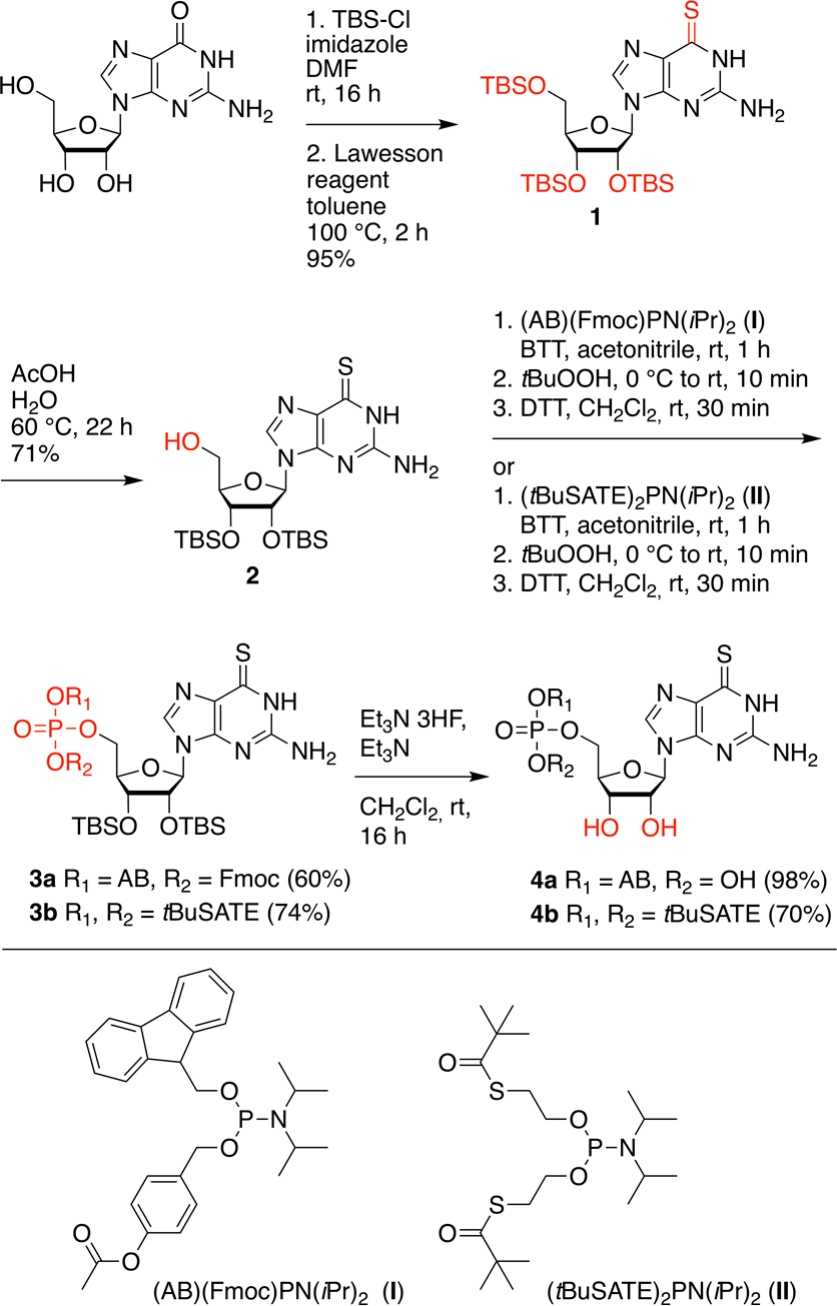
Synthesis of AB and *t*BuSATE 6sGMP Prodrugs **4a** and **4b** Abbreviations: *tert*-butyldimethylsilyl chloride (TBS-Cl), 4-acetyloxybenzyl (AB), 9-fluorenylmethoxycarbonyl chloride (Fmoc), 5-benzylthio-1*H*-tetrazole (BTT), 1,4-dithiothreitol (DTT), S-pivaloyl-2-thioethyl (*t*BuSATE).

The synthesis of 6sG prodrugs with 4-acetyloxybenzyl (AB)- and *S*-pivaloyl-2-thioethyl (*t*BuSATE) 5′-*O*-monophosphates, **4a** and **4b**, started from guanosine with protection of the three hydroxyl groups as *tert*-butyldimethylsilyl (TBS) ethers followed by treatment with the Lawesson reagent for the transformation of the 6-oxo into the 6-thio group, providing compound **1** in high yields (Scheme 1). Then, the 5′-*O*-TBS protecting group was cleaved under acidic conditions and gave precursor **2**, ready for attachment of the phosphotriester moiety. This functionalization was achieved using mild phosphor(*III*)amidite chemistry catalyzed by 5-(benzylthio)-1*H*-tetrazole. The required reagents 4-[({[bis(propan-2-yl)amino](9*H*-fluoren-9-ylmethoxy)phosphanyl}oxy)methyl]phenyl acetate **I**(24,25) and bis-(*S*-pivaloyl-2-thioethyl)-*N*,*N*-diisopropylaminophosphoramidite **II**,^26,27^ respectively, were synthesized as described earlier.^28^ Selective oxidation of the phosphite P(III) triester intermediates to the phosphate triester P(V) compounds **3a** and **3b** was accomplished using *tert*-butyl hydroperoxide at low temperature and short reaction times, immediately followed by 1,4-dithiothreitol (DTT) treatment to reduce S–S dimers and eliminate the slight excess of oxidizing reagent. Finally, the 2′-*O* and 3′-*O* TBS groups were cleaved by treatment with triethylamine trihydrofluoride to yield prodrugs **4a** and **4b**.

**Scheme 2:**
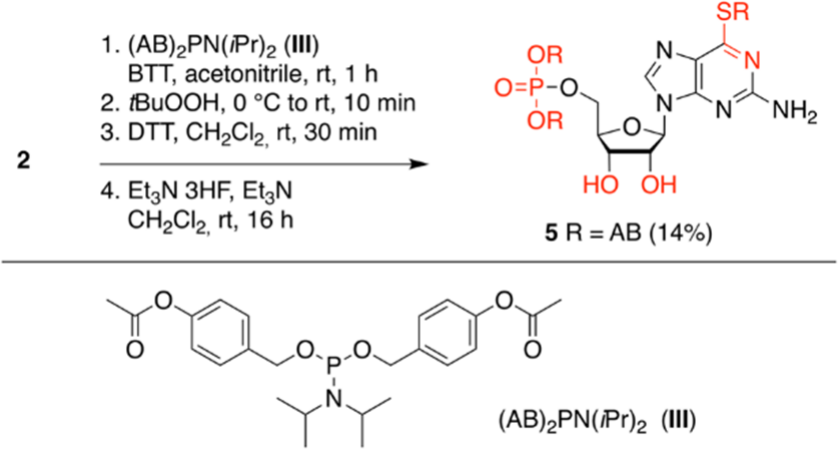
Synthesis of (AB)_3_ 6sGMP Prodrug **5** Abbreviations: 4-acetyloxybenzyl (AB), 5-benzylthio-1*H*-tetrazole (BTT), 1,4-dithiothreitol (DTT).

Prodrug **5** was obtained by a fast reaction sequence starting from precursor **2**, applying the reagent bis-(4-acetyloxybenzyl)-*N*,*N*-diisopropylaminophosphoramidite **III**,^15,28−30^ followed by the oxidation procedure described above (Scheme 2). Filtration on silica gel immediately followed by deprotection of the 2′-*O* and 3′-*O* TBS groups gave prodrug **5** in high purity and sufficient yields.

**Scheme 3:**
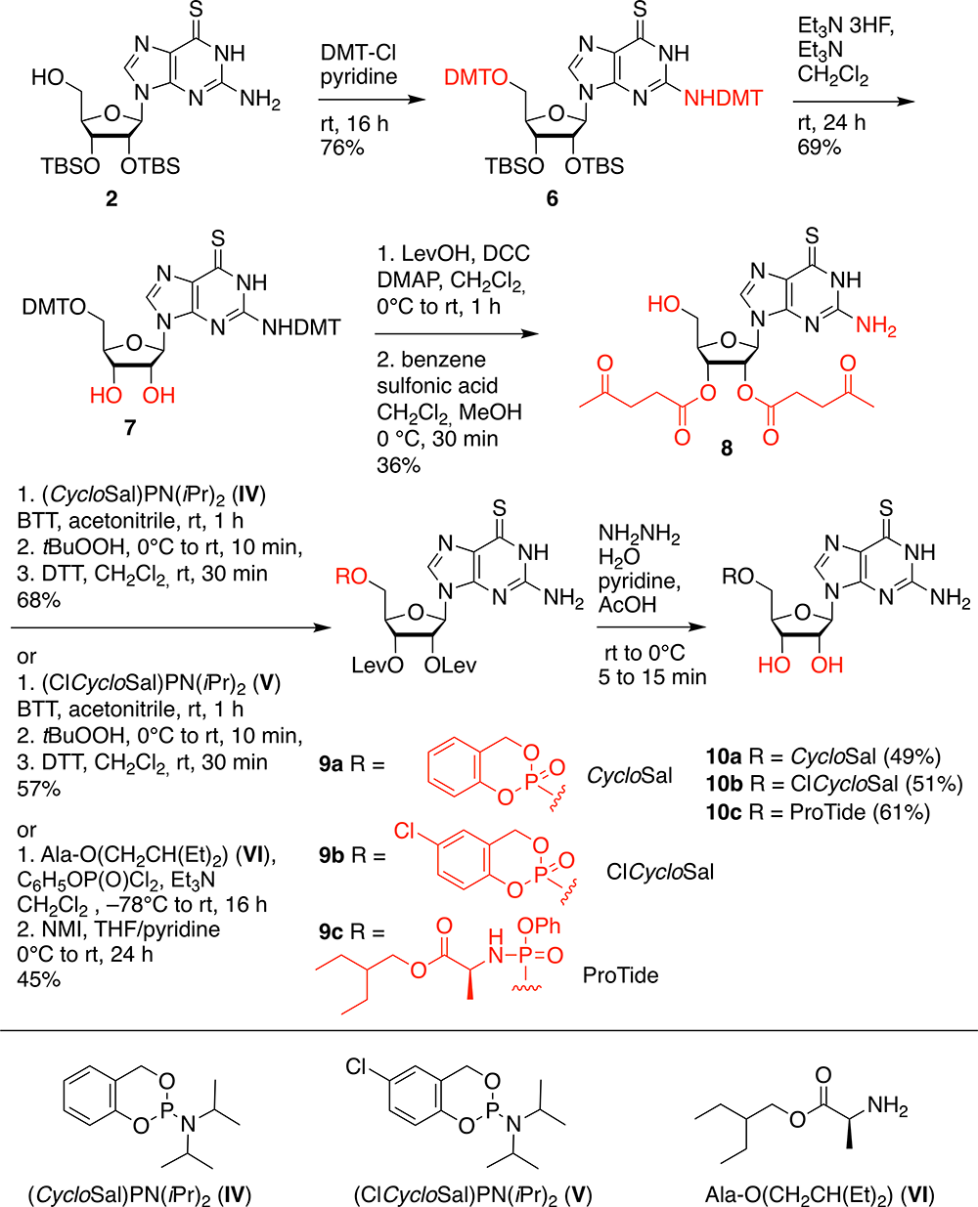
Synthesis of *Cyclo*Sal, Cl*Cyclo*Sal, and ProTide 6sGMP Prodrugs **10a**–**c** Abbreviations: *tert*-butyldimethylsilyl chloride (TBS-Cl), 4,4′-dimethoxytrityl chloride (DMTCl), levulinic acid (LevOH), *N*,*N*′-dicyclohexylcarbodiimide(DCC), 4-(*N*,*N*-dimethylamino)pyridine (DMAP), 5-benzylthio-*1H*-tetrazole (BTT), *N*-methylimidazole (NMI).

The synthesis of 6sG prodrugs with saligenyl (*Cyclo*Sal, Cl-*CycloS*al) and phosphoramidate (ProTide) compounds, **10a–c**, started from precursor **2** (Scheme 3). This compound was treated with 4,4′-dimethoxytrityl chloride and pyridine to yield the double tritylated compound **6**. Subsequent cleavage of the 2′-*O* and 3′-*O* TBS ethers with triethylamine trihydrofluoride (providing intermediate **7**) and re-protection of the 2′-*O* and 3′-*O* hydroxyl groups as levulinic acid esters followed by cleavage of the DMT groups resulted in the key precursor **8** for phosphorylation. This compound was reacted with either saligenyl-*N*,*N*-diisopropylaminophosphoramidite **IV**(31−33) or 5-chlorosaligenyl-*N*,*N*-diisopropylaminophosphoramidite **V**;^32−35^ then, oxidation using *tert*-butyl hydroperoxide at low temperature and immediate treatment with DTT to reduce eventually formed dimers and dispose any traces of oxidation reagents furnished nucleosides **9a** and **9b**, respectively. The actual prodrugs **10a** and **10b** were obtained using aqueous hydrazine in pyridine and acetic acid. The levulinyl esters were effectively cleaved leaving the sensitive saligenyl- and chlorosaligenyl moieties unharmed.

To obtain the 6sG ProTide nucleotide **10c** (Scheme 3), the necessary phosphorylation reagent was generated *in situ* in the presence of triethylamine by mixing phenyl dichlorophosphate and 2-ethylbutyl (*S*)-2-aminopropanoate hydrochloride **VI**.^36^ The phosphoryl chloride was then reacted with compound **8** to give compound **9c**; cleavage of the levulinic esters finally resulted in the target compound **10c**.

### Proliferation Inhibitory Potential of 6sG Monophosphate Prodrugs

**Figure 3:**
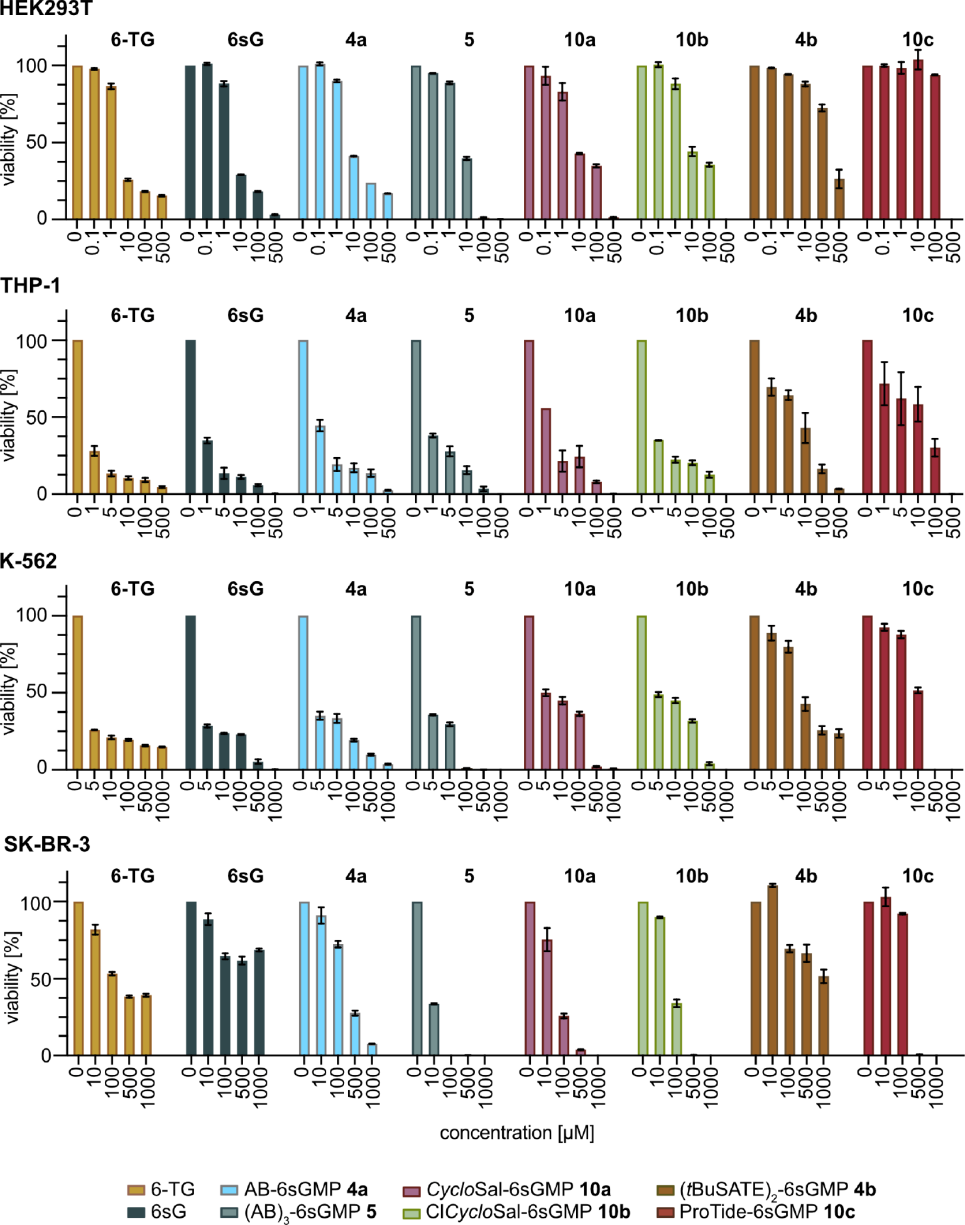
Analysis of the antiproliferative effects of 6-TG, 6sG, and different 6sGMP prodrugs. HEK293T, THP-1, K-562, and SK-BR-3 cells were incubated with different concentrations of the indicated drugs for 48 h, and viability was determined using the CellTiter Glow 2.0 assay. Mean ± SEM of two experiments with 3 replicates each is shown.

To investigate how the derivatized 6sGMP prodrugs compare to 6-TG (base) and 6sG (nucleoside) with respect to antiproliferative activity, we determined EC50 (half maximal effective concentration) after 48 h treatment of HEK293T cells using the CellTiter-Glo Assay (Promega). The results show that 6-TG and 6sG were most effective with EC50 of 3.6 and 4.7 μM, respectively (Table 1, Figure 3). The AB- and *Cyclo*Sal-derivatized 6sGMP prodrugs (**4a**, **5**, **10a**,**b**) had slightly higher EC50 values, while (*t*BuSATE)_2_ and ProTide prodrugs (**4b**, **10c**) were essentially ineffective (Table 1). These data show that in HEK293T cells, providing 6sGMP instead of thiopurine base or nucleoside offers no further advantage. These results are not unexpected because HEK293 cells have the highest *HGPRT* expression compared to other cell lines (The Human Protein Atlas; https://www.proteinatlas.org/), which should facilitate the conversion of the thiopurine base into the active 6-TGN resulting in high sensitivity toward the drug. Therefore, we tested three cell lines that exhibited low expression of *HGPRT* according to The Human Protein Atlas reasoning that under these conditions, a positive effect (if any) of the 6sGMP prodrugs might become apparent. The THP-1 and K-562 cell lines originate from acute monocytic leukemia and chronic myeloid leukemia, respectively, and display *HGPRT* levels that are in the lower half of the expression range of the 69 cell lines available at the Human Protein Atlas website. Consistent with the reported hypersensitivity of myeloid cells to thiopurine treatment,^6^ incubation of both cell lines with 6-TG and 6sG revealed the ready response of the cells with EC50 of 0.3 and 0.4 μM for THP-1 and 0.7 and 1.3 μM for K-562 (Figure 3; Table 1). The inhibition of THP-1 cell viability by the 6sGMP prodrugs was comparable to that of 6-TG, except for (*t*BuSATE)_2_- and ProTide-6sGMP (Table 1). Interestingly, ∼15% of K-562 cells survived treatment with the highest tested concentration of 6-TG (Figure 3), yet when we treated K-562 cells with the 6sGMP prodrugs, we observed that AB-, (AB)_3_-, *Cyclo*Sal-, and Cl*Cyclo*Sal-6sGMP (**4a**, **5**, **10a**,**b**) effectively inhibited the resistant population at higher concentrations while being slightly less effective than 6-TG at lower concentrations (Figure 3, Table 1). ProTide-6sGMP (**10c**) also killed the cells when administered at high concentration but showed reduced efficacy at lower concentrations, while the (*t*BuSATE)_2_-derivative (**4b**) again was ineffective (Figure 3).

**Table 1:** EC50 Values Calculated from Proliferation Data Shown in Figure 3^a^

	*EC50 [μM]*			
	HEK293T	K-562^b^	THP-1^b^	SK-BR-3
6-TG	3.6 ± 0.2	0.74 ± 0.07	0.27 ± 0.01	nd
6sG	4.7 ± 0.6	1.3 ± 0.3	0.35 ± 0.05	nd
AB-6sGMP **4a**	6.9 ± 0.5	2.3 ± 0.4	0.67 ± 0.10	201 ± 39
(AB)_3_-6sGMP **5**	6.7 ± 0.4	2.6 ± 0.5	0.34 ± 0.05	3.5 ± 0.9
*Cyclo*Sal-6sGMP **10a**	14 ± 5	7.2 ± 2.6	1.4 ± 0.2	25 ± 4
Cl*Cyclo*Sal-6sGMP **10b**	15 ± 6	5.6 ± 1.4	0.40 ± 0.07	42 ± 8
(*t*BuSATE)_2_-6sGMP **4b**	246 ± 18	62 ± 11	8.4 ± 2.9	nd
ProTide-6sGMP **10c**	nd	92 ± 10	33 ± 8	nd

a nd, not determined

b Additional concentrations (0.1, 1, 5 μM) were measured when necessary for calculation of EC50 values.

We then examined the effects of 6sGMP prodrugs in the breast cancer cell line SK-BR-3. These cells are among the cell lines with the lowest expression of *HGPRT* (https://www.proteinatlas.org/). We found that about 40% of SK-BR-3 cells were resistant to 6-TG even at 1 mM concentration. Remarkably, resistance was even higher when 6sG was administered (Figure 3), suggesting that low expression levels of *HGPRT* might impede the metabolization of 6sG to 6sGMP. In striking contrast, no resistant cells were observed with the 6sGMP prodrug derivatives containing AB, *Cyclo*Sal, and ProTide masking groups (**4a**, **5**, **10a**,**b**,**c**) (Figure 3). The strongest antiproliferative effect was displayed by (AB)_3_-6sGMP (**5**) with EC50 of 3.5 μM (Table 1). By contrast, (*t*BuSATE)_2_-6sGMP (**4b**) showed poor efficacy at all concentrations (Figure 3).

The fact that most of the 6sGMP prodrugs had higher EC50 values in susceptible cells, such as HEK293T cells, than 6-TG or 6sG can most likely be explained by the requirement for removal of the masking groups on the 5′-*O*-monophosphate to release the active 6sGMP. This is commonly achieved by cellular esterases^37^ and may remain incomplete, resulting in lower effective intracellular concentrations. Alternatively, or in addition, cellular uptake of the derivatized 6sGMP may be compromised by the masking groups. It is often proposed that the protection groups facilitate permeation of mono/di/triphosphate nucleoside prodrugs through the cell membrane due to an increase in hydrophobic character.^12^ However, very few experimental facts exist about how nucleoside monophosphate prodrugs enter the cell.^11^ It is possible that they require membrane transporters responsible for the uptake of thiopurines^38^ or other ABC transporters.^39,40^ The masking groups might weaken the interaction with those transporters, consequently again leading to lower effective concentrations inside the cell.

Taken together, our results clearly show that 6sGMP prodrugs, in particular those involving AB and *Cyclo*Sal masking groups (**4a**, **5**, **10a**,**b**), are very effective against cells with decreased sensitivity toward conventional thiopurine treatment.

### Correlation of Antiproliferative Effect with *HGPRT* Expression Levels

**Figure 4:**
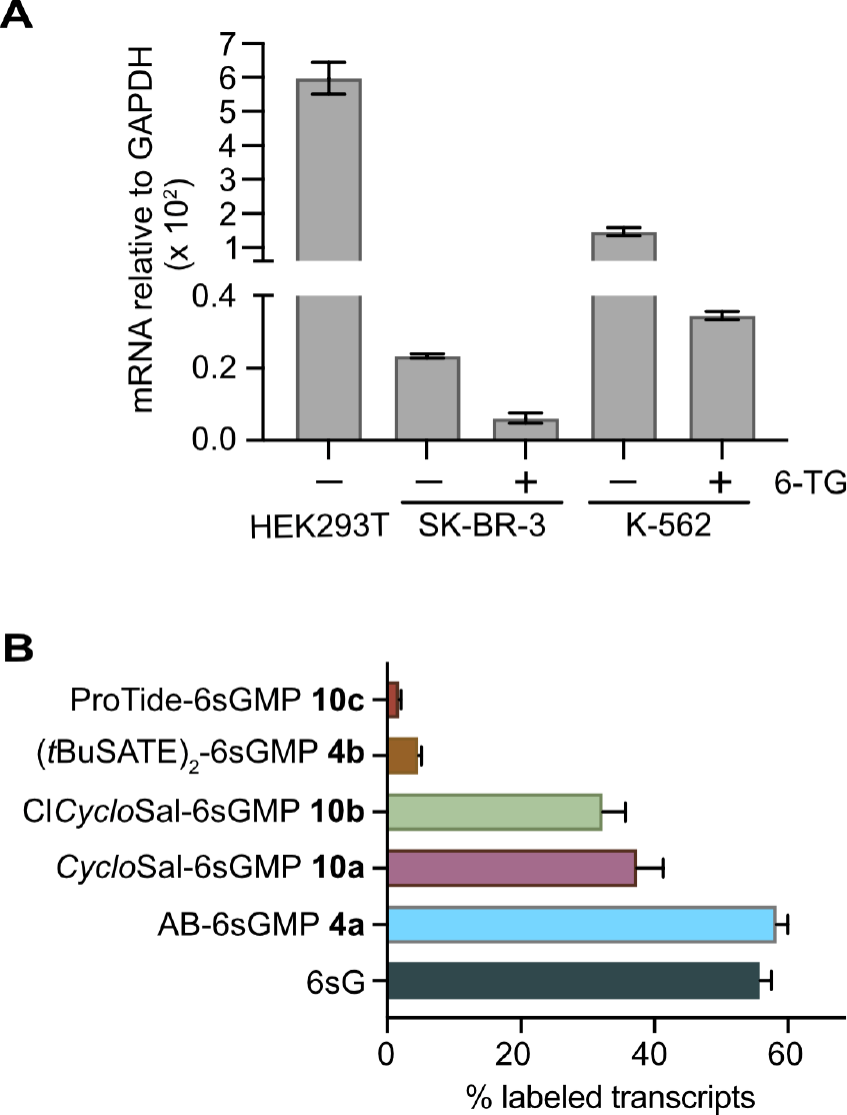
*HGPRT* expression in different cell lines and 6sG incorporation into RNA. (A) qPCR analysis of *HGPRT* expression in different cell lines. Cells were treated or not with 1 mM 6-TG for 48 h followed by RNA extraction, reverse transcription, and qPCR analysis. *GAPDH* served as internal reference. Mean ± SEM 2^–Δ*CT*^ values of two experiments are shown. (B) 6sG incorporation into RNA is related to the antiproliferative effects of thioguanosine prodrugs. HEK293T cells were treated with 100 μM of the indicated 6sGMP prodrugs for 16 h followed by RNA extraction, TUC treatment, and amplicon sequencing of *p21*. Labeling efficiency was calculated and corrected for random mutation using values derived from the DMSO control sample. Mean ± SD is shown (*N* = 2, 3 replicates each).

To better understand the molecular links between responsiveness of cells to 6-TG and purine metabolizing enzymes, we examined *HGPRT* expression levels. To this end, RT-qPCR was performed on K-562 and SK-BR-3 cells with and without treatment with 1 mM 6-TG for 48 h. Untreated HEK293T cells were used for reference. Our results confirmed the previously reported (www.proteinatlas.org) differences in *HGPRT* expression between HEK293T, K-526, and SK-BR-3 cells (Figure 4A). Intriguingly, SK-BR-3 and K-562 cells surviving 6-TG exhibited a further reduction of *HGPRT* transcript levels (Figure 4A). These results indicate that, particularly in SK-BR-3 cells, *HGPRT* availability might be a key factor affecting susceptibility of these cells to thiopurines, and they support the idea that the improved efficacy of 6sGMP prodrugs is due to a bypass of the *HGPRT* metabolization step.

### 6sGMP Prodrugs Can Be Converted to Bioactive Nucleotides and Incorporated into RNA

To examine if the administration of 6sGMP prodrugs leads to the formation of bioactive 6sGTP, we analyzed the extent of 6sGTP incorporation into mRNA using the recently developed TUC-seq DUAL method.^41^ Thus, 6sG nucleotides integrated into RNA during transcription can be detected by treatment of the isolated total RNA with a combination of OsO_4_, NH_4_Cl, and hydrazine, resulting in the conversion of 6sG to 6-hydrazino-2-aminopurine that is read as an adenine by reverse transcriptase resulting in a G-to-A mutation. The mutation signature can be detected by amplicon sequencing or RNA-seq.^42^ We tested a selection of 6sGMP prodrugs along with the 6sG nucleoside in HEK293T cells applying short and long-term labeling with 100 μM drug concentration. [Note that (AB)_3_-6sGMP (**5**) was not tested because the drug was not available at the time of analysis]. TUC-seq was first performed on *p21* mRNA after 16 h of drug incubation. The results show that AB-, *Cyclo*Sal-, and Cl*Cyclo*Sal-6sGMP (**4a**, **10a**,**b**) yielded high levels of labeled transcripts that were comparable to (AB **4a**) or slightly lower (*Cyclo*Sal **10a**, Cl*Cyclo*Sal **10b**) than 6sG (Figure 4B). On the other hand, ProTide- and *t*BuSATE -modified 6sGMP (**10c**, **4b**) were very poorly incorporated (Figure 4B). These results are in excellent agreement with the observed efficacies of the different prodrugs in the cell proliferation tests (Figure 3, Table 1).

We also sought to determine how RNA labeling dynamics of the 6sGMP prodrugs compares to that of the 6sG nucleoside. Thus, we performed TUC-seq on HEK293T cells that were treated for 2 h with 100 μM of each drug. The high turn-over mRNA *cyclin T1* (CCNT1), the more stable *p21* and also the mitochondrial transcript *ND1* were examined. While we found about 2.5 to 4.0% labeled transcripts when cells were treated with 6sG, none of the 6sGMP prodrugs showed appreciable labeling efficiency (Figure S1), suggesting that the availability of 6sGMP released from the masking groups is a slow process taking >2 h.

Taken together, we find that ProTide- and (*t*BuSATE)_2_-6sGMP (**10c**, **4b**) show low abundance in RNA after 16 h of labeling, indicating that uptake or release of the active 6sGMP is less efficient than for the other 6sGMP prodrugs. This is consistent with the reduced antiproliferative effects that we observed for both compounds. By contrast, AB-, *Cyclo*Sal-, and Cl*Cyclo*Sal-6sGMP (**4a**, **5**, **10a**,**b**) can be efficiently converted into the bioactive 6sG nucleotides followed by incorporation, e.g., into RNA (Figure 4), resulting in antiproliferative effects similar to those of 6-TG and 6sG in HEK293T cells and superior efficacy with thiopurine-resistant cell populations, such as the SK-BR-3 cell line. Thus, these results strongly suggest that the mode of action of these prodrugs is similar to that of the common thiopurines.

The results from our study also support TUC-seq DUAL^41^ as a potential new diagnosis tool. The success of thiopurine therapy is often measured by determining the level of 6-thioguanosine nucleotides by HPLC albeit without distinction between the different phosphorylated forms of 6sG nucleotides.^43,44^ To detect 6sGTP (considered to be the biologically most active form), liquid chromatography-mass spectrometry is required.^38^ The use of TUC-seq DUAL to measure the amount of 6sG that is incorporated into RNA might present an interesting alternative method in that it is able to determine the biologically active form of 6sG, requires small amounts of biological material, and uses standard equipment of a modern diagnostic laboratory.

## Conclusions

In this study, we developed robust synthetic routes toward 6-thioguanosine derivatives with 5′-*O*-monophosphate prodrug patterns (**4**, **5**, **10**) by mastering reaction conditions and protection concepts that preserve the oxidation-sensitive 6-thio moiety during the conversion of the P(III) intermediates into the corresponding P(V) products. Our cell proliferation tests demonstrate that some of the monophosphate prodrugs (**4a**, **5**, **10a**,**b**) exhibit similar efficacies as conventional thiopurine prodrugs in susceptible cell lines and have superior antiproliferative potential for cells resistant to thiopurine treatment. We show that prodrug treatment leads to efficient incorporation of 6sG into newly synthesized RNA, demonstrating that 6sGMP can be released inside the cells and successfully metabolized to the respective triphosphate nucleotide. Furthermore, we show that the endogenous level of the key metabolizing enzyme *HGPRT* is related to cellular resistance to thiopurine bases and nucleosides, providing a molecular explanation for the increased efficacies of 6sGMP prodrugs by bypassing the critical phosphoribosyl transfer step. Future studies are needed to examine specific aspects of pharmacokinetics and pharmacodynamics of 6sGMP prodrugs, such as oral bioavailability, tissue delivery, and stability, to determine 6sGMP prodrug potential for *in vivo* application. It is encouraging in this regard that several of the promising prodrug patterns used in this study (AB, *Cyclo*Sal, ProTide) have been successfully employed in clinical practice.^12,45−48^

Due to their relative ease of administration, their efficacy, and their cost effectiveness, thiopurines are in widespread clinical use even in the era of biological therapies and in spite of adverse reactions and resistance associated with thiopurine therapy. Hence, 6sG monophosphate prodrugs may provide a promising means to combat thiopurine resistance in the treatment of inflammatory diseases and cancer.

## Experimental Section

### Synthesis of 6sG Prodrugs

Synthetic procedures and analysis data for the synthesis of compounds and reagents are described in the Supporting Information. All compounds synthesized are 95% pure by NMR analysis, and all NMR spectra are depicted in the Supporting Information.

### Cell Culture

Human embryonic kidney cell line HEK293T, human myeloid leukemia cell line K-562, human monocytic leukemia cell line THP-1, and human breast cancer cell line SK-BR-3 were used in this study. All cell lines were maintained at 37 °C and 5% CO_2_. HEK293T cells were cultivated in DMEM/Ham’s F-12 medium with 10% fetal calf serum and GlutaMAX (PAN Biotech, Gibco). K-562 and THP-1 cells were grown in RPMI 1640 media (Corning) supplemented with 10% fetal calf serum, GlutaMAX, and penicillin–streptomycin (PAN Biotech, Gibco Sigma). For THP-1 cells, 1 mM sodium pyruvate (Sigma) and 0.05 mM 2-mercaptoethanol (Gibco) were supplied in addition. SK-BR-3 cells were cultivated in McCoy’s 5A medium with 10% fetal calf serum, l-glutamine, and penicillin–streptomycin (PAN Biotech).

### Cell Proliferation Test

Cells were seeded in triplicate at a density of 7 × 10^3^ per well into 96 well plates (10^5^ for THP-1). After 24 h of incubation at 37 °C and 5% CO_2_, the prodrugs were added to achieve 0.1, 1, 5, 10, 100, 500, and 1000 μM final concentration. DMSO was used as a mock control. After 48 h treatment, cell proliferation was measured with the CellTiter-Glo 2.0 Assay (Promega) according to the manufacturer’s instructions. Absolute EC50 was calculated using the nonlinear regression function of GraphPad Prism 9.

### Metabolic Labeling and 6sGTP Incorporation Analysis (TUC-Seq DUAL)

1.5 × 10^6^ HEK293T cells were seeded into 3 cm round cell culture dishes and incubated for 6 h at 37 °C and 5% CO_2_ in culture medium. The medium was replaced with a culture medium supplemented with 100 μM 6sG,^41^ 100 μM of the different synthesized 6sGMP prodrugs or vehicle (DMSO). Cells were incubated for 2 h or overnight (16 h) and subsequently harvested. Total RNA was isolated from labeled and DMSO-treated cells using the innuPREP RNA Mini Kit 2.0 (Analytik Jena) according to the manufacturer’s instructions. RNA (3 μg) was incubated with 0.45 mM OsO_4_ and 180 mM NH_4_Cl in a total volume of 22 μL for 2 h at 40 °C. The reaction was stopped by precipitation with 6 volumes of ice-cold ethanol and 2 volumes precipitation buffer (185 mM NaOAc pH 5.2 and 0.25 mg/mL glycogen). The RNA pellet was dissolved in 15 μL of H_2_O and mixed with 5 μL of hydrazine solution (0.5 M, 5 mM EDTA, 0.5 M Tris, pH 8.11) to a final concentration of 125 mM hydrazine and incubated for 2 h at 40 °C. The RNA solution was again precipitated with precipitation solution and ethanol before dissolving the pellet in 10 μL of nuclease free water.^41^

### Amplicon Sequencing and Data Analysis

TUC-treated RNA was reverse-transcribed with GoScript Reverse Transcriptase (Promega) with random hexamer primers according to the manufacturer’s instructions. Amplicons of *p21* (346 bp), *CCNT1* (296 bp), and *ND1* (353 bp) were generated by PCR with barcoded primers, purified from 1% agarose gels and pooled at equimolar ratio (primers are listed in Table S1). Library preparation from the amplicon pool and sequencing using the Illumina HighSeq platform was performed by Eurofins (Ebersberg, Germany). Processing of raw reads, determination of G-to-A conversion frequency and calculation of incorporation efficiency was performed exactly as described^41^ with the addition that we filtered all reads that had >1 unspecific G conversion (G → C|T).

### RT-qPCR

Total isolated RNA was reverse-transcribed with GoScript Reverse Transcriptase (Promega) and subjected to real-time qPCR using Luna qPCR mix (NEB) with primers for human *HGPRT* and glyceraldehyde 3-phosphate dehydrogenase (GAPDH) as a reference (Table S1). 2^–Δ*CT*^ values were calculated and plotted.

## Supporting Information Available

Synthetic procedures and spectroscopic data for all compounds synthesized (PDF)
Molecular formula strings (CSV)

## Abbreviations

TUC-seq: thiouridine-to-cytidine conversion sequencing
