# Supplementary material for: 6-Thioguanosine Monophosphate Prodrugs Display Enhanced Performance against Thiopurine-Resistant Leukemia and Breast Cancer Cells
Source: J Med Chem. 2022 Nov 14;65(22):15165–73. doi: 10.1021/acs.jmedchem.2c01010 (PMC9706553; doi:10.1021/acs.jmedchem.2c01010)
Supplement: Supplementary file 1 — jm2c01010_si_001.pdf [file jm2c01010_si_001.pdf]

Supporting Information

6-Thioguanosine monophosphate prodrugs display enhanced performance against thiopurine-resistant leukemia and breast cancer cells

Sarah Moreno,† Magdalena Fickl,‡ Ingo Bauer,‡ Melanie Brunner,‡ Anna Rázková,† Dietmar Rieder,§ Isabel Delazer,‡ Ronald Micura,† Alexandra Lusser ‡**

† Institute of Organic Chemistry, Center for Molecular Biosciences Innsbruck, University of Innsbruck, Innrain 80-82, 6020 Innsbruck, Austria; ‡ Institute of Molecular Biology, Biocenter, Medical University of Innsbruck, Innrain 80-82, 6020 Innsbruck, Austria; § Institute of Bioinformatics, Biocenter, Medical University of Innsbruck, Innrain 80-82, 6020 Innsbruck, Austria.

* Email: alexandra.lusser@i-med.ac.at, ronald.micura@uibk.ac.at

Contents

1. Chemical synthesis S2

2. Synthesis of 4-acetyloxybenzyl (AB)- and *S*-pivaloyl-2-thioethyl (*t*BuSATE)$_2$ 6sG 5′-*O*-monophosphates (4a, 4b) S3

2.1. Synthesis of 2′,3′,5′-tri-*O*-(*tert*-butyldimethylsilyl)-6-thioguanosine **(1)** S4

2.2. Synthesis of 2′,3′-di-*O*-(*tert*-butyldimethylsilyl)-6-thioguanosine **(2)** S6

2.3. Synthesis of (4-acetyloxybenzyl)-((9*H*-fluoren-9-yl)methyl)-5′-*O*-(2′,3′-di-*O*-(*tert*-butyldimethylsilyl)-6-thioguanosinyl) phosphate **(3a)** S8

2.4. Synthesis of bis(*S*-pivaloyl-2-thioethyl)-5′-*O*-(2′,3′-di-*O*-(*tert*-butyldimethylsilyl)-6-thioguanosinyl) phosphate **(3b)** S11

2.5. Synthesis of (4-acetyloxybenzyl)-5′-*O*-(6-thioguanosinyl) phosphate triethylammonium salt **(AB-6sGMP, 4a)** S14

2.6. Synthesis of bis(*S*-pivaloyl-2-thioethyl)-5′-*O*-(6-thioguanosinyl) phosphate **((*t*BuSATE)$_2$-6sGMP, 4b)** S17

3. Synthesis of bis(4-acetyloxybenzyl)-5′-*O*-(*S*^{6}-(4-acetyloxybenzyl) guanosinyl) phosphate (5) S20

4. Synthesis of *cyclo*Sal-6sG, Cl*cyclo*Sal-6sGMP and ProTide-6sGMP (10a-c) S24

4.1. Synthesis of 2′,3′-di-*O*-(*tert*-butyldimethylsilyl)-*N*^{2},5′-*O*-di-(4,4′-dimethoxytrityl)-6-thio guanosine (**6**) S25

4.2. Synthesis of *N*^{2},5′-*O*-di-(4,4′-dimethoxytrityl)-6-thioguanosine (**7**) S27

4.3. Synthesis of 2′,3′-di-*O*-levulinoyl-6-thioguanosine (**8**) S29

4.4. Synthesis of *cyclo*saligenyl-5′-*O*-(2′,3′-di-*O*-levulinoyl-6-thioguanosinyl)phosphate (**9a**) S32

4.5. Synthesis of *cyclo*(5-chlorosaligenyl)-5′-*O*-(2′,3′-di-*O*-levulinoyl-6-thioguanosinyl) phosphate (**9b**) S35

4.6. Synthesis of 2-ethylbutyl-2-(5′-*O*-(2′,3′-di-*O*-levulinoyl-6-thioguanosinyl) (phenoxy) phosphoryl) alaninate (**9c**) S38

4.7. Synthesis of *cyclo*saligenyl-5′-*O*-(6-thioguanosineyl) phosphate (*cyclo*Sal-6sGMP, **10a**) S41

4.8. Synthesis of *cyclo*(5-chlorosaligenyl)-5′-*O*-(6-thioguanosinyl) phosphate (Cl*cyclo*Sal-6sGMP, **10b**) S44

4.9. Synthesis of 2-ethylbutyl-2-(5′-*O*-(6-thioguanosinyl)(phenoxy)phosphoryl) alaninate (ProTide-6sGMP, **10c**) S47

5. Figure S-1 S50

6. Table S-1 S51

7. References S52

1. Chemical synthesis

All reactions were carried out under argon gas atmosphere using absolute solvents. Solvents and other reagents were purchased from commercial suppliers in the highest available purity and were used without further purification. Acetonitrile and acetonitrile containing 0.3 M 5-(benzylthio)-1*H*-tetrazole (BTT) were dried over molecular sieves. Solutions of *tert*-butyl hydroperoxide (5.0-6.0 M in decane) were purchased from Sigma Aldrich and diluted with dry toluene to a final concentration of 0.5-1.0 M which was dried over molecular sieves before its use in chemical reactions. Reagents **I-VI** were prepared according to Moreno *et al.*[1]

Reaction progress was monitored via Polygram Sil G/UV254 (Marchery-Nagel) thin layer chromatography plates. Non-fluorescent compounds were visualized using an I2-chamber. Silica gel 60 (70-230 mesh; Fluka) was used for column chromatography. Identity and purity of the synthesized compounds was analyzed by NMR spectroscopy and mass spectrometry. NMR spectra were recorded on 300 MHz Bruker Avance spectrometer, 400 MHz Bruker Avance 4 Neo spectrometer and 600 MHz Avance II+ spectrometer and the reported chemical shifts were referenced to deuterated solvents (^{1}H: $CDCl_3$ = 7.26 ppm, d_6-DMSO = 2.50 ppm, ^{13}C: $CDCl_3$ = 77.16 ppm, d_6-DMSO = 39.52 ppm). Prodrug candidates were additionally characterized via ^{31}P-NMR which shifts are relative to external 85% phosphoric acid. ^{1}H-^{1}H-COSY-, ^{1}H-^{13}C-HSQCand ^{1}H-^{13}C-HMBC-experiments were recorded to enable signal assignment. High resolution mass spectrometry was carried out on a Thermo Scientific Q Exactive Orbitrap using an electrospray ionization system. Samples were dissolved in methanol and were measured in positive and negative ion mode with a spray voltage of 3.7 kV.

2. Synthesis of 4-acetyloxybenzyl (AB)- and *S*-pivaloyl-2-thioethyl (*t*BuSATE)$_2$ 6sG 5′-*O*-monophosphates (4a, 4b)

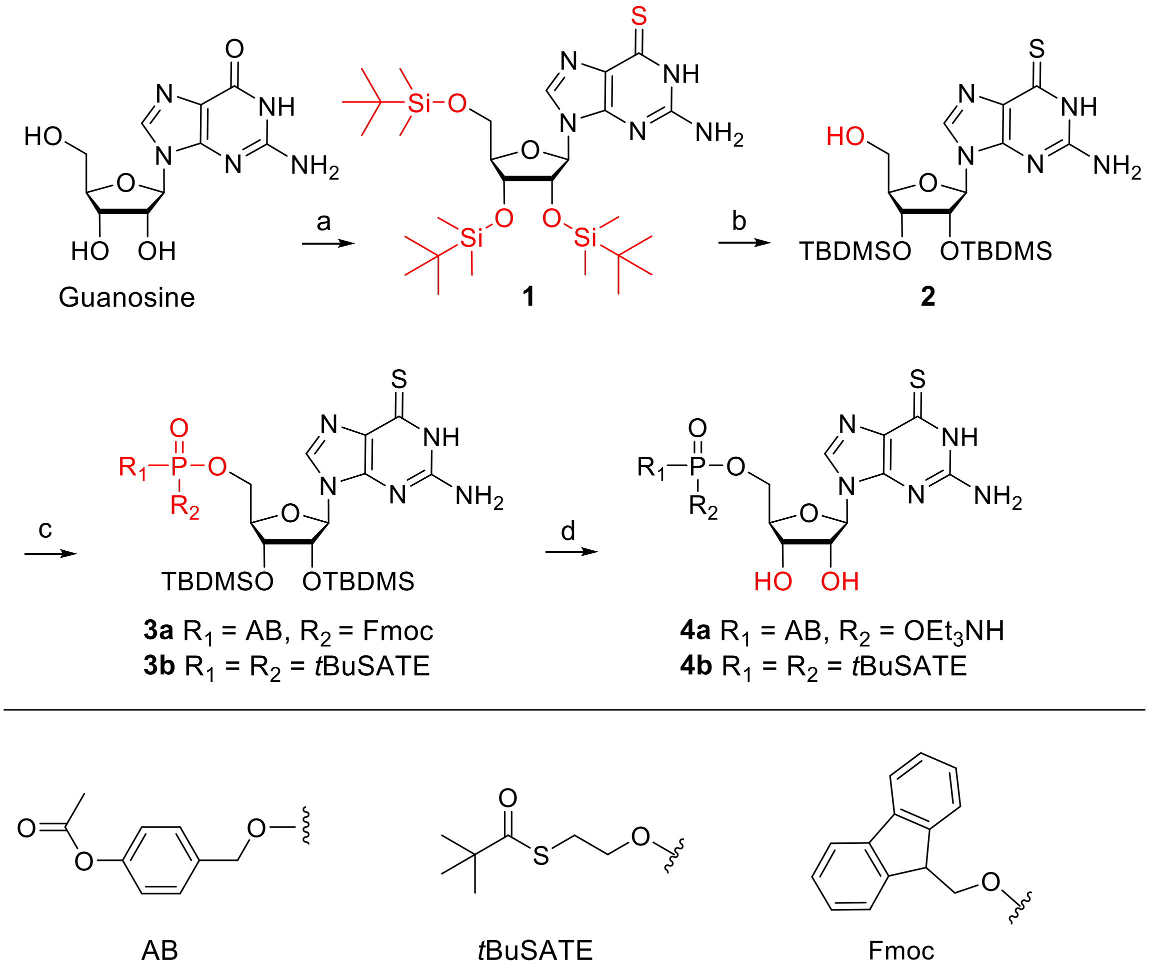

Scheme 1 Synthesis of AB-6sG **4a** & (*t*BuSATE)$_2$-6sG **4b**. **a)** i) 10.0 eq imidazole, 5.0 eq TBDMSCl, DMF, 16 h, RT; ii) 1.2 eq Lawesson reagent, toluene, 2 h, 100 °C, 95%. **b)** HOAc, H_2O, 22 h, 60 °C, 71%. **c)** i) for **3a:** 1.2 eq compound **I**; for **3b:** 1.2 eq compound **II**, 1.2 eq BTT, ACN, 60-70 min, RT; ii) 1.2 eq *t*BuOOH, 10 min, 0 °C-RT; iii) 50 mM DTT, CH_2Cl_2, 30 min, RT, 60% for **3a**, 74% for **3b**. **d)** 6.0 eq $Et_3N{\cdot}3HF$, 6.0 eq Et_3N, CH_2Cl_2, 16 h, RT, 98% for **4a**, 70% for **4b**. Overall yield: **4a:** 40%, **4b:** 35%.

2.1. Synthesis of 2′,3′,5′-tri-*O*-(*tert*-butyldimethylsilyl)-6-thioguanosine (1)[2,3]

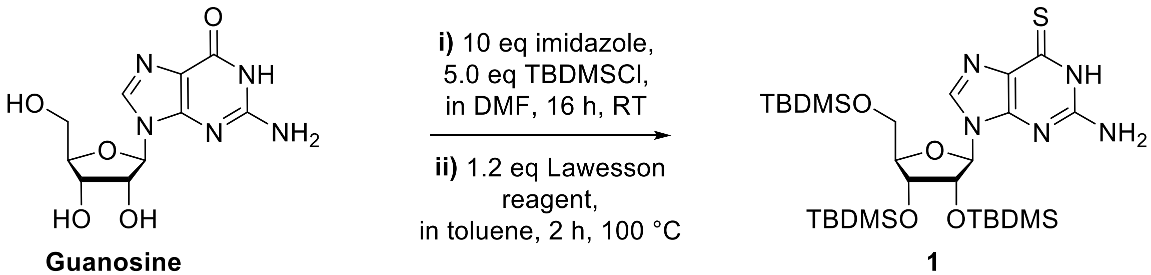

Guanosine **1**

Guanosine (2.280 g, 8.05 mmol) was suspended in *N,N*-dimethylformamide (20.0 ml) and was treated with imidazole (10.0 eq, 80.50 mmol, 5.480 g) and *tert*-butyldimethylsilyl chloride (5.0 eq, 40.25 mmol, 6.066 g). The suspension turned into an off-white solution within 1 h which was stirred at ambient temperature for 16 h. The reaction mixtures was diluted with ethyl acetate and was washed extensively with water and brine. The organic layer was dried over sodium sulfate, was concentrated, coevaporated trice with toluene and dried under high vacuum. The white foam was then dissolved toluene (160.0 ml) and Lawesson reagent (1.2 eq, 9.66 mmol, 3.907 g) was introduced. The reaction mixture was stirred 2 h at 100 °C followed by evaporation of the solvent and column chromatography (0-5% MeOH in CH_2Cl_2). Yield: 4.910 g as a yellow foam (95%). TLC (5% MeOH in CH_2Cl_2): 0.47. HR-ESI-MS (m/z): $[M+H]^+$ calculated for $[C_{28}H_{56}N_5O_4SSi_3]^+$: 642.3355; found: 642.3349. ^{1}H-NMR (d_6-DMSO, 400 MHz): δ -0.28, -0.07, 0.087, 0.096, 0.11 (18 H, 5x s, 6x CH_3 (TBDMS)); 0.73, 0.90 (27 H, 2x s, 3x *t*-Bu (TBDMS)); 3.71 (1 H, dd, $^2J_{HH}$ = 11.20 Hz, $^3J_{HH}$ = 3.34 Hz, CH_a (5′)); 3.86 (1 H, td, $^2J_{HH}$ = 11.29 Hz, $^3J_{HH}$ = 5.31 Hz, CH_b (5′)); 3.96 (1 H, triplettoid, $^3J_{HH}$ = 4.47 Hz, CH (4′)); 4.17 (1 H, d br., $^3J_{HH}$ = 3.75 Hz, CH (3′)); 4.57 (1 H, dd, $^3J_{HH}$ = 6.51 Hz, $^3J_{HH}$ = 4.63 Hz, CH (2′)); 5.73 (1 H, d, $^3J_{HH}$ = 4.65 Hz, CH (1′)); 6.80 (2 H, s br., NH_2); 8.07 (1 H, s, CH (8)); 11.96 (1 H, s, CH (8)) ppm. ^{13}C-NMR (d_6-DMSO, 101 MHz): δ -5.59, -5.48, -4.92, -4.86, -4.79 6x CH_3 (TBDMS); 17.42, 17.67, 17.93 3x C_q *t*-Bu (TBDMS); 25.38, 25.61, 25.75 2x *t*-Bu (TBDMS); 62.78 C (5′); 72.58 C (3′), 75.10 C (2′); 85.30 C (1′); 85.67 C (1′); 128.09 C (5); 138.57 C (8); 148.03 C (4); 153.08 C (2); 175.12 C (6)ppm.

^{1}H-NMR (400 MHz, d_6-DMSO) of compound **1**

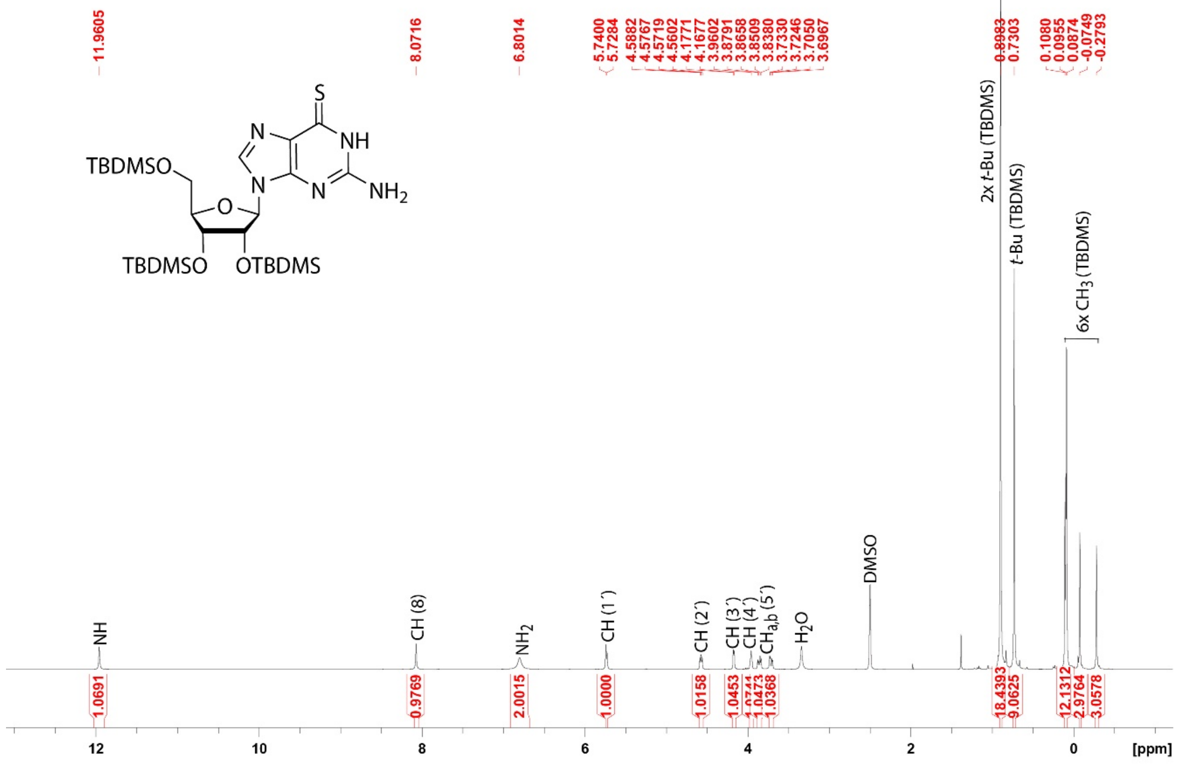

^{13}C-NMR (101 MHz, d_6-DMSO) of compound **1**

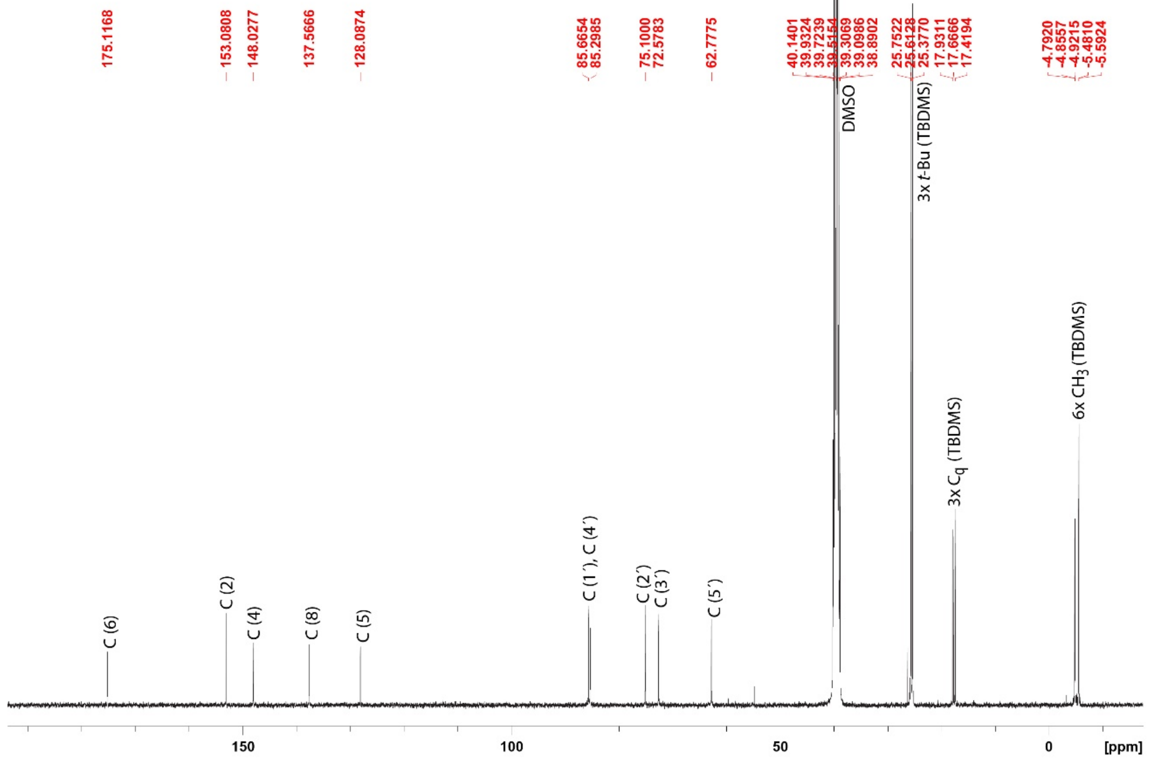

2.2. Synthesis of 2′,3′-di-*O*-(*tert*-butyldimethylsilyl)-6-thioguanosine (2)[4]

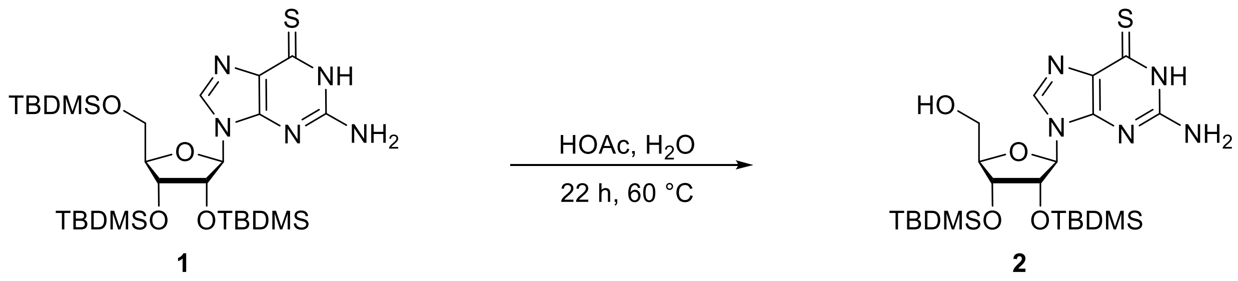

Compound **1** (4.910 g, 7.65 mmol) was dissolved in acetic acid (60.0 ml) and was treated with water (15.0 ml). The yellow suspension was stirred at 60 °C for 22 h whereas it turned into a yellow solution. Solvent was removed *in vacuo* and the remaining residue was coevaporated trice with ethanol. Column chromatography (0-4% MeOH in CH_2Cl_2) delivers compound **2** as a white powder. Yield: 2.869 g as a white powder (71%). TLC (5% MeOH in CH_2Cl_2): 0.41. HR-ESI-MS (m/z): $[M+H]^+$ calculated for $[C_{22}H_{42}N_5O_4SSi_2]^+$: 528.2491; found: 528.2483. ^{1}H-NMR (d_6-DMSO, 400 MHz): δ -0.30, -0.08, 0.09, 0.11 (12 H, 4x s, 4x CH_3 (TBDMS)); 0.73, 0.90 (18 H, 2x s, 2x *t*-Bu (TBDMS)); 3.56 (1 H, ddd, $^2J_{HH}$ = 11.73 Hz, $^3J_{HH}$ = 5.16 Hz, $^3J_{HH}$ = 3.95 Hz, CH_a (5′)); 3.67 (1 H, m, CH_b (5′)); 3.92 (1 H, triplettoid, $^3J_{HH}$ = 4.18 Hz, CH (4′)); 4.23 (1 H, d br., $^3J_{HH}$ = 4.21 Hz, CH (3′)); 4.67 (1 H, dd, $^3J_{HH}$ = 6.97 Hz, $^3J_{HH}$ = 4.53 Hz, CH (2′)); 5.18 (1 H, t, $^3J_{HH}$ = 5.31 Hz, OH (5′)); 5.73 (1 H, d, $^3J_{HH}$ = 7.09 Hz, CH (1′)); 6.80 (2 H, s br., NH_2); 8.17 (1 H, s, CH (8)); 11.96 (1 H, s, CH (8)) ppm. ^{13}C-NMR (d_6-DMSO, 101 MHz): δ -5.44, -4.78, -4.75, -4.70 4x CH_3 (TBDMS); 17.52, 17.76 2x C_q *t*-Bu (TBDMS); 25.48, 25.72 2x *t*-Bu (TBDMS); 61.08 C (5′); 72.95 C (3′), 74.98 C (2′); 85.67 C (1′); 86.71 C (1′); 128.28 C (5); 138.38 C (8); 148.05 C (4); 153.04 C (2); 175.12 C (6)ppm.

^{1}H-NMR (400 MHz, d_6-DMSO) of compound **2**

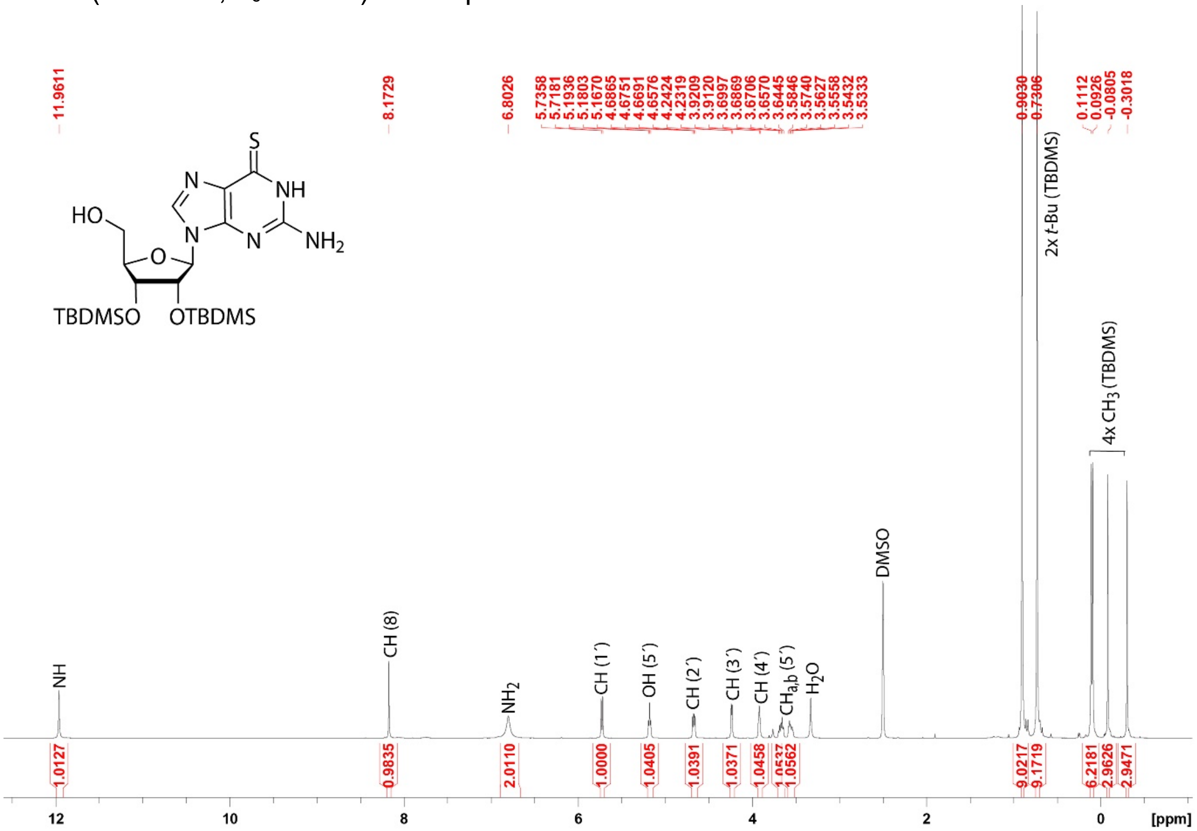

^{13}C-NMR (101 MHz, d_6-DMSO) of compound **2**

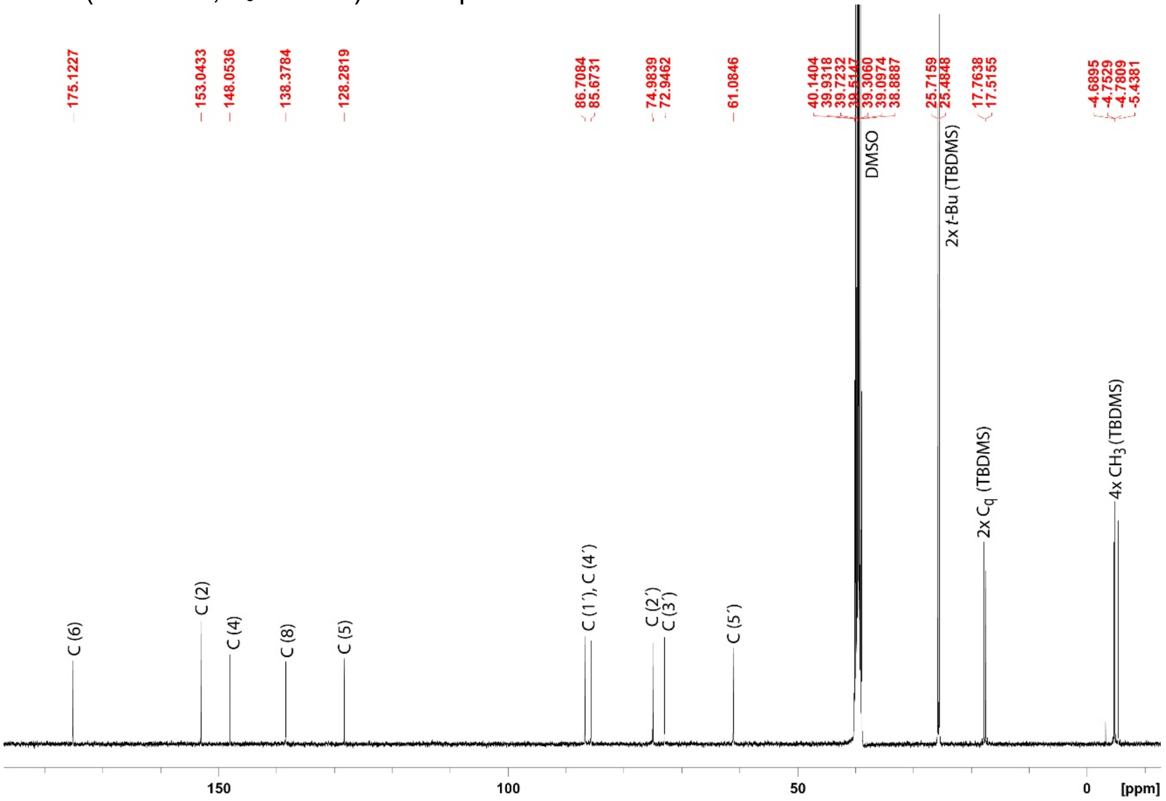

2.3. Synthesis of (4-acetyloxybenzyl)-((9*H*-fluoren-9-yl)methyl)-5′-*O*-(2′,3′-di-*O*-(*tert*-butyldimethylsilyl)-6-thioguanosinyl) phosphate (3a)

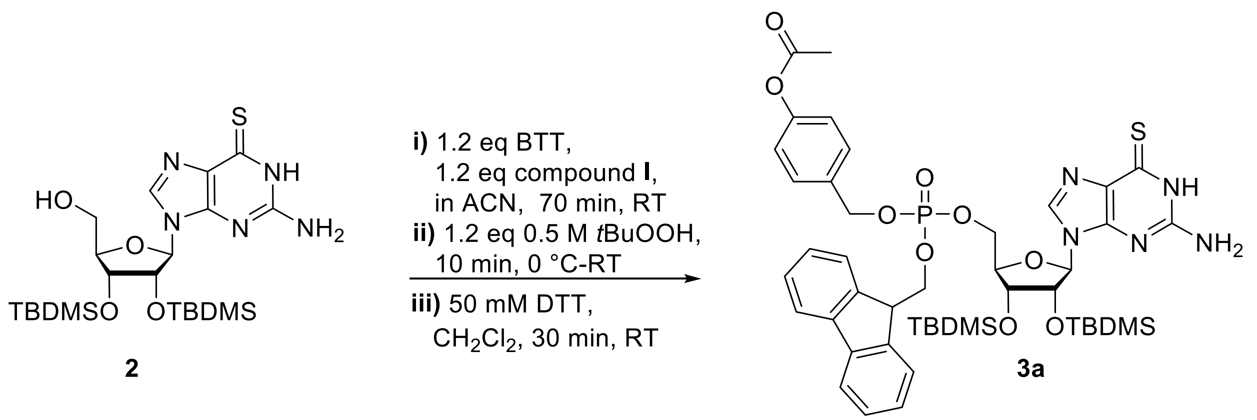

Compound **2** (97 mg, 0.18 mmol) was dried under high vacuum overnight. It was then suspended in acetonitrile (0.61 ml) and 0.3 M 5-(benzylthio)-1*H*-tetrazole in acetonitrile (1.2 eq, 0.22 mmol, 0.74 ml) was added. The suspension was then treated dropwise with compound **I**[1] (1.2 eq, 0.22 mmol, 108 mg) in acetonitrile (0.74 ml) over a period of 10 min and was subsequently stirred for 60 min. *Tert*-butyl hydroperoxide (in decane, 1.2 eq, 0.22 mmol, 0.44 ml 0.5 M in toluene) was added dropwise at 0 °C and the reaction mixture was stirred for 5 min at 0 °C and another 5 min at ambient temperature. Solvent was removed under reduced pressure and the crude product was dissolved in dichloromethane containing 50 mM DL-dithiothreitol (2.0 ml). After 30 min solvent was evaporated and the product was purified by flash chromatography (0-4% MeOH in CH_2Cl_2). Yield: 103 mg as a colorless oil (60%). TLC (5% MeOH in CH_2Cl_2): 0.44. HR-ESI-MS (m/z): $[M+H]^+$ calculated for $[C_{45}H_{61}N_5O_9PSSi_2]^+$: 934.3461; found: 934.3448. ^{1}H-NMR ($CDCl_3$, 400 MHz): δ -0.33, -0.27, -0.11, -0.08, 0.05, 0.06, 0.07, 0.08 (12 H, 4x s, 4x CH_3 (TBDMS)); 0.77, 0.79, 0.90, 0.91 (18 H, 2x s, 2x *t*-Bu (TBDMS)); 2.30, 2.33 (3 H, s, CH_3 (AB)); 4.10-4.46 (7 H, m, CH (3′), CH (Fmoc), CH (4′), CH_2 (5′), CH_2 (Fmoc)); 4.80-4.94 (3 H, m, CH (2′), CH_2 (AB)); 5.67 (1 H, 2x d, $CH_{a,b}$ (1′)); 5.80 (1 H, s br., $NH_{2,a}$); 6.09 (1 H, s br., $NH_{2,b}$); 6.99-7.76 (12 H, m, aromat. CH (AB+Fmoc)); 7.91, 7.94 (1 H, s, $CH_{a,b}$ (8)) ppm. ^{13}C-NMR ($CDCl_3$, 151 MHz): δ -5.05—4.30 4x CH_3 (TBDMS); 17.98, 18.00, 18.15, 18.17 2x C_q *t*-Bu (TBDMS); 21.27, 21.35 CH_3 (AB); 25.83, 25.85, 25.90, 25.92 2x *t*-Bu (TBDMS); 47.94-48.11 (2x d, CH (Fmoc)); 66.99-67.15 (2x d, CH (5′)); 68.93-69.11 (2x d, CH_2 (AB)); 69.69-69.90 (2x d, CH_2 (Fmoc)); 72.34, 72.57 C (3′), 73.38 C (2′); 83.53-83.93 (m, C (4′)); 89.54, 89.63 C (1′); 120.13, 120.24, 122.04, 122.10, 125.09-125.22

(2x d), 127.32-127.38, 127.97, 128.07, 128.17, 128.18, 128.83, 129.01, 129.25, 129.41 aromat. C (AB+Fmoc); 130.25, 130.28 C (5); 133.20 (d, $^3J_{CP}$ = 6.76 Hz, $C_{q,a}$ (AB); 133.64 (d, $^3J_{CP}$ = 5.42 Hz, $C_{q,b}$ (AB); 140.62, 140.91 C (8); 141.44, 141.48, 141.58, 141.59, 142.90, 142.96, 142.97, 143.04 C_q (AB+Fmoc); 147.24, 147.41 C (4); 150.70, 150.98 C_q (AB+Fmoc); 152.66, 152.70 C (2); 169.81, 171.22 C=O (AB); 175.48, 175.61 C (6) ppm. <u>^{31}P-NMR</u> ($CDCl_3$, 162 MHz): δ -1.82, -1.52 ppm

^{1}H-NMR (400 MHz, $CDCl_3$) of compound **3a**

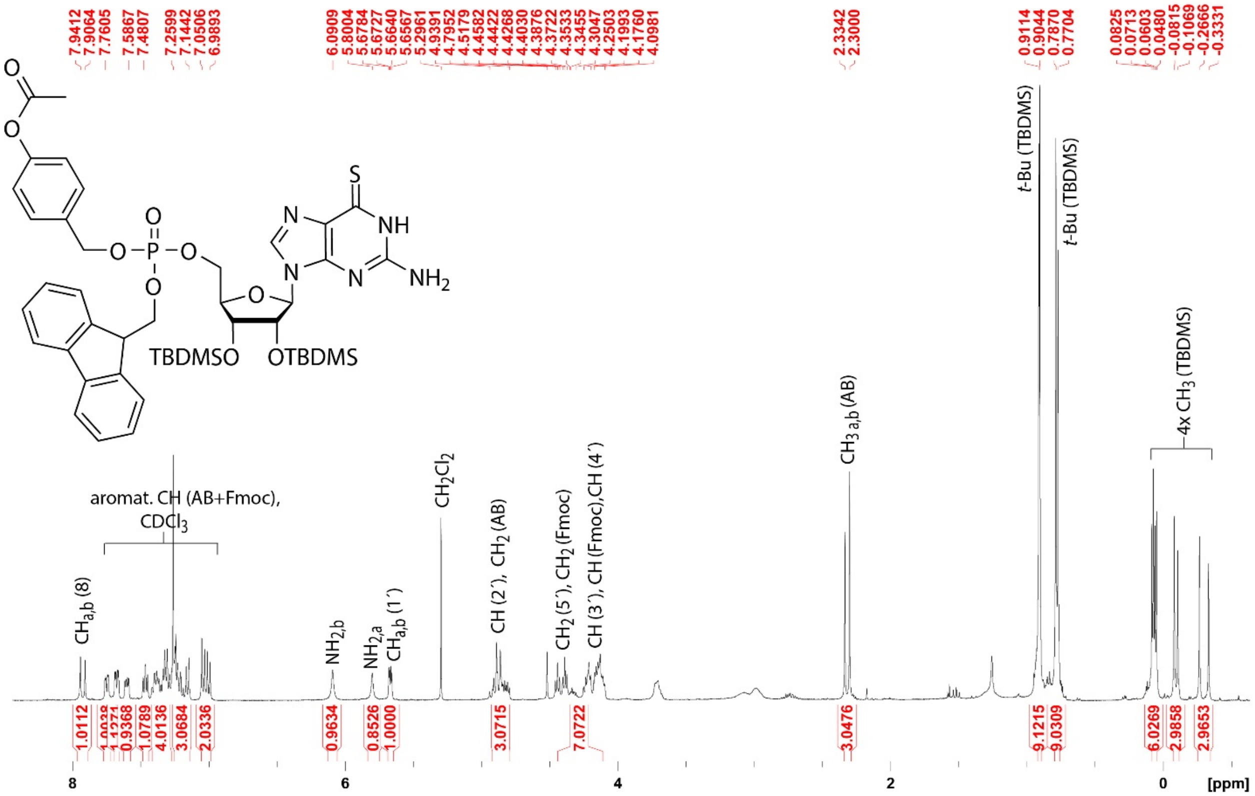

^{13}C-NMR (151 MHz, $CDCl_3$) of compound **3a**

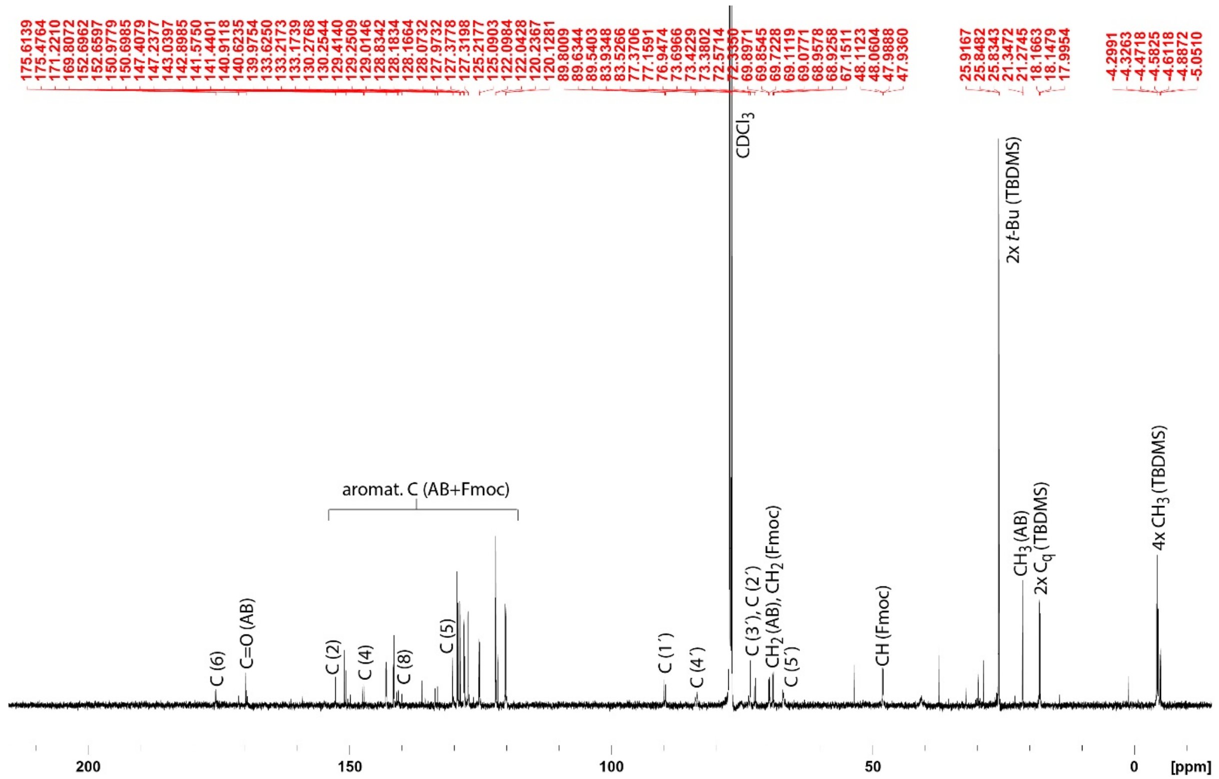

^{31}P-NMR (162 MHz, $CDCl_3$) of compound **3a**

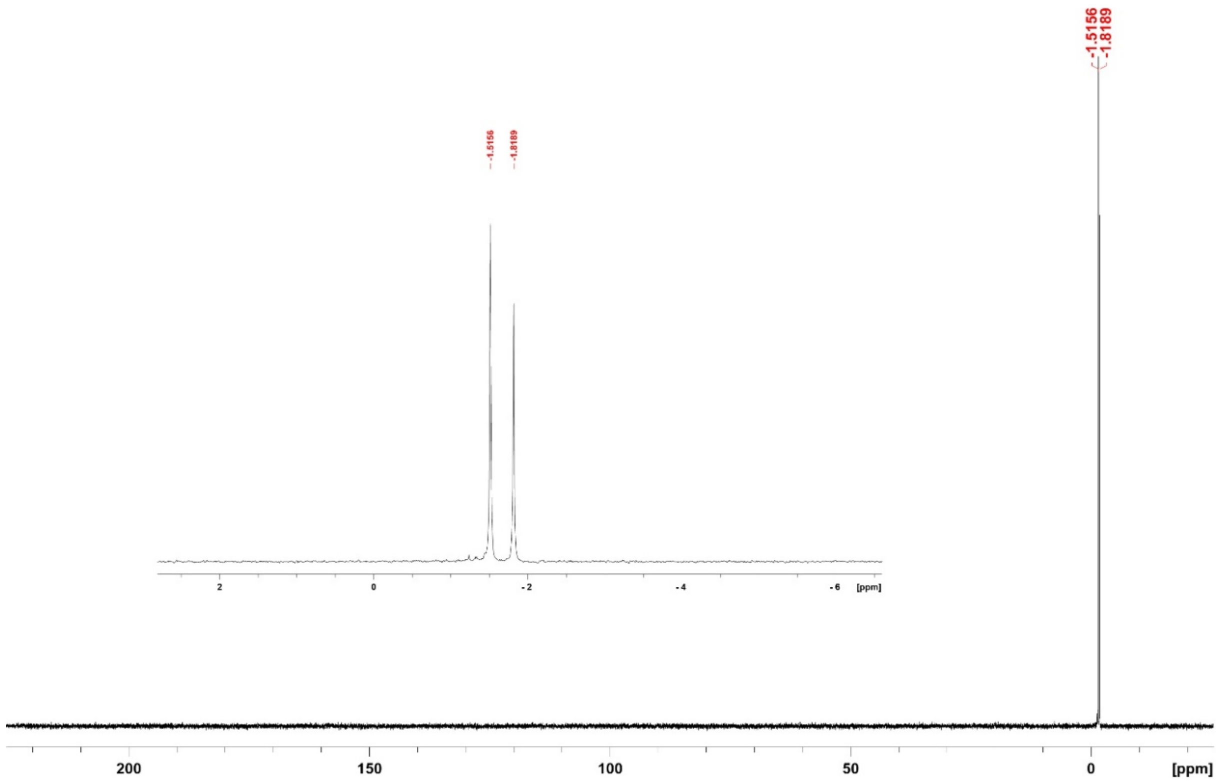

2.4. Synthesis of bis(*S*-pivaloyl-2-thioethyl)-5′-*O*-(2′,3′-di-*O*-(*tert*-butyldimethylsilyl)-6-thioguanosinyl) phosphate (3b)

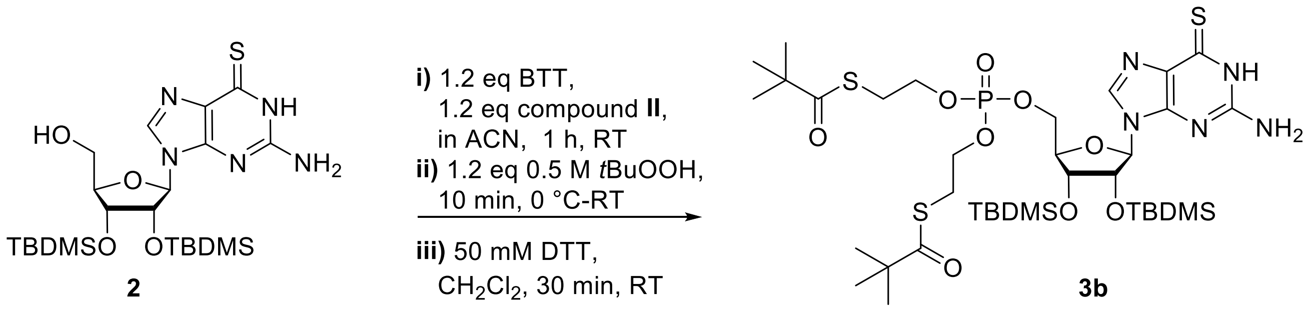

Compound **2** (53 mg, 0.10 mmol) was coevaporated three times with acetonitrile and was further dried under high vacuum for 1 h. It was then suspended in acetonitrile containing 0.3 M 5-(benzylthio)-1*H*-tetrazole (1.2 eq, 0.12 mmol, 0.40 ml). Compound **II**[1] (1.2 eq, 0.12 mmol, 55 mg) in acetonitrile (1.0 ml) was added dropwise and resulting solution was stirred for 45 min at ambient temperature. It was then cooled to 0 °C and a solution of *tert*-butyl hydroperoxide (in decane, 1.2 eq, 0.12 mmol, 0.24 ml 0.5 M in toluene) was introduced slowly. The reaction mixture was stirred for 5 min at 0 °C and then another 5 min at ambient temperature. Solvent was removed under reduced pressure and the crude product was dissolved in dichloromethane containing 50 mM DL-dithiothreitol (2.0 ml). After 30 min solvent was evaporated and the product was purified by column chromatography (10-70% ethyl acetate in c-hexane). Yield: 67 mg as a white foam (74%). TLC (5% MeOH in CH_2Cl_2): 0.38. HR-ESI-MS (m/z): $[M+H]^+$ calculated for $[C_{36}H_{67}N_5O_9PS_3Si_2]^+$: 896.3372; found: 896.3356. ^{1}H-NMR ($CDCl_3$, 400 MHz): δ -0.22, -0.04, 0.115, 0.124 (12 H, 4x s, 4x CH_3 (TBDMS)); 0.81, 0.93 (18 H, 2x s, 2x *t*-Bu (TBDMS)); 1.19, 1.23 (18 H, 2x s, 2x *t*-Bu (SATE)); 3.09 (2 H, t, $^3J_{HH}$ = 6.69 Hz, SCH_2 (SATE)); 3.16 (2 H, t, $^3J_{HH}$ = 6.73 Hz, SCH_2 (SATE)); 4.10 (2 H, dd, $^3J_{HP}$ = 14.51 Hz, $^3J_{HH}$ = 6.73 Hz, $POCH_a$ (SATE)); 4.16 (2 H, dd, $^3J_{HP}$ = 14.58 Hz, $^3J_{HH}$ = 6.91 Hz, $POCH_b$ (SATE)); 4.21-4.26 (2 H, m, CH (4′), CH (3′)); 4.37 (1 H, m, CH_a (5′)), 4.45 (1 H, m, CH_b (5′)), 4.82 (1 H, dd, $^3J_{HH}$ = 5.26, Hz, $^3J_{HH}$ = 4.83 Hz, CH (2′)); 5.71 (1 H, d, $^3J_{HH}$ = 5.62 Hz, CH (1′)); 6.27 (2 H, s br., NH_2); 7.99 (1 H, s, CH (8)) ppm. ^{13}C-NMR ($CDCl_3$, 101 MHz): δ -4.81, -4.55, -4.42, -4.24 4x CH_3 (TBDMS); 18.02, 18.18 2x C_q *t*-Bu (TBDMS); 25.87, 25.94 2x *t*-Bu (TBDMS); 27.44 2x *t*-Bu (SATE); 28.53 (d, $^3J_{CP}$ = 7.27 Hz, SCH_2 (SATE)); 28.60 (d, $^3J_{CP}$ = 7.33 Hz, SCH_2 (SATE)); 46.66, 46.68 2x C_q *t*-Bu (SATE); 66.70 (d, $^2J_{CP}$ = 5.91 Hz, $POCH_2$ (SATE)); 66.76 (d, $^2J_{CP}$ = 5.81 Hz, $POCH_2$ (SATE)); 67.12 (d, $^2J_{CP}$ = 5.81 Hz, C (5′)); 72.31 C (3′), 73.78 C (2′); 83.44 (d, $^3J_{CP}$ = 8.72 Hz, C (4′)); 89.82 C (1′); 130.12 C (5); 140.80 C (8); 147.60 C (4); 152.76 C (2); 175.36 C (6); 205.79, 205.92 2x C=O (SATE) ppm. ^{31}P-NMR ($CDCl_3$, 162 MHz): δ -1.97 ppm

^{1}H-NMR (400 MHz, $CDCl_3$) of compound **3b**

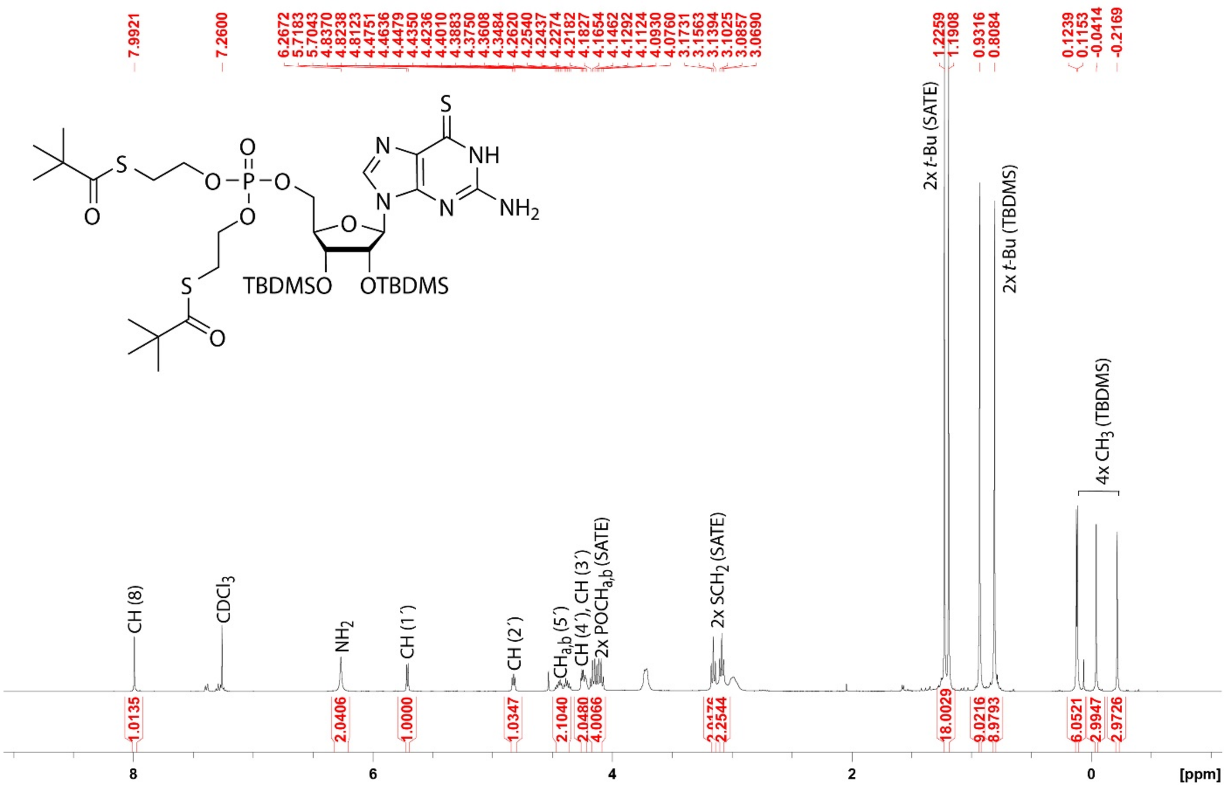

^{13}C-NMR (101 MHz, $CDCl_3$) of compound **3b**

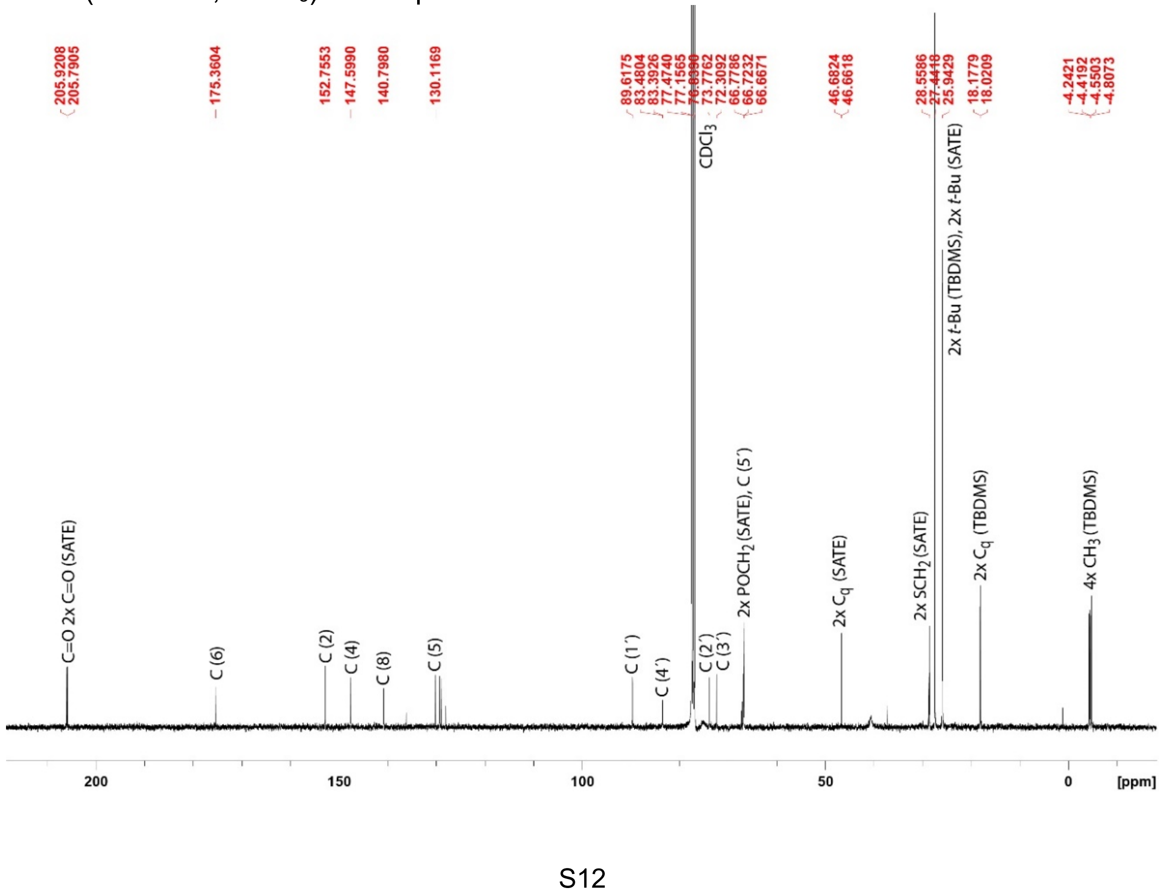

^{31}P-NMR (162 MHz, $CDCl_3$) of compound **3b**

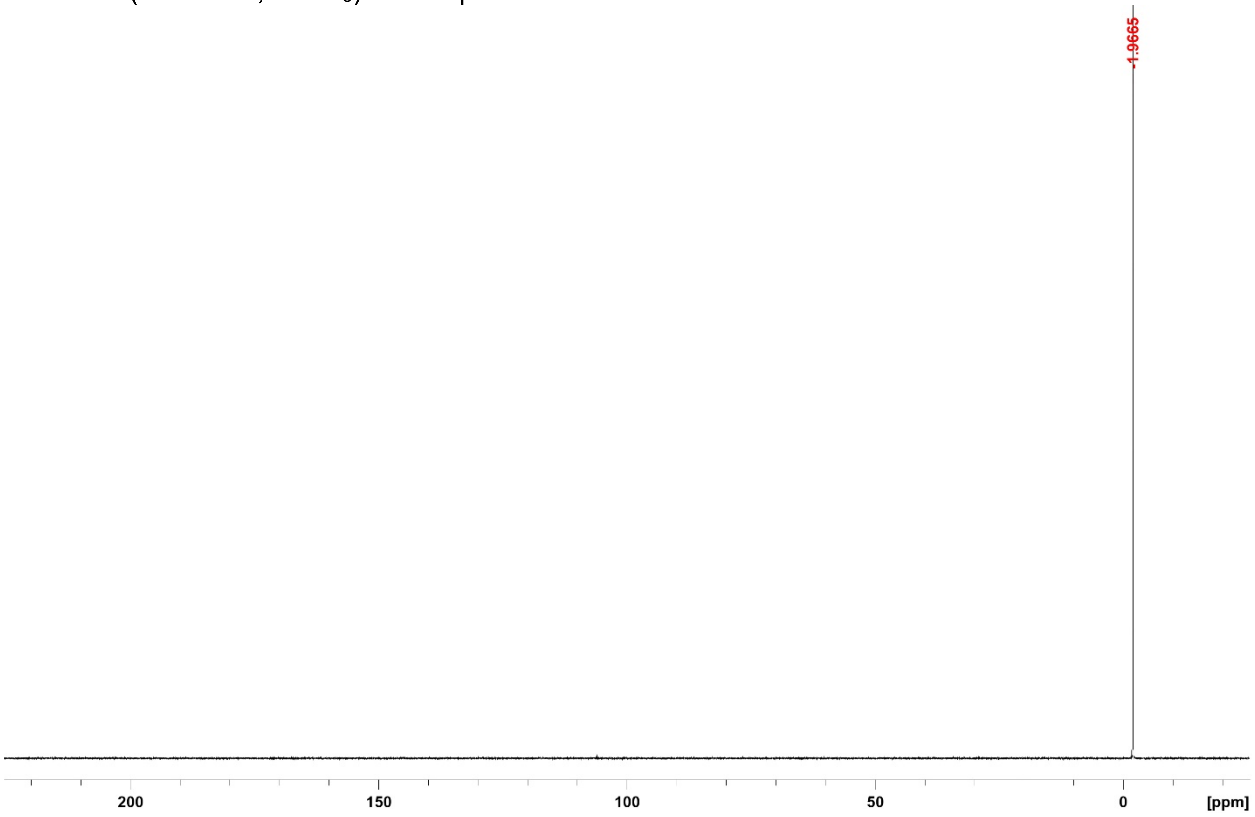

2.5. Synthesis of (4-acetyloxybenzyl)-5′-*O*-(6-thioguanosinyl) phosphate triethylammonium salt (AB-6sGMP, 4a)[5]

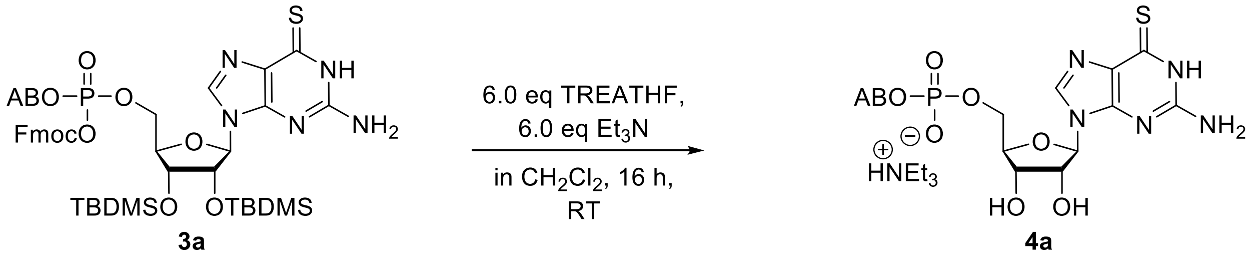

Compound **3a** (31 mg, 0.033 mmol) was dissolved in dichloromethane (1.0 ml) and was consecutively treated with triethylamine trihydrofluoride (6.0 eq, 0.20 mmol, 32 µl) and triethylamine (6.0 eq, 0.20 mmol, 28 µl). The reaction mixture was allowed to stir at ambient temperature for 16 h and was then evaporated to dryness. The oily residue was coevaporated with toluene and was further under high vacuum in order to remove excessive triethylamine. The residue was then dissolved in a minimal amount of dichloromethane and diethyl ether was added. The appearing precipitate was collected by centrifugation and was washed several times with diethyl ether. Yield: 20 mg as white powder (98%). HR-ESI-MS (m/z): $[M]^-$ calculated for $[C_{19}H_{21}N_4O_9PS]^-$: 526.0792; found: 526.0794. ^{1}H-NMR (d_6-DMSO, 600 MHz): δ 1.14 (9 H, t, $^3J_{HH}$ = 7.19 Hz, 3x CH_3 (Et_3N)); 2.26 (3 H, s, CH_3 (AB)); 2.96 (6 H, q, $^3J_{HH}$ = 6.45 Hz, 3x CH_2 (Et_3N)); 3.86 (1 H, m , CH_a (5′)); 3.92 (1 H, m , CH_b (5′)); 4.00 (1 H, dd, $^3J_{HH}$ = 7.37 Hz, $^3J_{HH}$ = 3.85 Hz, CH (4′)); 4.17 (1 H, m, CH (3′)); 4.53 (1 H, m, CH (2′)); 4.72 (2 H, m br., CH_2 (AB)); 5.40 (1 H, s br., OH (3′)); 5.49 (1 H , s br., OH (2′)); 5.70 (1 H, d, $^3J_{HH}$ = 5.72 Hz, CH (1′)); 6.98 (2 H, s br., NH_2); 7.06 (2 H, d, $^3J_{HH}$ =8.42 Hz, aromat. CH (AB)); 7.35 (2 H, d, $^3J_{HH}$ =8.38 Hz, aromat. CH (AB)); 8.16 (1 H, c, CH (8) ppm. ^{13}C-NMR (d_6-DMSO, 151 MHz): δ 8.69 3x CH_3 Et_3N; 20.85 CH_3 (AB); 45.39 3x CH_2 Et_3N; 64.68 (d, $^2J_{CP}$ = 5.43 Hz, C (5′)); 65.45 (d, $^2J_{CP}$ = 5.35 Hz, CH_2 (AB)); 70.85 C (3′); 73.51 C (2); 83.94 (d, $^3J_{CP}$ = 7.65 Hz, C (4′)); 86.83 C (1′); 121.44, 128.16 aromat. C (AB); 128.38 C (5); 136.82 (d, $^3J_{CP}$ = 7.57 Hz, C_q (AB)); 138.79 C (8); 147.99 C (4), 149.52 C_q (AB); 153.11 C (2); 169.22 C=O (AB); 175.06 C (6) ppm. ^{31}P-NMR (d_6-DMSO, 162 MHz): δ -1.00 ppm

^{1}H-NMR (600 MHz, d_6-DMSO) of compound **4a**

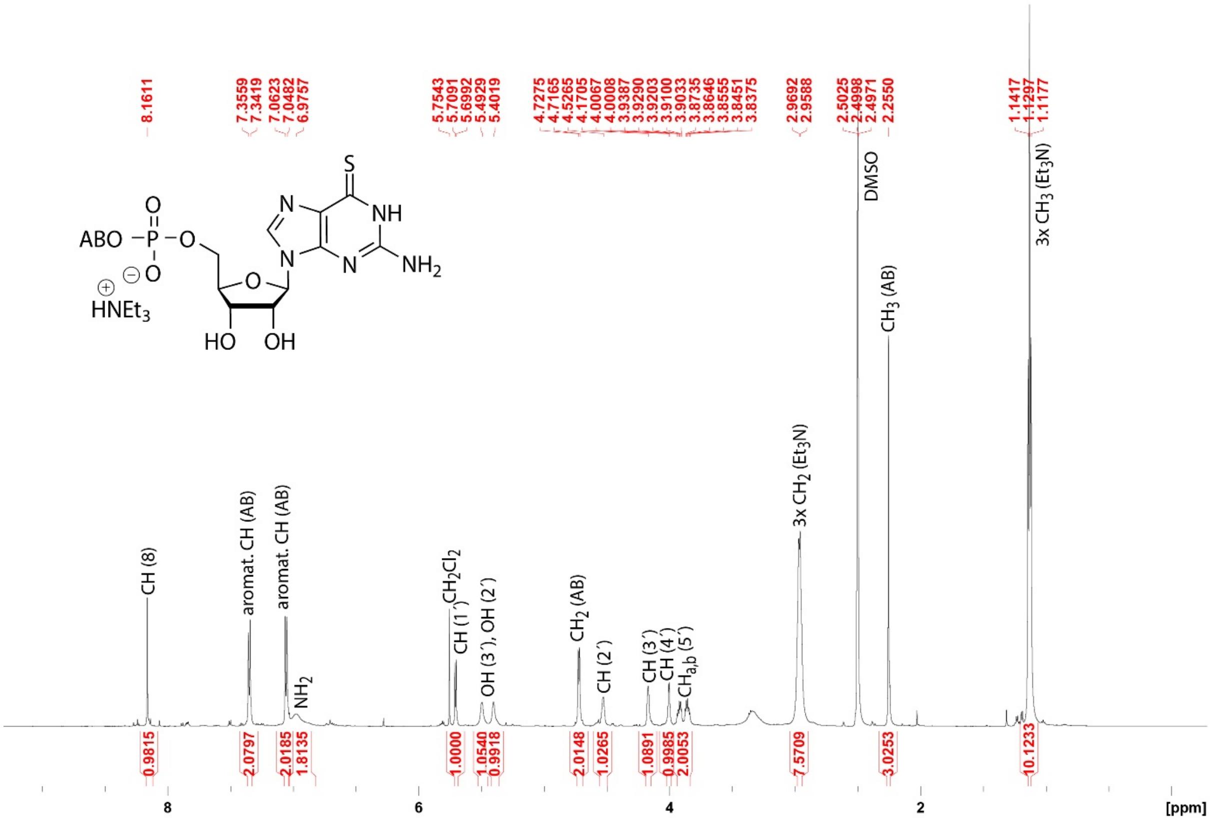

^{13}C-NMR (151 MHz, d_6-DMSO) of compound **4a**

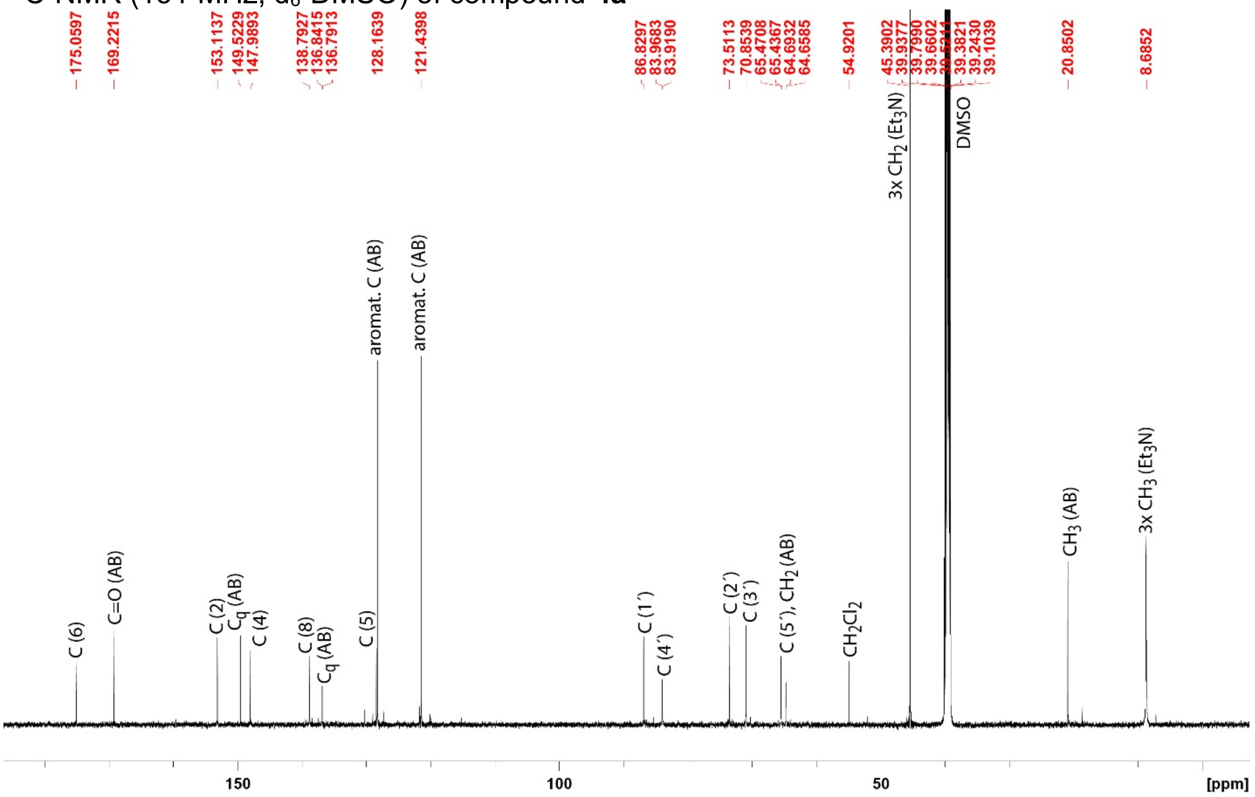

^{31}P-NMR (162 MHz, d_6-DMSO) of compound **4a**

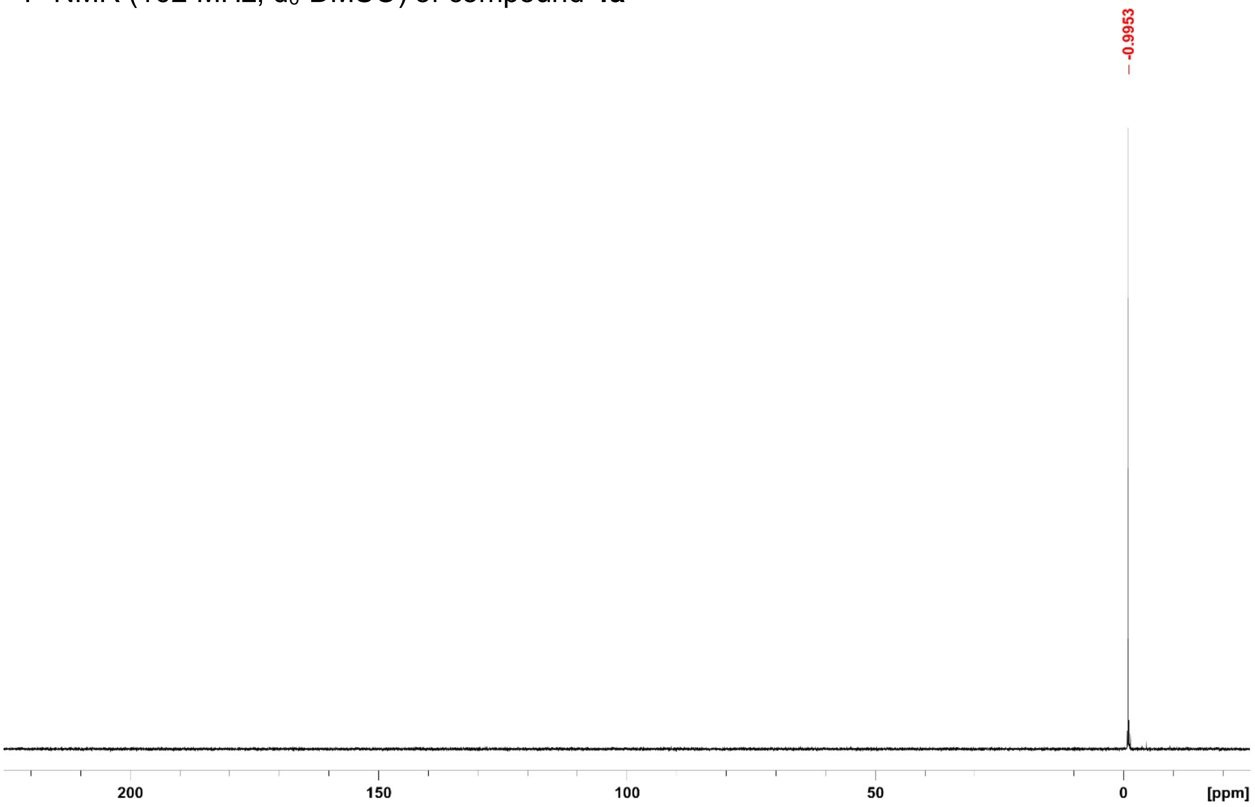

2.6 Synthesis of bis(*S*-pivaloyl-2-thioethyl)-5′-*O*-(6-thioguanosinyl) phosphate ((*t*BuSATE)$_2$-6sGMP, 4b)[5]

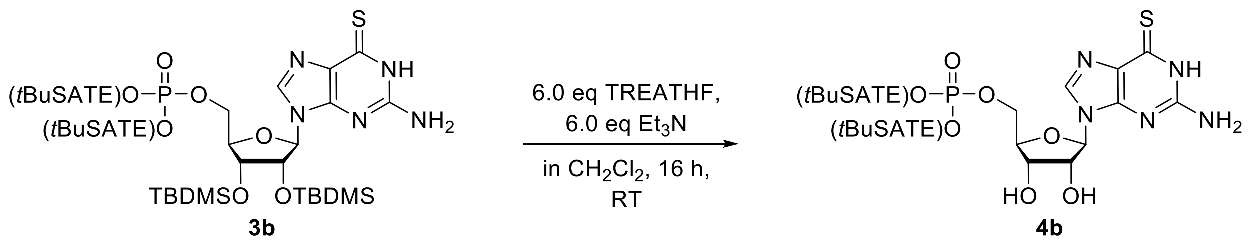

Triethylamine trihydrofluoride (6.0 eq, 0.32 mmol, 52 µl) and triethylamine (6.0 eq, 0.32 mmol, 45 µl) were added to a solution of compound **3b** (48 mg, 0.053 mmol) in dichloromethane (1.5 ml). The reaction mixture was stirred for 16 h at ambient temperature followed by evaporation of the solvent and purification by column chromatography (2-8% MeOH in CH_2Cl_2). Yield: 24 mg as colorless solid (70%). TLC (10% MeOH in CH_2Cl_2): 0.44. HR-ESI-MS (m/z): $[M+H]^+$ calculated for $[C_{24}H_{39}N_5O_9PS_3]^+$: 668.1642; found: 668.1612. ^{1}H-NMR (d$_6$-DMSO, 600 MHz): δ 1.16, 1.17 (18 H, 2x s, 2x *t*-Bu (SATE)); 3.08 (4 H, m, 2x SCH_2 (SATE)); 3.98-4.05 (5 H, 2x $POCH_2$ (SATE), CH (4′)); 4.13-4.18 (2 H, m, CH (3′), CH_a (5′)); 4.22 (1 H, m, CH_b (5′)), 4.44 (1 H, dd, $^3J_{HH}$ = 10.57 Hz, $^3J_{HH}$ = 5.25 Hz, CH (2′)); 5.25 (1 H, d, $^3J_{HH}$ = 4.90 Hz, OH (2′)); 5.58 (1 H, d, $^3J_{HH}$ = 5.66 Hz, OH (3′)); 5.72 (1 H, d, $^3J_{HH}$ = 5.46 Hz, CH (1′)); 6.82 (2 H, s br., NH_2); 8.04 (1 H, s, CH (8)); 11.97 (1 H, s br, NH) ppm. ^{13}C-NMR (d$_6$-DMSO, 151 MHz): δ 26.87 2x *t*-Bu (SATE); 28.06 (d, $^3J_{CP}$ = 7.13 Hz, SCH_2 (SATE)); 28.10 (d, $^3J_{CP}$ = 7.19 Hz, SCH_2 (SATE)); 46.00 2x C_q *t*-Bu (SATE); 65.61 (d br., $^2J_{CP}$ = 5.08 Hz, 2x $POCH_2$); 67.29 (d, $^2J_{CP}$ = 5.77 Hz, C (5′)); 69.96 C (3′), 72.99 C (2′); 82.28 (d, $^3J_{CP}$ = 7.50 Hz, C (4′)); 86.75 C (1′); 128.44 C (5); 138.32 C (8); 147.86 C (4); 153.07 C (2); 175.23 C (6); 205.06 2x C=O (SATE) ppm. ^{31}P-NMR (d$_6$-DMSO, 162 MHz): δ -1.82 ppm

^{1}H-NMR (600 MHz, d_6-DMSO) of compound **4b**

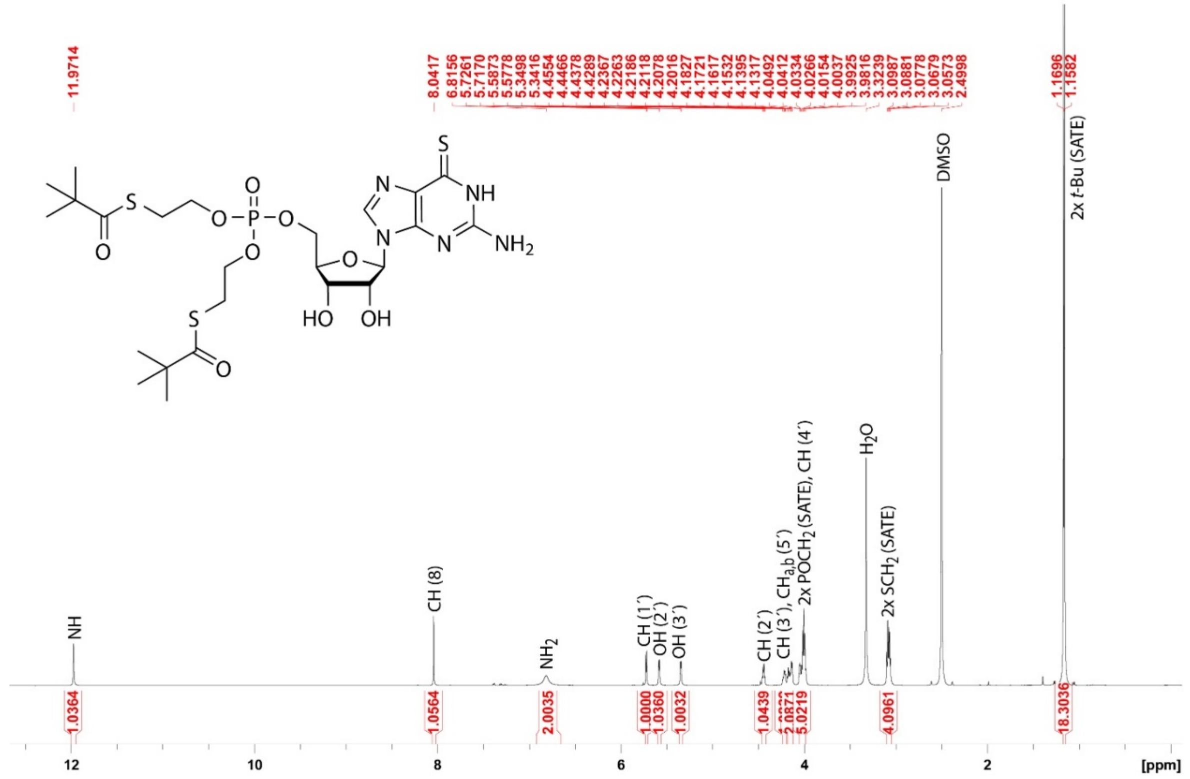

^{13}C-NMR (151 MHz, d_6-DMSO) of compound **4b**

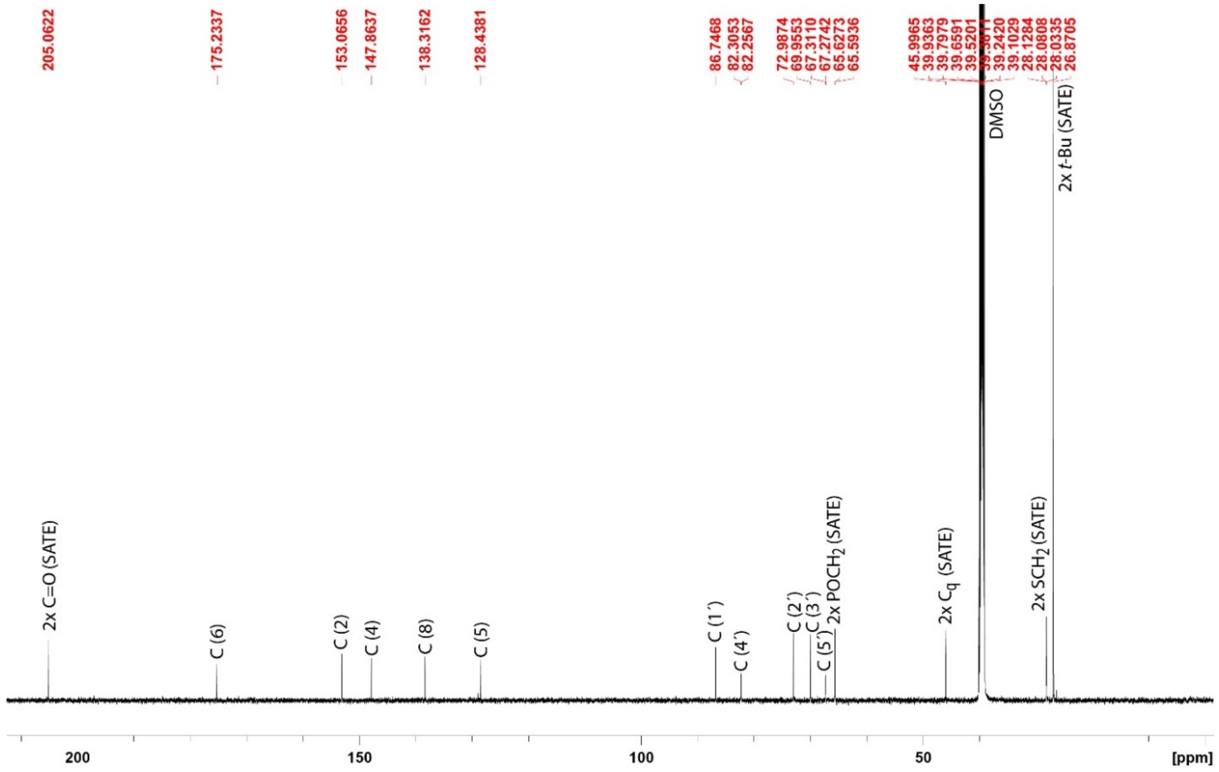

^{31}P-NMR (162 MHz, d_6-DMSO) of compound **4b**

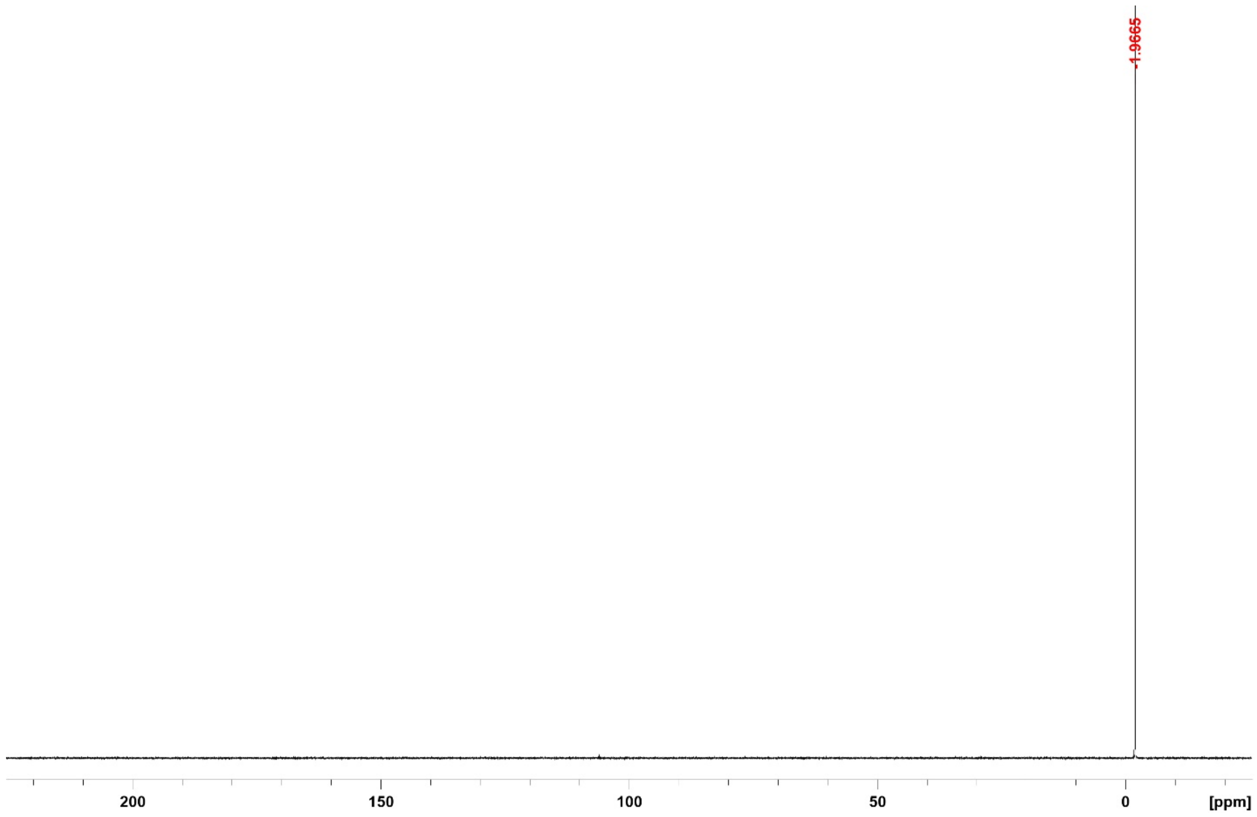

3. Synthesis of bis(4-acetyloxybenzyl)-5′-*O*-(S^6-(4-acetyloxybenzyl) guanosinyl) phosphate (5)

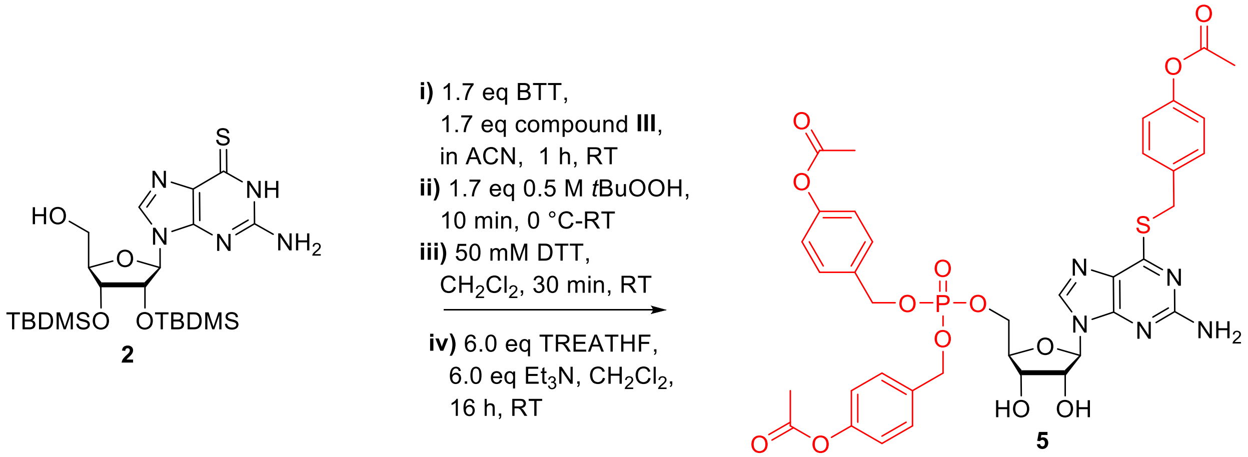

Compound **2** (179 mg, 0.34 mmol) was dried under high vacuum overnight. It was then suspended in acetonitrile containing 0.3 M 5-(benzylthio)-1*H*-tetrazole (1.7 eq, 0.58 mmol, 1.92 ml). Compound **III** (1.7 eq, 0.58 mmol, 266 mg) in acetonitrile (1.0 ml) was added dropwise and resulting solution was stirred for 1 h at ambient temperature. It was then cooled to 0 °C and a solution of *tert*-butyl hydroperoxide (in decane, 1.7 eq, 0.58 mmol, 1.20 ml 0.5 M in toluene) was added dropwise. The reaction mixture was stirred for 5 min at 0 °C and then another 5 min at ambient temperature. Solvent was removed under reduced pressure and the crude product was dissolved in dichloromethane containing 50 mM DL-dithiothreitol (2.0 ml). After 30 min solvent was evaporated and the product was purified by flash chromatography (0-4% MeOH in CH_2Cl_2). The colorless oil was then dissolved in dichloromethane (8.0 ml) and triethylamine trihydrofluoride (6.0 eq, 2.03 mmol, 0.33 ml) and triethylamine (6.0 eq, 2.03 mmol, 0.28 ml) were added.[5] The reaction mixture was stirred for 16 h at ambient temperature and was then extracted trice with 5% sodium bicarbonate solution. Drying over sodium sulfate was followed by concentration and flash chromatography (2-7% MeOH in CH_2Cl_2). Yield: 40 mg as a colorless oil (14%). TLC (7% MeOH in CH_2Cl_2): 0.44. HR-ESI-MS (m/z): $[M+H]^+$ calculated for $[C_{37}H_{39}N_5O_{13}PS]^+$: 824.1997; found: 824.1958. ^{1}H-NMR ($CDCl_3$, 700 MHz): δ 2.28, 2.29 (3x 3H, 2x s, 3x CH_3 (AB)); 3.53 (1 H, s br., OH); 4.05-4.08 (2 H, m, CH_a (5′), CH (3′)); 4.17-4.21 (2 H, m, CH_b (5′), CH (4′)); 4.47 (2 H, m, CH (2′), OH); 4.52 (2 H, d, $^4J_{HH}$ = 3.40 Hz, CH_2 (AB, S^6)); 4.92-5.04 (4 H, m, 2x CH_2 (AB)); 5.15 (2 H, s br., NH_2); 5.75 (1 H, d, $^3J_{HH}$ = 5.38 Hz, CH (1′)); 7.00-7.06, 7.30-7.44 (12 H, m, aromat. CH

(AB)); 7.71 (1 H, s, CH (8)) ppm. ^{13}C-NMR ($CDCl_3$, 101 MHz): δ 21.26 3x CH_3 (AB)); 32.05 CH_2 (AB, *S*[6]); 66.75 (d, $^2J_{CP}$ = 5.96 Hz, C (5′)); 69.12 (d, $^2J_{CP}$ = 5.25 Hz, $CH_{2,a}$ (AB)); 69.23 (d, $^2J_{CP}$ = 5.29 Hz, $CH_{2,b}$ (AB)); 71.19 C (3′), 73.96 C (2′); 83.27 (d, $^3J_{CP}$ = 8.26 Hz, C (4′)); 89.42 C (1′); 121.71, 122.06, 122.11 aromat. C (AB); 125.86 C (5); 129.59, 129.72, 130.29 aromat. C (AB); 133.34-133.36 (d br., 2x C_q (AB)); 135.60 aromat. C (AB); 138.96 C (8); 149.88 aromat. C (AB); 150.20 C (4), 150.93, 151.00 aromat. C (AB); 158.87 C (2); 161.24 C (6); 169.63, 169.89 3x C=O (AB) ppm. ^{31}P-NMR ($CDCl_3$, 162 MHz): δ -1.02 ppm.

^{1}H-NMR (700 MHz, $CDCl_3$) of compound **5**

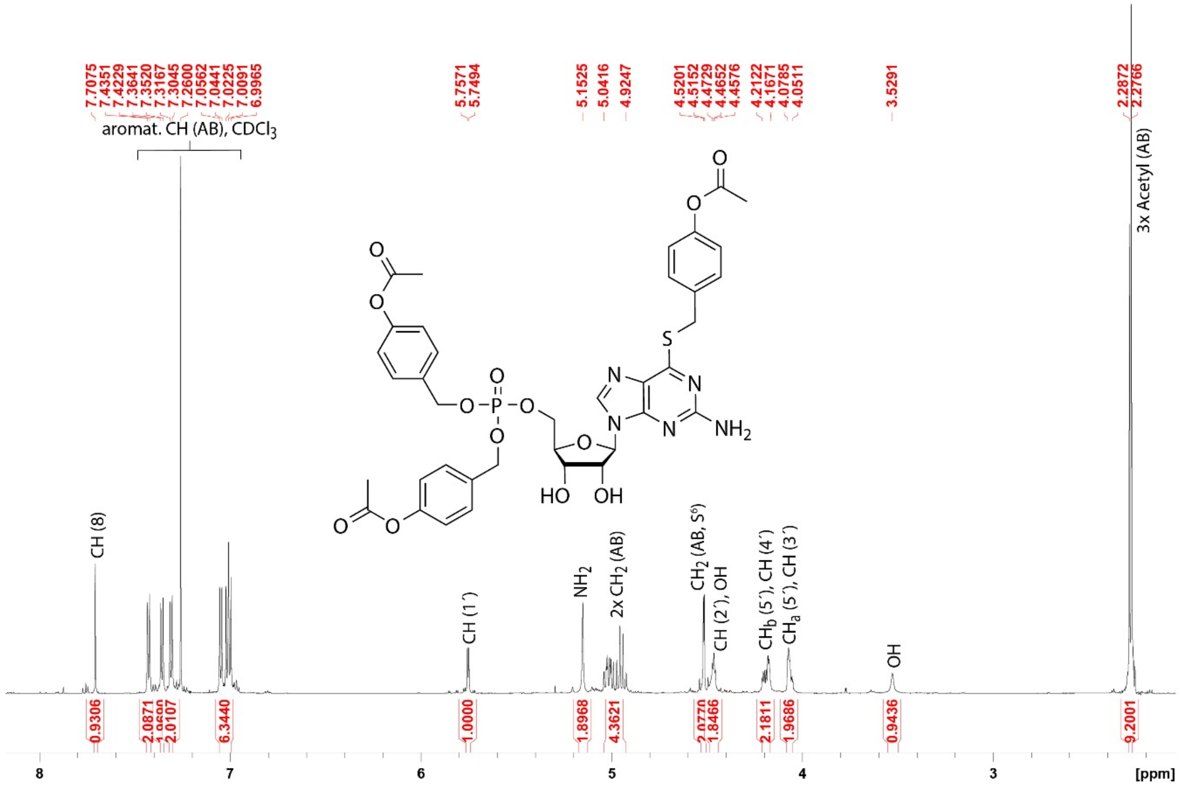

^{13}C-NMR (176 MHz, $CDCl_3$) of compound **5**

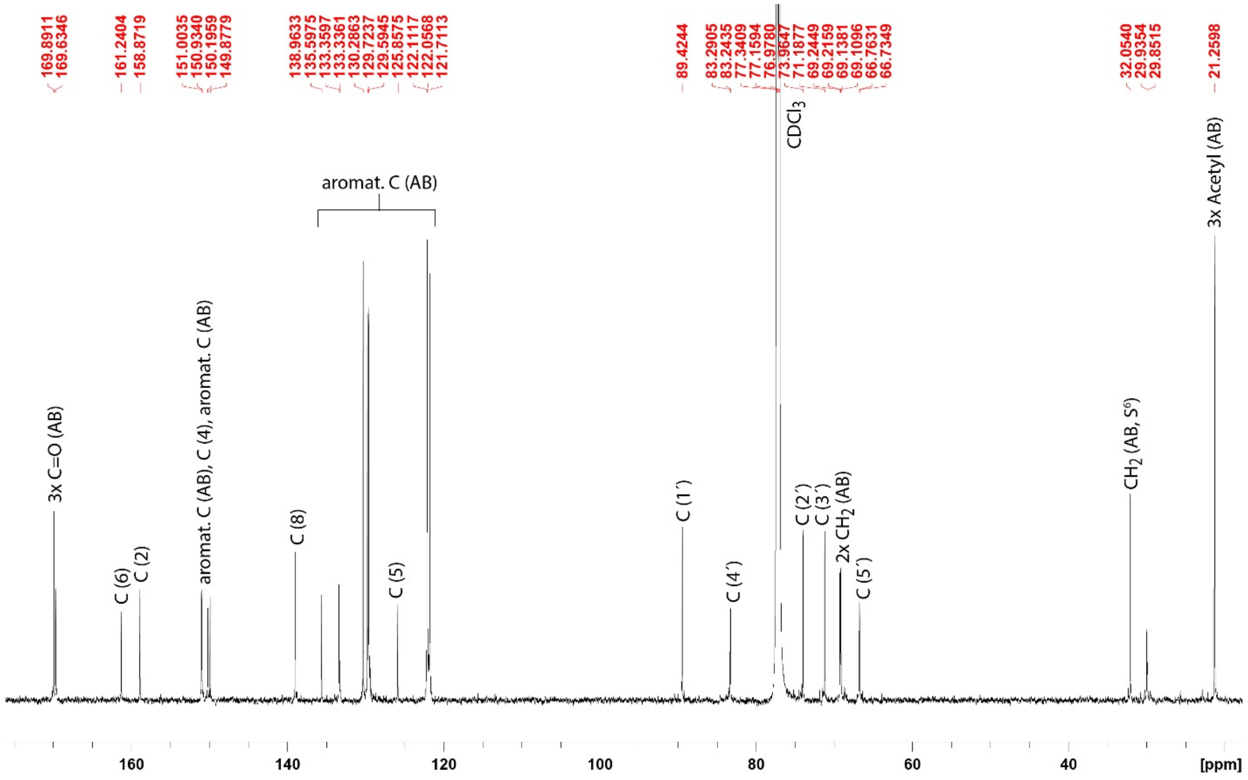

^{31}P-NMR (162 MHz, CDCl$_3$) of compound **5**

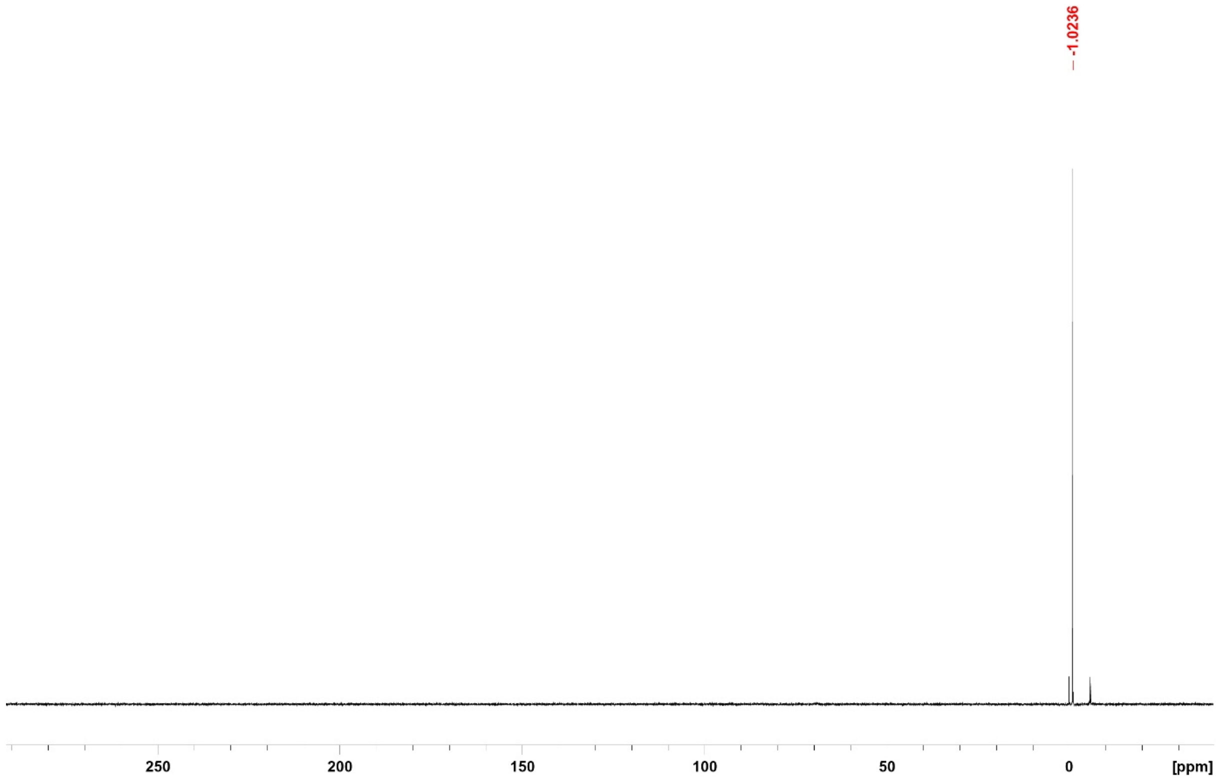

HMBC (700 MHz, CDCl$_3$) of compound **5**

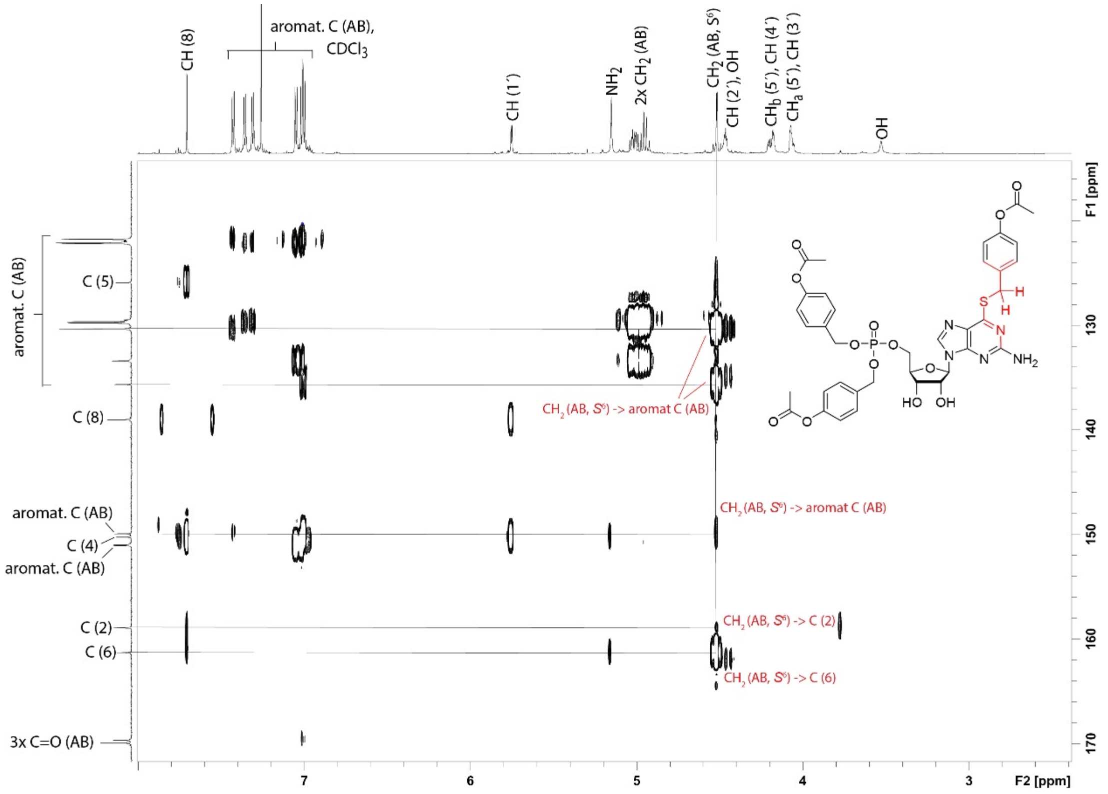

4. Synthesis of *cyclo*Sal-6sGMP, Cl*cyclo*Sal-6sGMP and ProTide-6sGMP (10a-c)

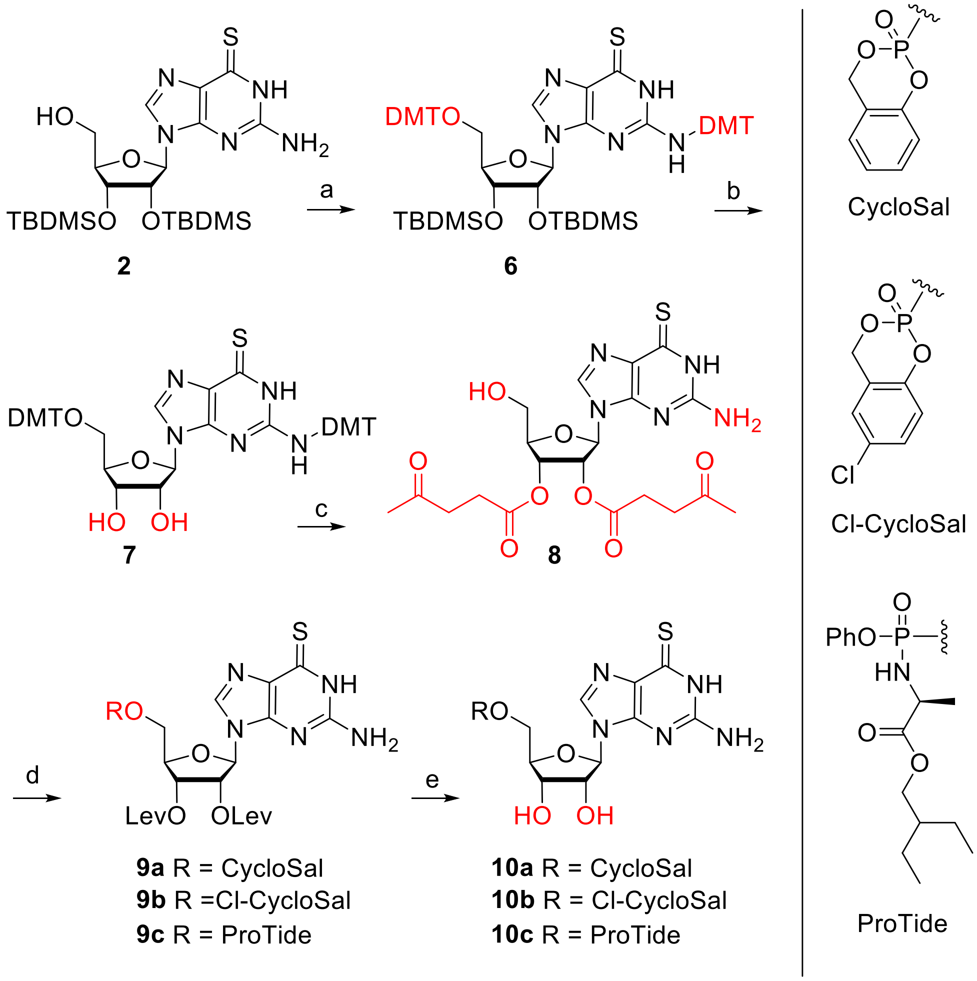

Scheme 2 Synthesis of *cyclo*Sal-6sG **10a,** Cl*cyclo*Sal-6sG **10b** and ProTide-6sG **10c**. **a)** 2.5 eq DMTCl, pyridine, 16 h, RT, 76%. **b)** 5.0 eq Et_3N, 10.0 eq TREATHF, CH_2Cl_2, 24 h, RT, 69%. **c)** i) 4.3 eq DCC, 8.7 eq LevOH, 0.15 eq DMAP, CH_2Cl_2, 1 h, 0 °C-RT; ii) 4.0 eq benzenesulfonic acid, CH_2Cl_2/MeOH, 30 min, 0°C, 36%. **d)** for **9a**: i) 1.2 eq compound **IV**, 1.2 eq BTT, ACN, 1 h, RT-0°C; ii) 1.2 eq *t*BuOOH; iii) 50 mM DTT, CH_2Cl_2, 30 min, RT, 68%. for **9b**: i) 1.2 eq compound **V**, 1.2 eq BTT, ACN, 1 h, RT-0°C; ii) 1.2 eq *t*BuOOH; iii) 50 mM DTT, CH_2Cl_2, 30 min, RT, 35%. for **9c**: i) 1.0 eq compound **VI**, 1.0 eq $C_6H_5OP(O)Cl_2$, 1.0 eq Et_3N, CH_2Cl_2, 16 h, -78 °C-RT; ii) 8.0 eq NMI, THF/pyridine, 24 h, 0 °C-RT, 45%. **e)** for **10a,b**: H_2NNH_2, H_2O/pyridine/HOAc, 5 min, RT-0 °C, 49% for **10a**, 51% for **10b**; for **10c**: 2 M H_2NNH_2, H_2O/pyridine/HOAc, 15 min, RT-0 °C, 61%. Overall yield: **10a:** 6%, **10b**: 5%, **10c**: 5%.

4.1. Synthesis of 2′,3′-di-*O*-(*tert*-butyldimethylsilyl)-*N*^{2},5′-*O*-di-(4,4′-dimethoxytrityl)-6-thio guanosine (6)

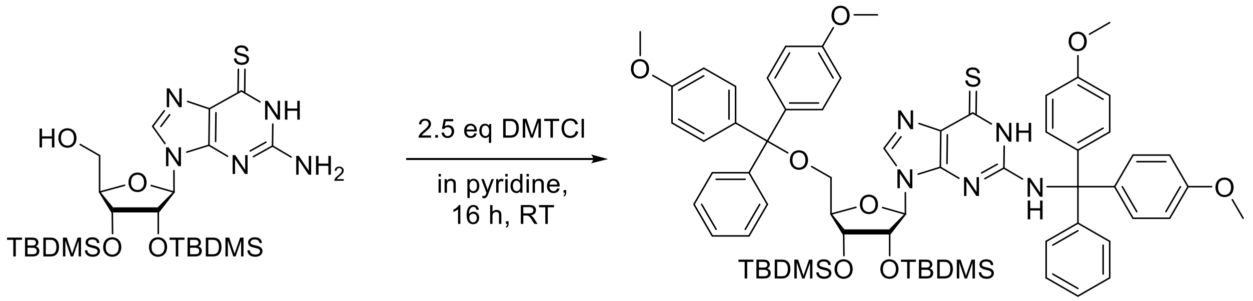

2 **6**

Compound **2** (413 mg, 0.78 mmol) was dried by azeotropic distillation of pyridine. It was then dissolved in pyridine (5.0 ml) and 4,4′-dimethoxytrityl chloride (2.5 eq, 1.96 mmol, 663 mg) was added in two portions over a period of 30 min. The reaction mixture was stirred for 16 h at ambient temperature, was then diluted with ethyl acetate and washed with 5% sodium bicarbonate solution. The organic layer was dried over sodium sulfate and solvent was evaporated. The oily residue was coevaporated with toluene and subjected to column chromatography (10-30% ethyl acetate in c-hexane). Yield: 677 mg as off-white foam (76%). TLC (3% MeOH in CH_2Cl_2): 0.47. HR-ESI-MS (m/z): $[M+H]^+$ calculated for $[C_{64}H_{78}N_5O_8SSi_2]^+$: 1132.5104; found: 1132.5076. ^{1}H-NMR ($CDCl_3$, 400 MHz): δ -0.18, -0.10, 0.0069, 0.032 (4x 3 H, 4x s, 4x CH_3 (TBDMS)); 0.83, 0.86 (2x 9 H, 2x s, 2x *t*-Bu (TBDMS)); 3.10 (1 H, dd, $^2J_{HH}$ = 10.74 Hz, $^3J_{HH}$ = 2.94 Hz, CH_a (5′)); 3.54 (1 H, dd, $^2J_{HH}$ = 10.95 Hz, $^3J_{HH}$ = 1.75 Hz, CH_b (5′)); 3.73, 3.78 (4x 3 H, 2x s, 4x OCH_3 (DMT)); 4.08 (1 H, m, CH (4′)); 4.16 (1 H, dd, $^3J_{HH}$ = 4.45 Hz, $^3J_{HH}$ = 1.30 Hz, CH (3′)); 4.85 (1 H, dd, $^3J_{HH}$ = 6.66 Hz, $^3J_{HH}$ = 4.66 Hz, CH (2′)); 5.70 (1 H, d, $^3J_{HH}$ = 7.05 Hz, CH (1′)); 6.15 (1 H, s br., NH); 6.73-7.58 (26 H, m, aromat. CH (DMT)); 8.80 (1 H, s, CH (8) ppm. ^{13}C-NMR ($CDCl_3$, 101 MHz): δ -4.94, -4.64, -4.42, -4.23 4x CH_3 (TBDMS)); 17.93, 18.01 2x C_q (TBDMS); 25.83, 25.86 2x *t*-Bu (TBDMS); 55.21, 55.22, 55.30 4x OCH_3 (DMT); 63.24 C (5′); 70.45 aromat. C (DMT); 72.46 C (3′); 74.56 C (2); 85.51 C (4′); 86.63 aromat. C (DMT); 87.56 C (1′); 113.24, 113.36, 113.39, 114.13, 127.10, 127.85, 127.91, 128.09, 128.78, 129.21, 129.49, 129.53, 129.85, 129.95 aromat. C (DMT); 130.62 C (5); 134.71, 134.76, 135.95, 136.23 aromat. C (DMT); 139.65 C (8); 143.07, 144.77 aromat. C (DMT); 147.04 C (4), 150.74 C (2); 158.59, 158.62, 158.98, 159.01 aromat. C (DMT); 174.72 C (6) ppm.

^{1}H-NMR (400 MHz, $CDCl_3$) of compound **6**

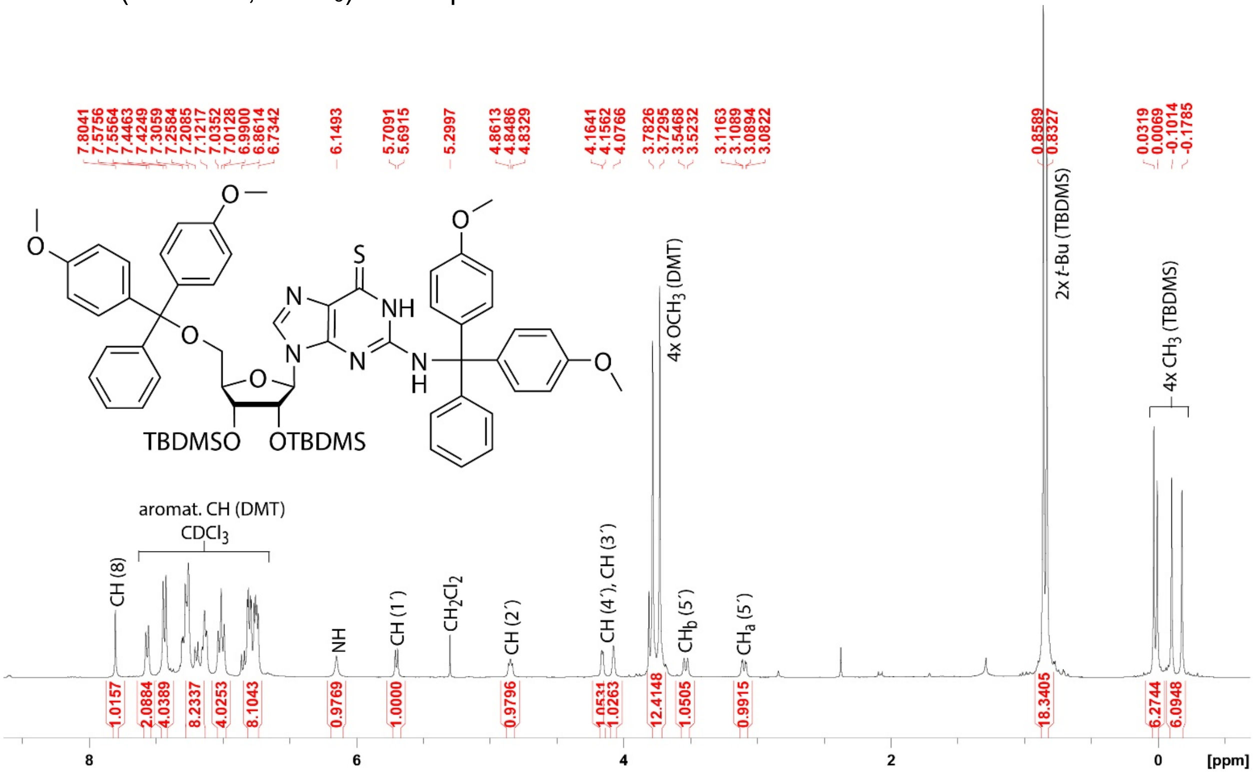

^{13}C-NMR (101 MHz, $CDCl_3$) of compound **6**

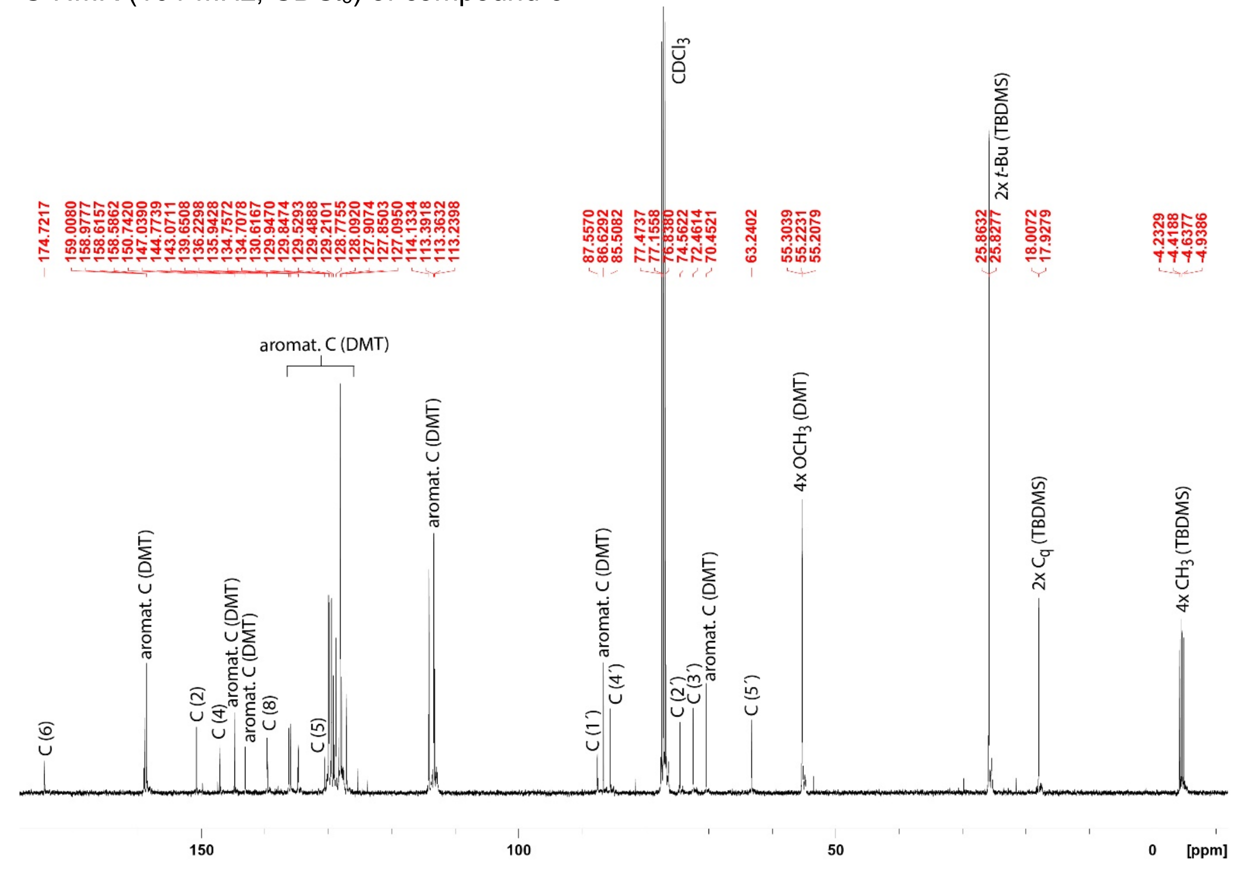

4.2. Synthesis of *N*^{2},5′-*O*-di-(4,4′-dimethoxytrityl)-6-thioguanosine (7)[5]

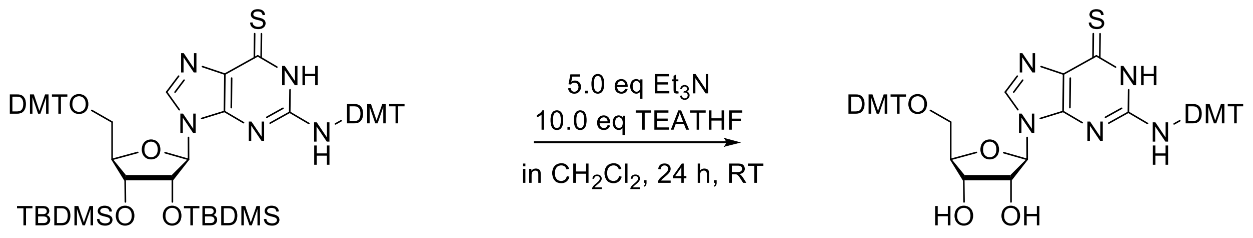

6 **7**

Compound **6** (630 mg = 0.56 mmol) was dissolved in dichloromethane (2.0 ml) and was treated with triethylamine (5.0 eq, 2.80 mmol, 0.39 ml) and triethylamine trihydrofluoride (10.0 eq, 5.58 mmol, 0.91 ml). The reaction mixture was stirred for 24 h at ambient temperature followed by dilution with dichloromethane and extraction with 5% sodium bicarbonate solution. The organic layer was dried over sodium sulfate, was concentrated and was subjected to column chromatography (0-3% MeOH in CH_2Cl_2). Yield: 346 mg as slightly foam (69%). TLC (3% MeOH in CH_2Cl_2): 0.38. HR-ESI-MS (m/z): $[M+H]^+$ calculated for $[C_{52}H_{50}N_5O_8S]^+$: 904.3375; found: 904.3353. ^{1}H-NMR (d_6-DMSO, 400 MHz): δ 3.08 (2 H, d, $^3J_{HH}$ = 4.35 Hz, CH_2 (5′)); 3.67, 3.73 (4x 3 H, 2x s, 4x OCH_3 (DMT)); 3.71 (1 H, m, CH (3′)); 3.85-3.91 (2 H, m, CH (2′), CH (4′)); 4.93 (1 H, d, $^3J_{HH}$ = 6.23 Hz, OH (3′)); 5.09 (1 H, d, $^3J_{HH}$ = 5.12 Hz, OH (2′)); 5.20 (1 H, d, $^3J_{HH}$ = 3.70 Hz, CH (1′)); 6.80-6.86, 7.11-7.37 (26 H, m, aromat. CH (DMT)); 7.90 (1 H, s, CH (8); 7.93, 12.12 (2x 1 H, 2x s br., 2x NH) ppm. ^{13}C-NMR (d_6-DMSO, 151 MHz): 54.96, 55.03 4x OCH_3 (DMT); 63.96 C (5′); 69.64 aromat. C (DMT); 69.84 C (3′); 72.62 C (2); 82.18 C (4′); 85.55 aromat. C (DMT); 87.80 C (1′); 112.99, 113.01, 113.16, 126.39, 126.70, 127.66, 127.69, 127.83, 128.07 aromat. C (DMT); 128.54 C (5); 129.65, 129.68, 129.71, 129.73, 135.41, 135.55, 136.50, 136.78 aromat. C (DMT); 138.99 C (8); 144.58, 144.84 aromat. C (DMT); 145.66 C (4), 150.54 C (2); 157.70, 157.52, 158.08 aromat. C (DMT); 175.35 C (6) ppm.

^{1}H-NMR (400 MHz, d_6-DMSO) of compound **7**

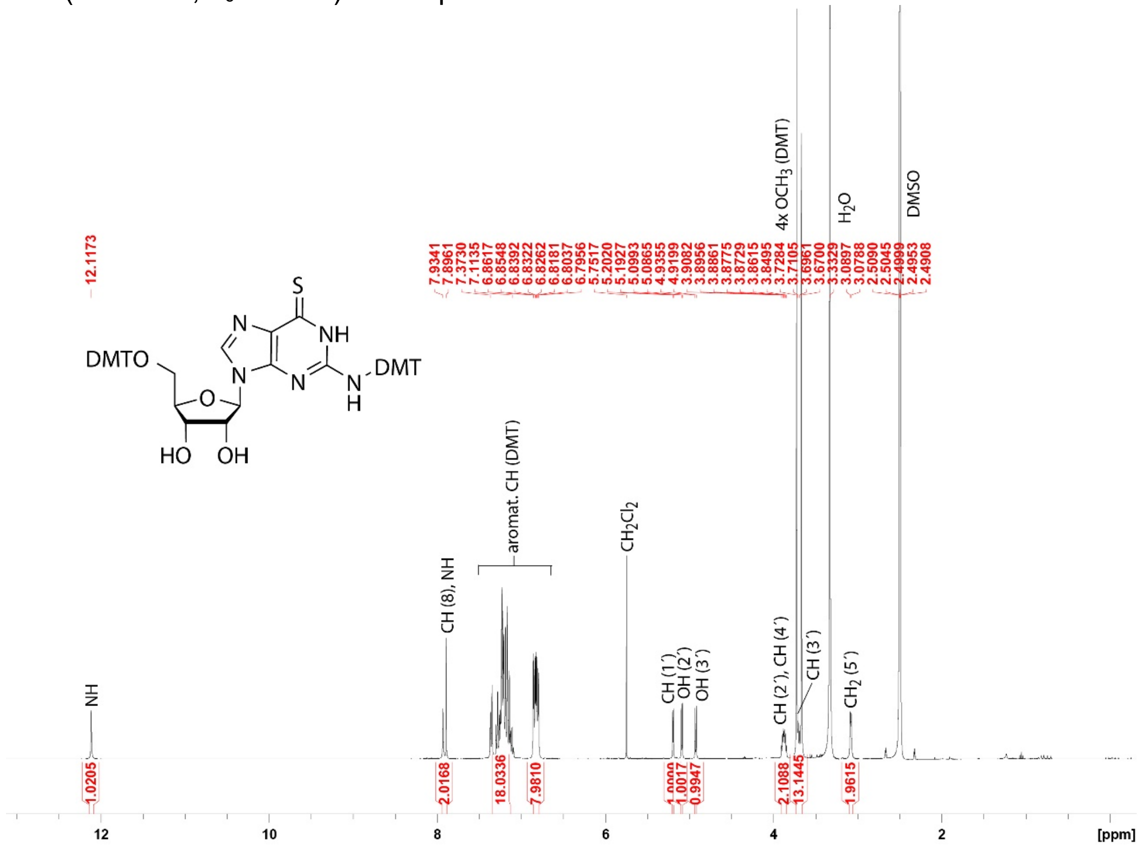

^{13}C-NMR (151 MHz, d_6-DMSO) of compound **7**

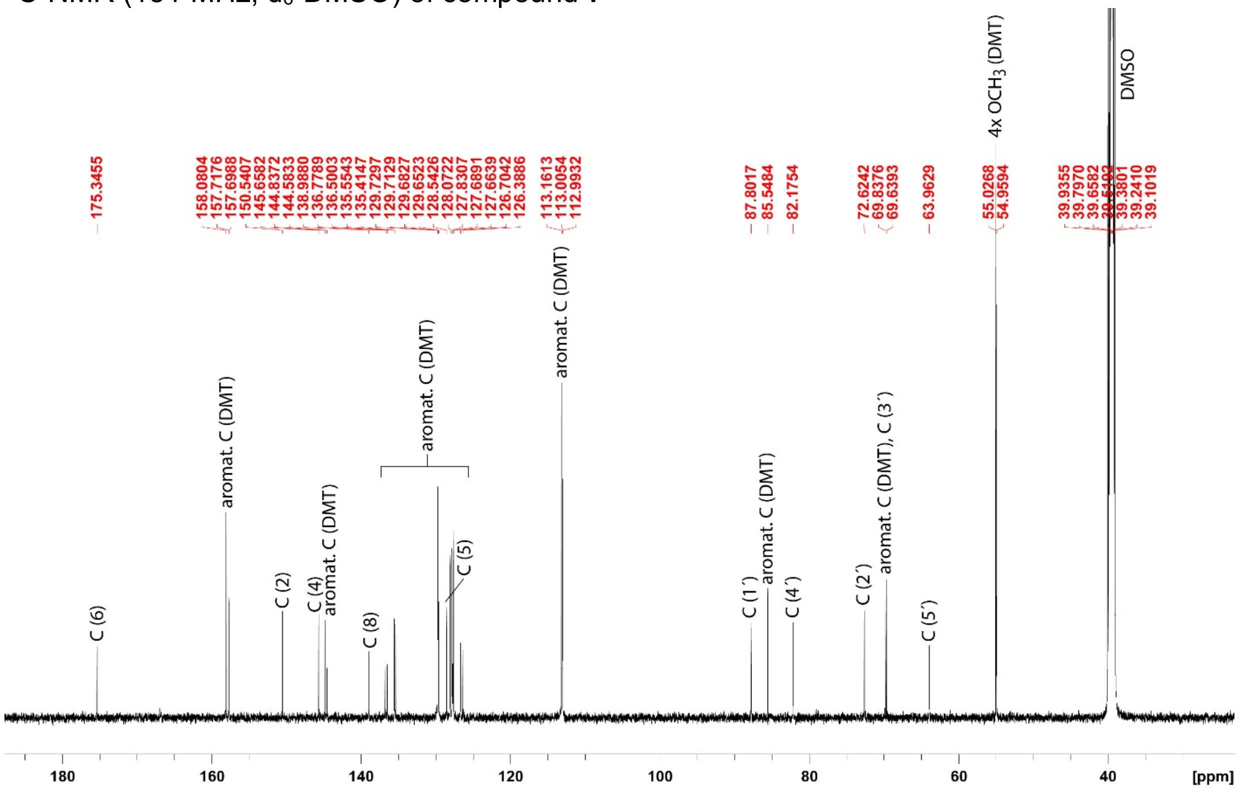

4.3. Synthesis of 2′,3′-di-*O*-levulinoyl-6-thioguanosine (8)[6,7]

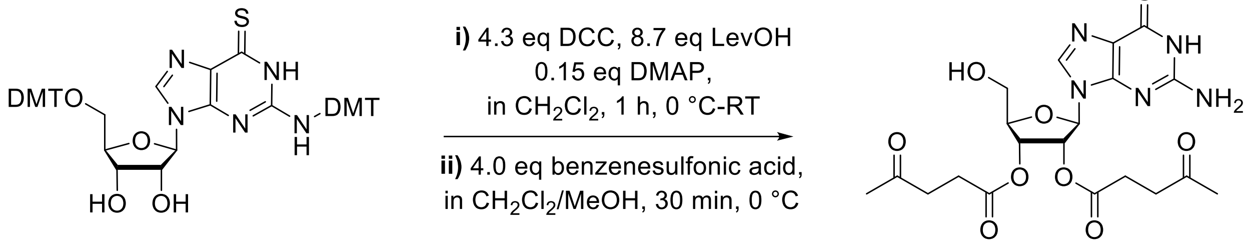

7 8

N,N′-Dicyclohexylcarbodiimid (4.3 eq, 1.64 mmol, 338 mg) was dissolved in dichloromethane (2.6 ml) and cooled to 0 °C. Levulinic acid (8.7 eq, 3.32 mmol, 0.34 ml) was added and the reaction mixture was stirred for 5 min at 0 °C and for 10 min at ambient temperature. A solution of compound **7** (346 mg, 0.38 mmol) and 4-(dimethylamino)pyridine (0.15 eq, 0.057 mmol, 7 mg) in dichloromethane (3.0 ml) was added and stirred for 1 h at ambient temperature. 5% sodium bicarbonate solution was added to the vigorously stirred reaction mixture. Phases were separated and the organic layer was twice with 5% sodium bicarbonate solution. Drying over sodium sulfate was followed by concentration and column chromatography (0-3% MeOH in CH_2Cl_2) yielding the 2′,3′-O-levulinated compound a white foam. The latter was dissolved in dichloromethane/methanol (9/1, 5.0 ml) and was cooled to 0 °C. A solution of benzenesulfonic acid (4.0 eq, 1.44 mmol, 229 mg) in dichloromethane/methanol (9/1, 10.0 ml) was added slowly in four portions over a period of 30 min at 0 °C. 5% Sodium bicarbonate solution was added to the bright orange reaction mixture which was stirred vigorously for 5 min at 0 °C. Phases were separated and the organic layer was with 5% sodium bicarbonate solution and brine. Drying over sodium sulfate was followed by concentration and column chromatography (1-7% MeOH in CH_2Cl_2). Yield: 68 mg as a colorless solid (36%). TLC (5% MeOH in CH_2Cl_2): 0.25. HR-ESI-MS (m/z): $[M+H]^+$ calculated for $[C_{20}H_{26}N_5O_8S]^+$: 496.1497; found: 496.1482. ^{1}H-NMR (d_6-DMSO, 400 MHz): δ 2.04, 2.13 (2x 3 H, 2x s, 2x CH_3 (Lev)); 2.44 (2 H, t, $^3J_{HH}$ = 6.48 Hz, CH_2 (Lev)); 2.58 (2 H, t, $^3J_{HH}$ = 6.24 Hz, CH_2 (Lev)); 2.64 (2 H, t, $^3J_{HH}$ = 6.48 Hz, CH_2 (Lev)); 2.76 (2 H, m, CH_2 (Lev)); 3.65 (2 H, m, CH_2 (5′)); 4.14 (1 H, dd, $^3J_{HH}$ = 5.72 Hz, $^3J_{HH}$ = 3.36 Hz, CH (4′)); 5.36 (1 H, t, $^3J_{HH}$ = 5.32 Hz, OH (5′)); 5.41 (1 H, dd, $^3J_{HH}$ = 5.36 Hz, $^3J_{HH}$ = 2.12 Hz, CH (3′)); 5.68 (1 H, dd, $^3J_{HH}$ = 7.04 Hz, $^3J_{HH}$ = 5.52 Hz, CH (2′)); 5.97 (1 H, d, $^3J_{HH}$ = 7.24 Hz, CH (1′)); 6.88 (2 H, s br., NH_2); 8.15 (1 H, s, CH (8)); 12.01 (1 H, s br., NH) ppm. ^{13}C-NMR (d_6-DMSO, 101 MHz): δ 27.21, 27.41

2x CH_2 (Lev); 29.37, 29.57 2x CH_3 (Lev); 37.23, 37.29 2x CH_2 (Lev); 60.96 (m, C (5′)); 70.43 C (3′); 72.91 C (2′); 83.74 C (1′); 83.88 C (4′); 128.20 C (5); 137.98 (8); 147.83 C (4); 153.25 C (2); 171.21, 171.54 2x C=O (Lev); 175.36 C (6); 206.41, 206.76 2x C=O (Lev) ppm.

Note: Identity of 2′,3′-di-*O*-levulinoyl-N^2,5′-*O*-di-(4,4′-dimethoxytrityl)-6-thioguanosine was confirmed by HR-ESI-MS (m/z): $[M+H]^+$ calculated for $[C_{62}H_{62}N_5O_{12}S]^+$: 1100.4110; found: 1100.4075. TLC (5% MeOH in CH_2Cl_2): 0.40.

^{1}H-NMR (400 MHz, d_6-DMSO) of compound **8**

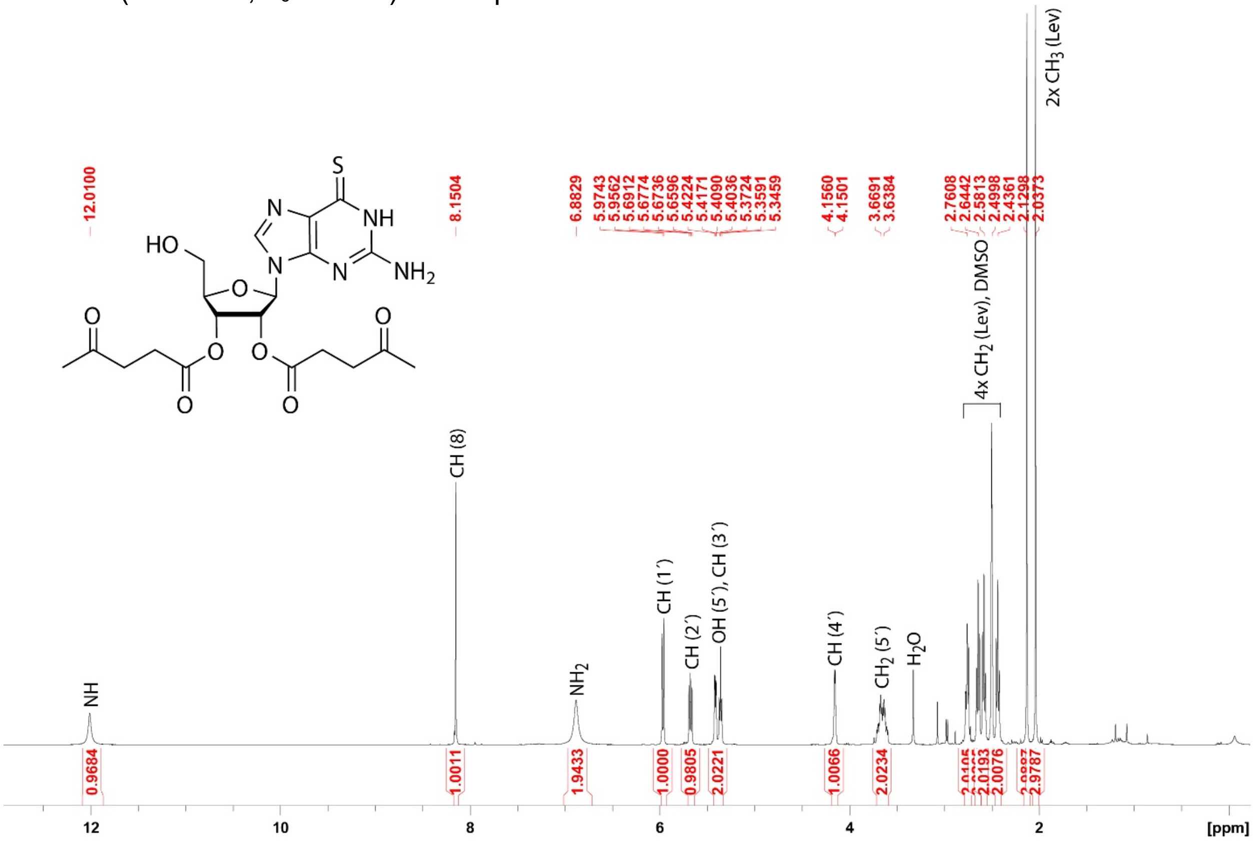

^{13}C-NMR (101 MHz, d_6-DMSO) of compound **8**

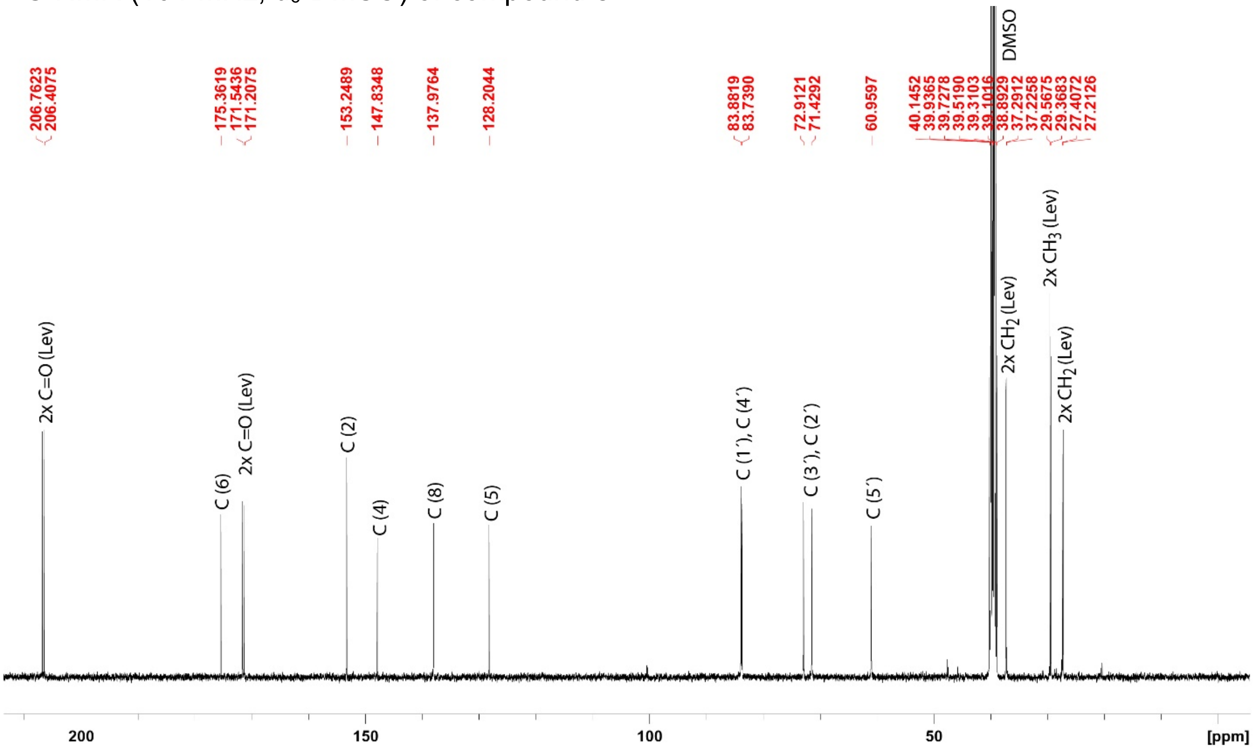

4.4. Synthesis of *cyclo*saligenyl-5′-*O*-(2′,3′-di-*O*-levulinoyl-6-thioguanosinyl) phosphate (9a)

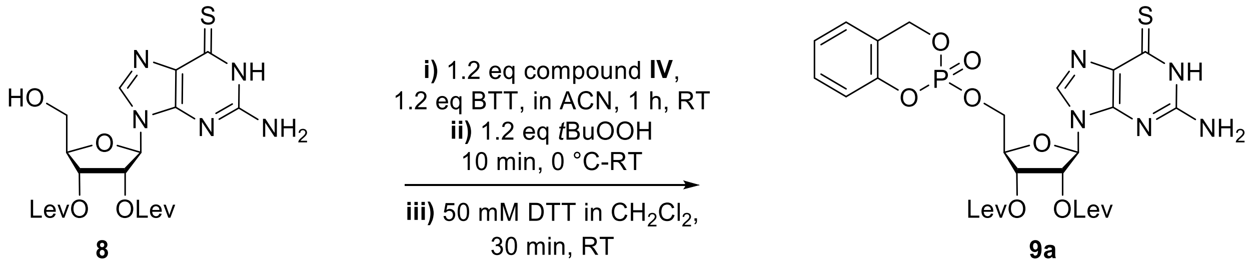

Compound **8** (102 mg, 0.21 mmol) was coevaporated trice with acetonitrile and was subsequently dried under high vacuum for 30 min. It was then suspended in acetonitrile (0.82 ml) and 0.3 M 5-(benzylthio)-1*H*-tetrazole in acetonitrile (1.2 eq, 0.25 mmol, 0.82 ml) and compound **IV**[1] (1.2 eq, 0.25 mmol, 62 mg) were added consecutively. The resulting solution was stirred 1 h at ambient temperature and was then cooled to 0 °C. A 0.5 M solution of *tert*-butyl hydroperoxide (in decane) in toluol (1.2 eq, 0.25 mmol, 0.49 ml) was added dropwise and stirring was continued for 5 min at 0 °C and for further 5 min at ambient temperature. Solvent was removed *in vacuo* and the oily residue was dissolved in dichloromethane containing 50 mM DL-dithiothreitol (2.0 ml). The reaction mixture was stirred another 30 min at ambient temperature followed by evaporation of the solvent and purification via column chromatography (1-5% MeOH in CH_2Cl_2). Yield: 93 mg as a white foam (68%). TLC (5% MeOH in CH_2Cl_2): 0.32. HR-ESI-MS (m/z): $[M+H]^+$ calculated for $[C_{27}H_{31}N_5O_{11}PS]^+$: 664.1473; found: 664.1459. ^{1}H-NMR ($CDCl_3$, 400 MHz): δ 2.15, 2.18 (2x 3 H, 2x s, 2x CH_3 (Lev)); 2.58-2.68, 2.70-2.81 (8 H, m, 4x CH_2 (Lev)); 4.39 (1 H, m, CH (4′)); 4.43-4.66 (2 H, m, CH_2 (5′)); 5.32 (2 H, m, $POCH_2$); 5.76 (1 H, m, CH (3′)); 5.92 (2 H, m, CH (2′), CH (1′); 6.48, 6.53 (2 H, 2x s, $NH_{2\ a,b}$); 6.94-7.08 (3 H, m, CH_{aryl} (3), CH_{aryl} (6), CH_{aryl} (5)); 7.24 (1 H, m, CH_{aryl} (4)); 8.10, 8.11 (1 H, 2x s, $CH_{a,b}$ (8)); 12.04 (1 H, s, NH) ppm. ^{13}C-NMR ($CDCl_3$, 101 MHz): δ 27.64, 27.71, 27.75 2x CH_2 (Lev); 29.96 2x CH_3 (Lev); 37.83, 37.91, 37.94 2x CH_2 (Lev); 66.95 (m, C (5′)); 69.10 (m, $POCH_2$); 70.41, 70.49 C (3′); 72.88 C (2′); 80.75 (m, C (4′)); 87.37, 87.43 C (1′); 118.63 (m, C_{aryl} (3)); 120.61 (m, C_{aryl} (1)); 124.70, 124.72 C_{aryl} (5); 125.52, 125.61 C_{aryl} (6); 129.71, 129.76 C (5); 130.01, 130.10 C_{aryl} (4); 140.43, 140.52 (8); 147.33 C (4); 149.85 (d, $^2J_{CP}$ = 7.27 Hz, C_{aryl} (2)); 149.92 (d, $^3J_{CP}$ = 7.32 Hz, C_{aryl} (2)); 152.99, 153.02 C (6) or C (2); 171.71, 171.72, 171.93, 171.96 2x C=O (Lev); 175.71 C (6) or C (2); 206.76, 206.82, 206.83 2x C=O (Lev) ppm. ^{31}P-NMR ($CDCl_3$, 162 MHz): δ -9.34, -9.13 ppm (2x diastereomers).

^{1}H-NMR (400 MHz, $CDCl_3$) of compound **9a**

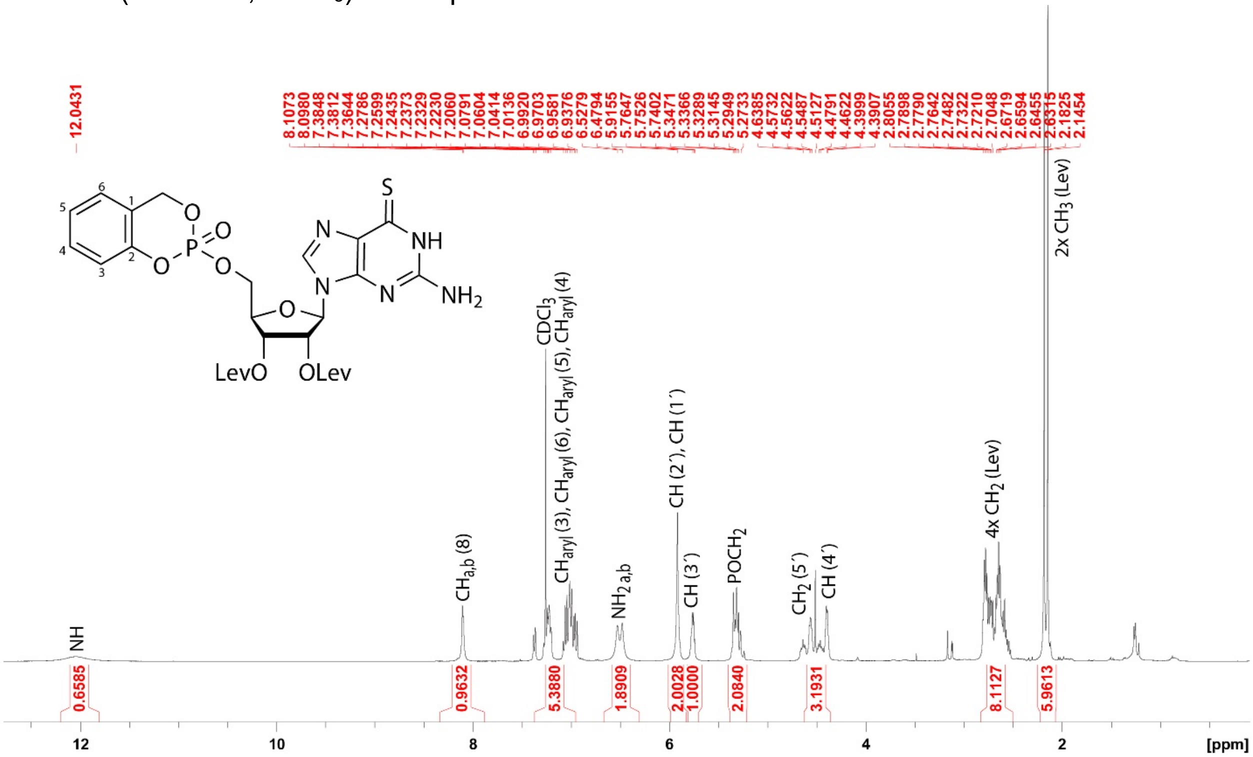

^{13}C-NMR (101 MHz, $CDCl_3$) of compound **9a**

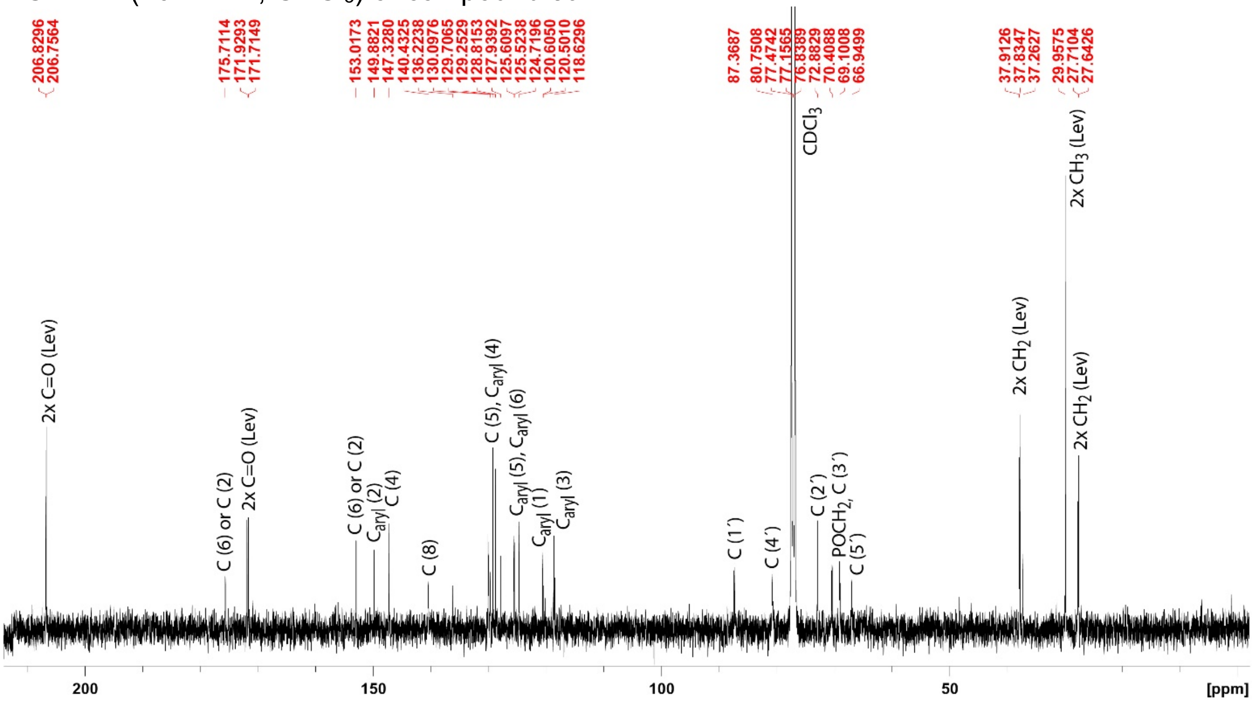

^{31}P-NMR (162 MHz, $CDCl_3$) of compound **9a**

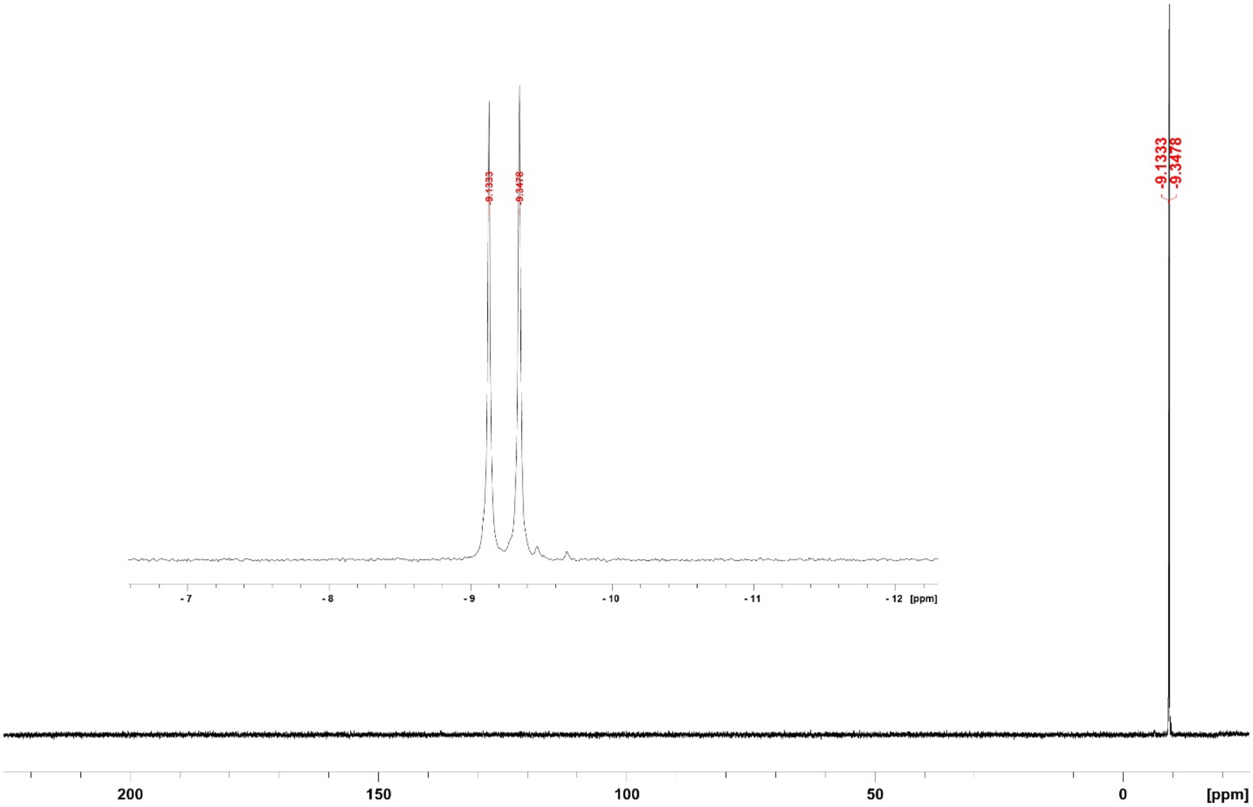

4.5. Synthesis of *cyclo*(5-chlorosaligenyl)-5′-*O*-(2′,3′-di-*O*-levulinoyl-6-thioguanosinyl) phosphate (9b)

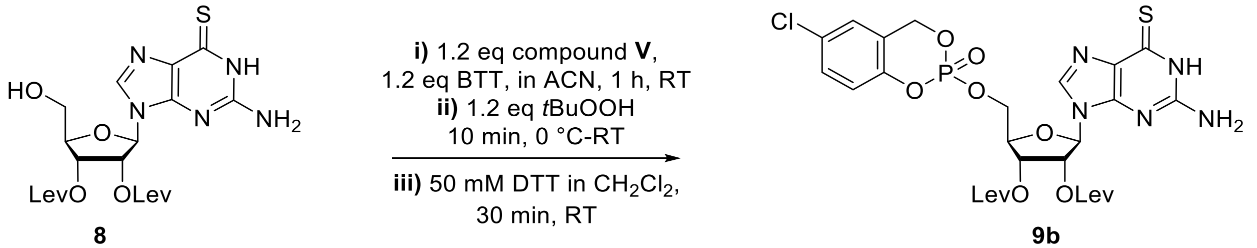

Compound **8** (105 mg, 0.21 mmol) was coevaporated trice with acetonitrile and was subsequently dried under high vacuum for 30 min. It was then suspended in acetonitrile (0.85 ml) and 0.3 M 5-(benzylthio)-1*H*-tetrazole in acetonitrile (1.2 eq, 0.25 mmol, 0.85 ml) and compound **V**[1] (1.2 eq, 0.25 mmol, 73 mg) were added consecutively. The resulting solution was stirred 1 h at ambient temperature and was then cooled to 0 °C. A 0.5 M solution of *tert*-butyl hydroperoxide (in decane) in toluol (1.2 eq, 0.25 mmol, 0.51 ml) was added dropwise and stirring was continued for 5 min at 0 °C and for further 5 min at ambient temperature. Solvent was removed *in vacuo* and the oily residue was dissolved in dichloromethane containing 50 mM DL-dithiothreitol (2.0 ml). The reaction mixture was stirred another 30 min at ambient temperature followed by evaporation of the solvent and purification via column chromatography (1-5% MeOH in CH_2Cl_2). Yield: 52 mg as colorless crystals (57%). TLC (5% MeOH in CH_2Cl_2): 0.42. HR-ESI-MS (m/z): $[M+H]^+$ calculated for $[C_{27}H_{30}ClN_5O_{11}PS]^+$: 698.1083; found: 698.1072. ^{1}H-NMR (CDCl$_3$, 400 MHz): δ 2.15, 2.19 (2x 3 H, 2x s, 2x CH_3 (Lev)); 2.58-2.79 (8 H, m, 4x CH_2 (Lev)); 4.40 (1 H, m, CH (4′)); 4.44-4.66 (2 H, m, CH_2 (5′)); 5.19-5.32 (2 H, m, $POCH_2$); 7.74 (1 H, m, CH (3′)); 5.92 (2 H, m, CH (2′), CH (1′)); 6.51 (2 H, s br., NH_2); 6.89 (0.5 H, d, $^3J_{HH}$ = 8.78 Hz, $CH_{aryl,a}$ (3)); 6.95 (0.5 H, d, $^3J_{HH}$ = 8.83 Hz, $CH_{aryl,b}$ (3)); 7.01 (0.5 H, d, $^4J_{HH}$ = 1.95 Hz, $CH_{aryl,a}$ (6)); 7.03 (0.5 H, d, $^4J_{HH}$ = 2.00 Hz, $CH_{aryl,b}$ (6)); 7.21 (1 H, m, CH_{aryl} (4)); 8.06 (1 H, s, CH (8)) ppm. ^{13}C-NMR (CDCl$_3$, 101 MHz): δ 27.63, 27.70 2x CH_2 (Lev); 29.95 2x CH_3 (Lev); 37.82, 37.90 2x CH_2 (Lev); 67.26 (m, C (5′)); 68.37-68.60 (2x d, $POCH_2$); 70.47, 70.51 C (3′); 72.88 C (2′); 80.87 (m, C (4′)); 80.40 C (1′); 120.10 (m, C_{aryl} (3)); 122.08 (m, C_{aryl} (1)); 125.60, 125.63 (2x s, C_{aryl} (6)); 129.66, 129.71 C (5); 129.836, 129.845 (2x s, C_{aryl} (4)); 130.04, 130.09 (2x s, C_{aryl} (5)); 136.27 C (8); 147.28 C (5); 148.45 (d, $^2J_{CP}$ = 6.54 Hz, C_{aryl} (2)); 148.51 (d, $^3J_{CP}$ = 6.42 Hz, C_{aryl} (2)); 153.05 C (2) or C (6); 171.74, 171.97, 171.99 2x C=O (Lev); 175.66 C (2) or C (6); 206.79, 206.86, 206.88 2x C=O (Lev) ppm. ^{31}P-NMR (CDCl$_3$, 162 MHz): δ -9.69, -9.50 ppm (2x diastereomers).

^{1}H-NMR (400 MHz, $CDCl_3$) of compound **9b**

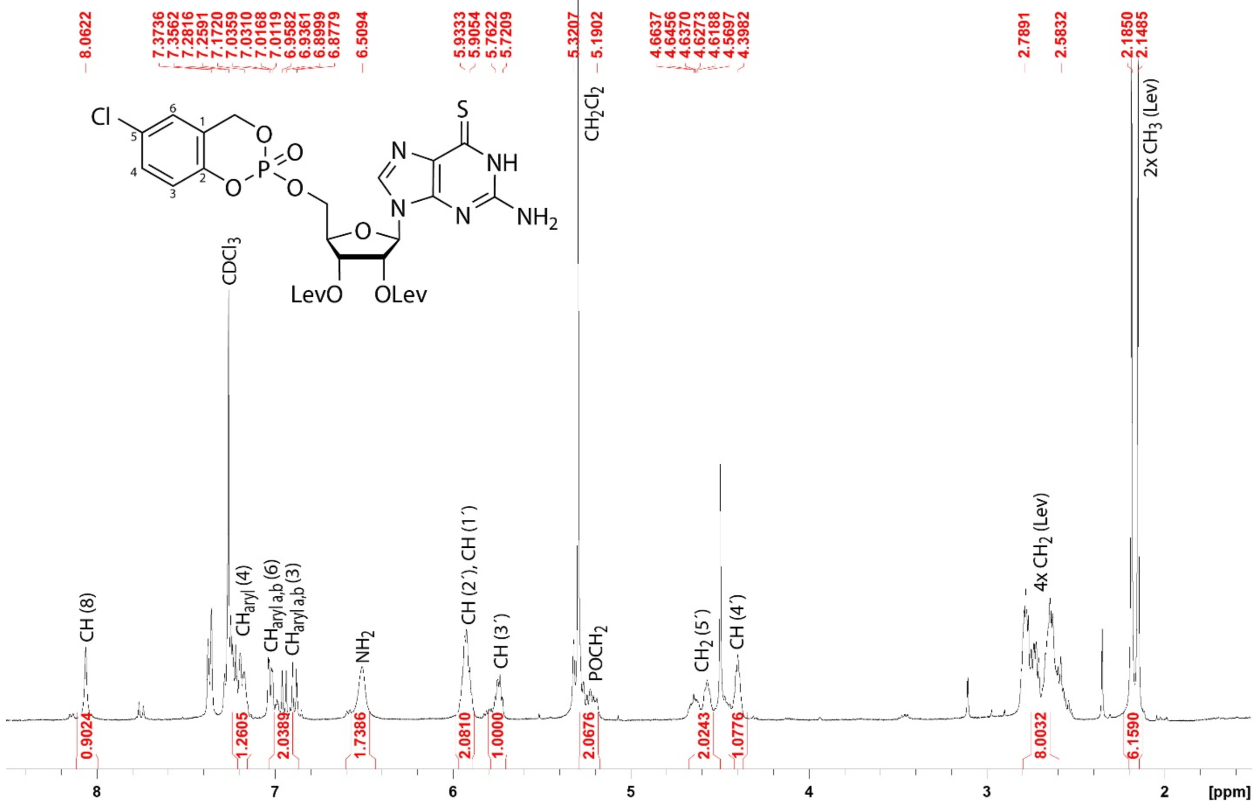

^{13}C-NMR (101 MHz, $CDCl_3$) of compound **9b**

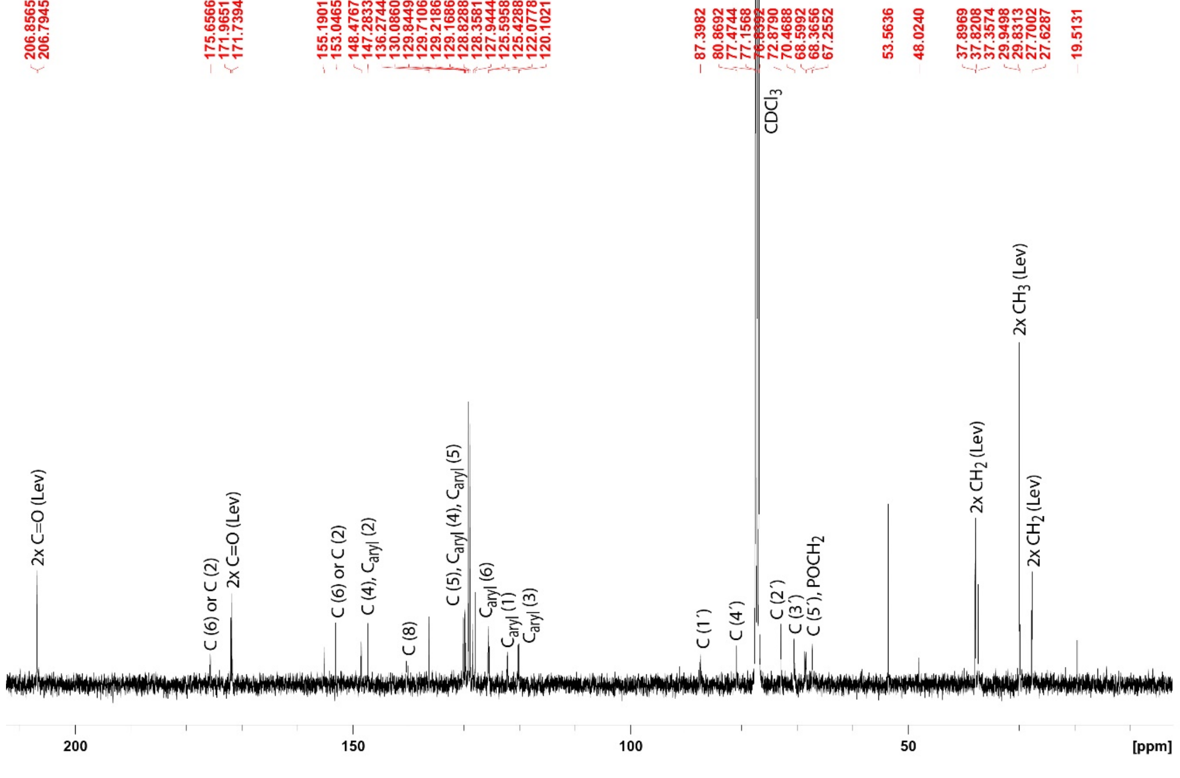

^{31}P-NMR (162 MHz, $CDCl_3$) of compound **9b**

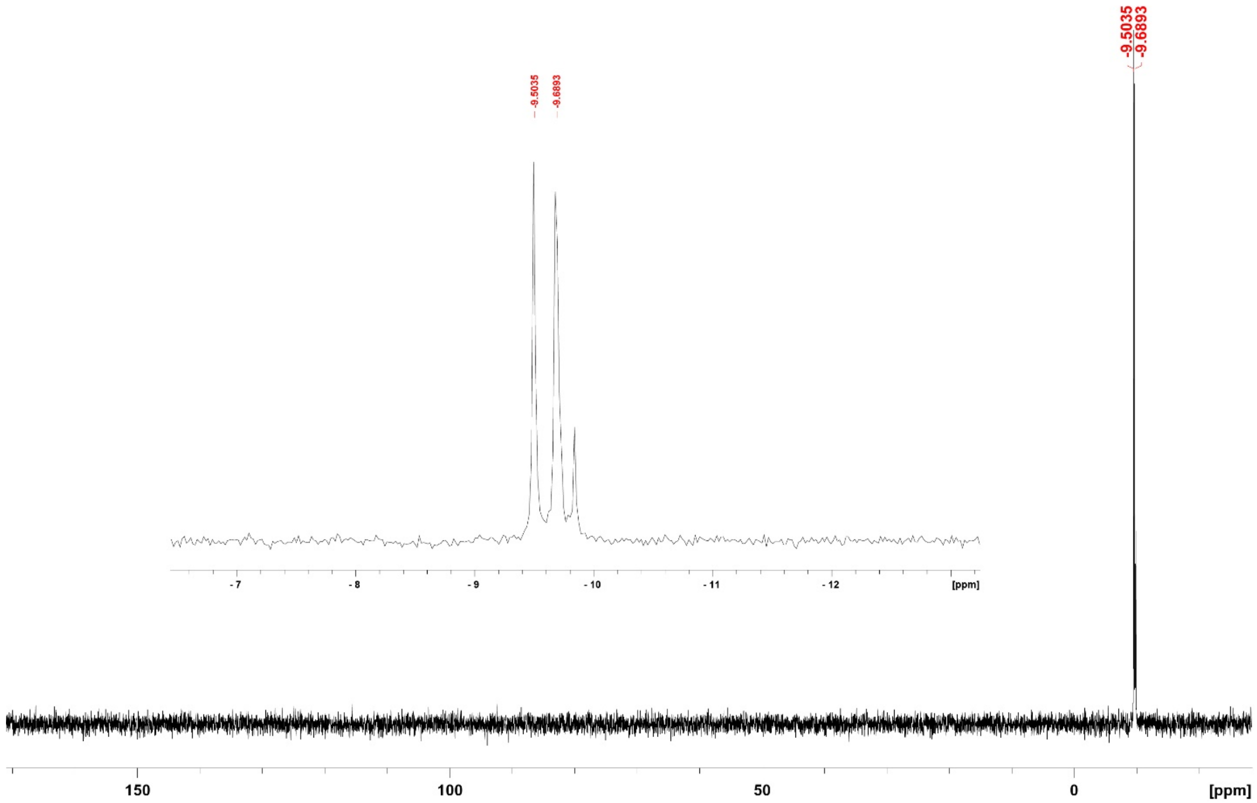

4.6. Synthesis of 2-ethylbutyl-2-(5′-*O*-(2′,3′-di-*O*-levulinoyl-6-thioguanosinyl)(phenoxy) phosphoryl) alaninate (9c)[8]

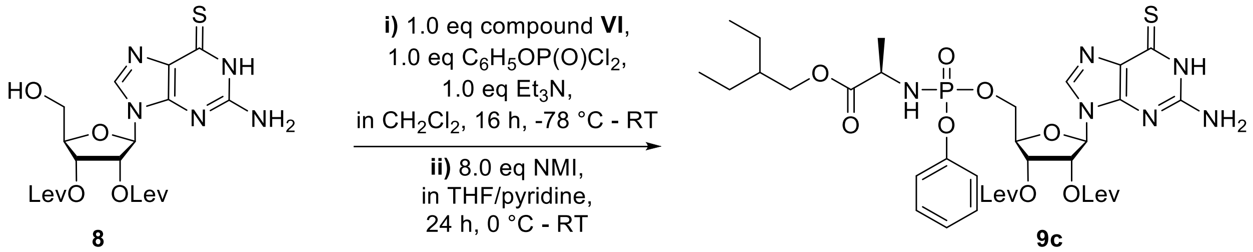

Compound **VI**[1] (268 mg, 1.55 mmol, dried *in vacuo* overnight at 50 °C) in dichloromethane (4.8 ml) was treated with phenyl-dichlorophosphate (1.0 eq, 1.55 mmol, 326 mg) at 0 °C. The reaction mixture was further cooled to -78 °C where triethylamine (1.0 eq, 1.55 mmol, 157 mg) was introduced dropwise over a period of 30 min. The reaction mixture was stirred for 2 more hours at -78 °C and was then allowed to warm to ambient temperature overnight. Solvent was evaporated on a pre-dried rotavapor which was aerated with argon gas. The remnant was dissolved in diethyl ether (5.0 ml) and salt were removed by filtration via Schlenk-frit. Evaporation of the solvent delivered the 2-ethylbutyl (chloro(phenoxy)phosphoryl)alaninate as a colorless oil which was used without further purificartion. Compound **8** (124 mg, 0.25 mmol) was suspended in tetrahydrofuran (9.0 ml) was stirred for 1 h at ambient temperature. 2-ethylbutyl (chloro(phenoxy)phosphoryl)alaninate (5.0 eq, 1.25 mmol, 435 mg) in tetrahydrofuran (1.72 ml) was added followed by the dropwise addition of *N*-methyl imidazole (8.0 eq, 2.00 mmol, 0.16 ml). Pyridine (6.0 ml) was added after 6 h to improve solubility of the starting material. Stirring was continued for another 18 h followed by termination of the reaction by the addition of methanol. Solvent was removed under reduced pressure and the residue was dissolved in ethyl acetate. Extraction with brine delivered the crude product, which was purified by column chromatography (0-5% MeOH in CH_2Cl_2). Yield: 90 mg as a white foam (45%). TLC (5% MeOH in CH_2Cl_2): 0.48. HR-ESI-MS (m/z): $[M+H]^+$ calculated for $[C_{35}H_{48}N_6O_{12}PS]^+$: 807.2783; found: 807.2762. ^{1}H-NMR ($CDCl_3$, 400 MHz): δ 0.82 (3 H, t, $^3J_{HH}$ = 7.42 Hz, CH_3 (Esther)); 0.83 (3 H, t, $^3J_{HH}$ = 7.35 Hz, CH_3 (Esther)); 1.24-1.32 (4 H, m, 2x CH_2 (Esther)); 1.34-1.39 (3 H, 2x d, CH_3 (Ala)); 1.46 (1 H, sept br., $^3J_{HH}$ = 6.03 Hz, CH (Esther)); 2.15, 2.17, 2.18 (2x 3 H, 3x s, 2x CH_3 (Lev)); 2.50-2.80 (8 H, m, 4x CH_2 (Lev)); 3.11 (0.5 H, dd, $^3J_{HH}$ = 3.64 Hz, $^3J_{HH}$ =1.19 Hz, NH_a); 3.16 (0.5 H, d, $^3J_{HH}$ = 2.68 Hz, NH_b); 3.92-4.09 (3 H, m, OCH_2 (Esther), CH (Ala)); 4.34-4.46 (2 H, m, CH (4′), CH_a (5′)); 4.54 (1

H, m, CH_b (5′)); 5.69 (1 H, m, CH (3′)); 5.82 (1 H, m, CH (2′)); 5.91 (1 H, d, $^3J_{HH}$ = 5.75 Hz, CH (1′)); 6.33, 6.37 (2 H, 2x s br. $NH_{2\,a,b}$); 7.04-7.21, 7.25-7.29 (5 H, m, aromat. CH); 7.96 (0.5 H, s, CH_a (8)); 8.06 (0.5 H, s, CH_b (8)) ppm. ^{13}C-NMR ($CDCl_3$, 101 MHz): δ 11.02, 11.05 2x CH_3 (Esther); 20.88-20.97 (2x d, CH_3 (Ala)); 23.19, 23.22 2x CH_2 (Esther); 27.56, 27.71 2x CH_2 (Lev); 29.90 2x CH_3 (Lev); 37.78, 37.87 2x CH_2 (Lev); 40.23 CH (Esther); 50.48(d br., $^2J_{CP}$ = 11.50 Hz, CH (Ala)); 65.90 (m, C (5′)); 67.69, 67.75 OCH_2 (Esther); 70.94 C (3′); 72.64, 72.72 C (2′); 81.21-81.42 (2x d, C (4′)); 86.80, 86.99 C (1′); 120.23-120.45 (2x d, aromat. C); 125.23, 125.29, 129.42, 129.70 aromat. C; 129.93 C (5); 139.86, 140.14 C (8); 147.34, 147.39 C (4); 150.34-150.49 (2x d, aromat. C_q); 153.02, 153.07 C (2); 171.49, 171.52, 171.82 2x C=O (Lev); 173.60 (d, $^3J_{CP}$ = 7.99 Hz, C=O_a (Ala)); 173.68 (d, $^3J_{CP}$ = 7.99 Hz, C=O_b (Ala)); 175.92, 175.97 C (4); 206.49, 206.65, 206.67 2x C=O (Lev) ppm. ^{31}P-NMR ($CDCl_3$, 162 MHz): δ 3.04, 3.09 ppm (2 diastereomers).

^{1}H-NMR (400 MHz, $CDCl_3$) of compound **9c**

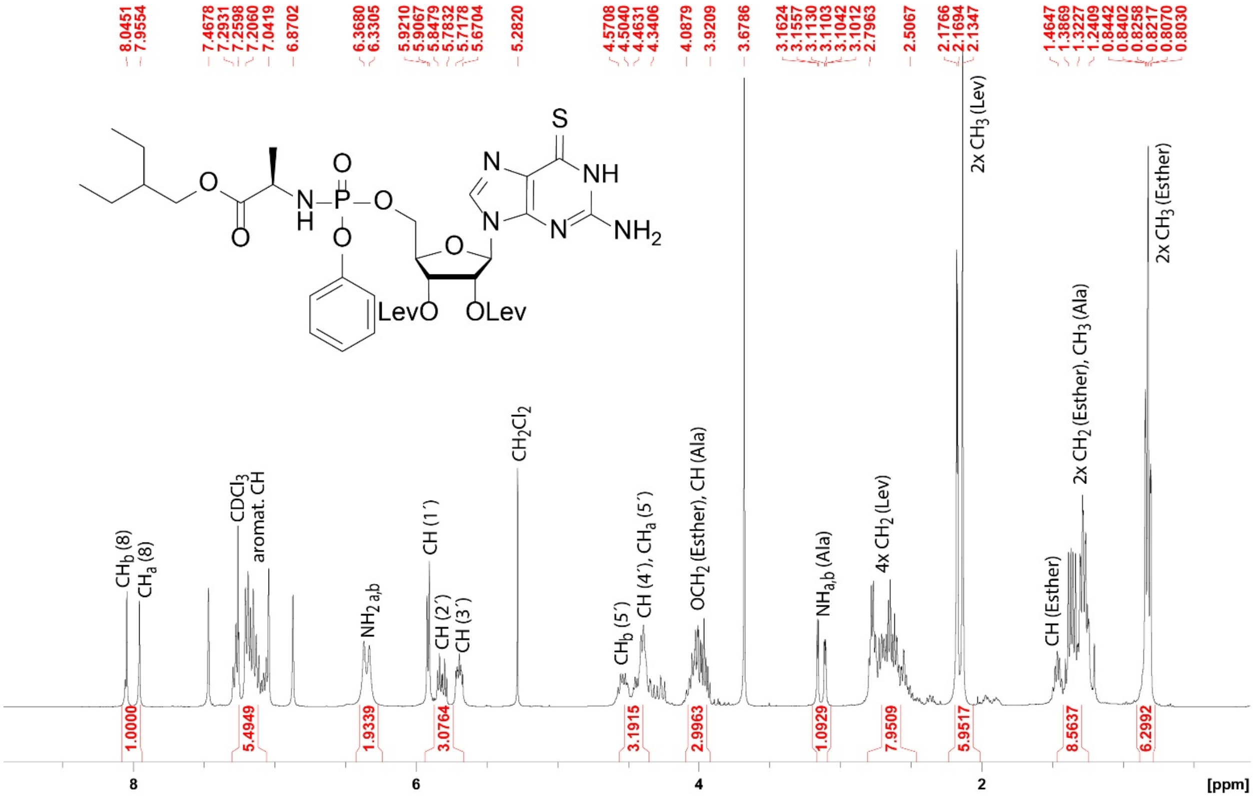

^{13}C-NMR (101 MHz, $CDCl_3$) of compound **9c**

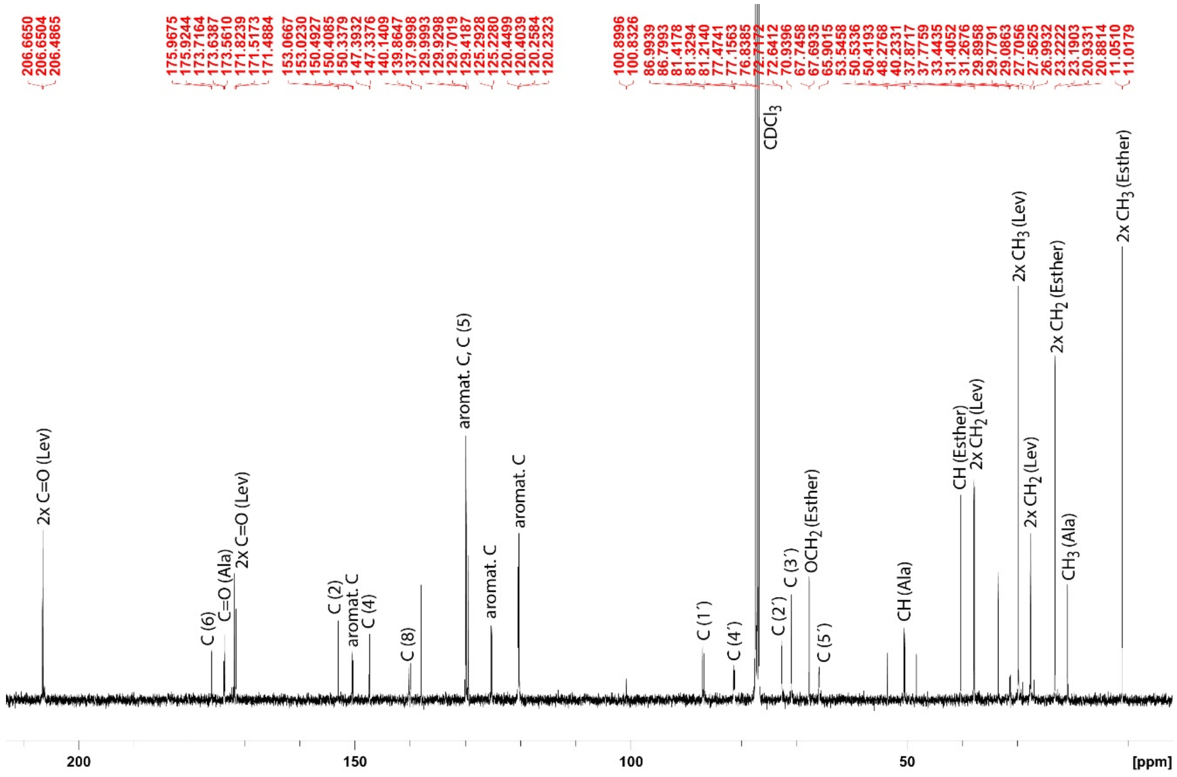

^{31}P-NMR (162 MHz, $CDCl_3$) of compound **9c**

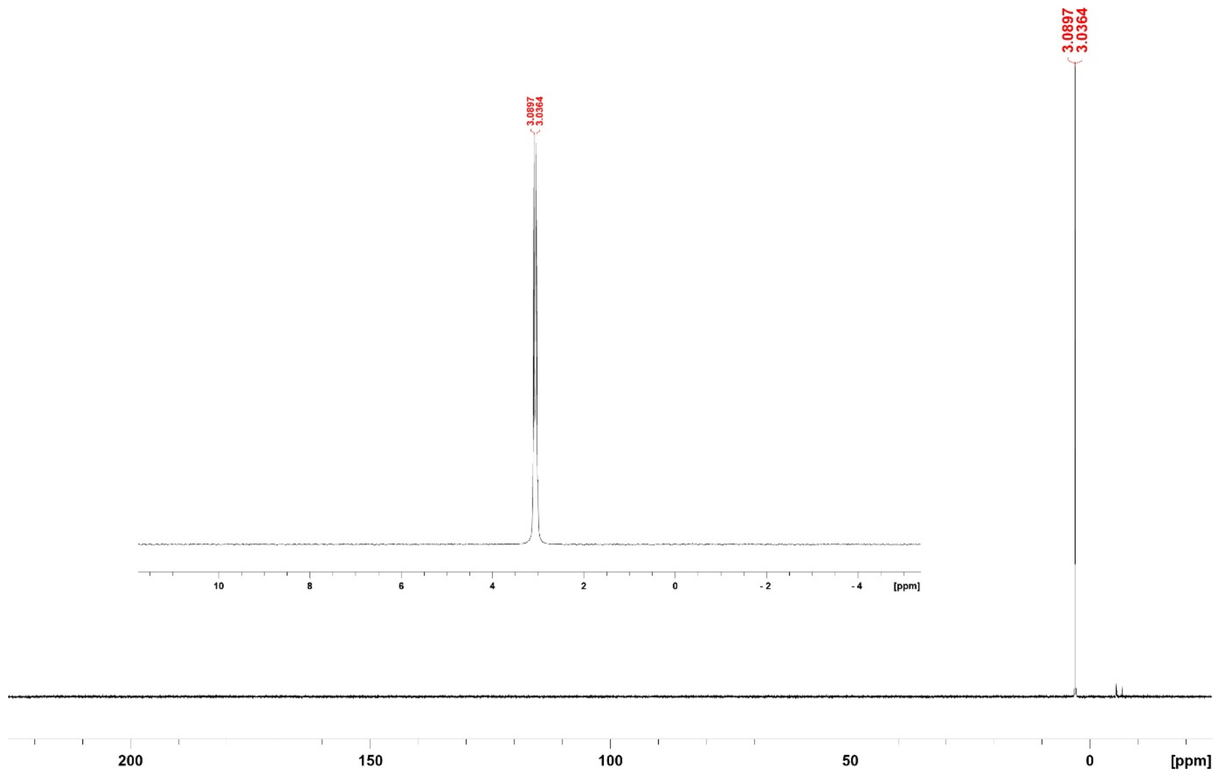

4.7. Synthesis of *cyclo*saligenyl-5′-*O*-(6-thioguanosinyl) phosphate (*cyclo*Sal-6sGMP, 10a)[9]

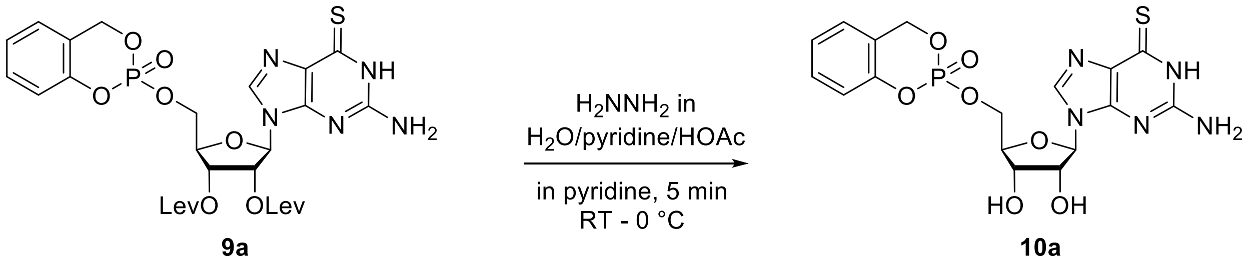

Compound **9a** (79 mg, 0.12 mmol) was dissolved in pyridine (4.2 ml) and treated with hydrazine in a pyridine/ acetic acid- buffer (24- 26% H_2NNH_2 in H_2O/pyridine/acetic acid 2/4/3, 5.58 ml) and was stirred for 5 min. The reaction mixture was then cooled to 0 °C and ethyl acetate (21.0 ml) and water (21.0 ml) were added and stirred vigorously. The organic layer was washed with 5% sodium bicarbonate solution, and was dried over sodium sulfate. Solvent was evaporated and the crude product was coevaporated with toluene. The oily residue was treated with dichloromethane whereas compound **10a** precipitates as a white powder. Yield: 27 mg as white powder (49%). HR-ESI-MS (m/z): $[M+H]^+$ calculated for $[C_{17}H_{19}N_5O_7PS]^+$: 468.0737; found: 468.0734. ^{1}H-NMR (d_6-DMSO, 400 MHz): δ 4.04 (1 H, m, CH (4′)); 4.13 (1 H, m, CH (3′)); 4.26-4.43 (3 H, m, CH_2 (5′), CH (2′)); 5.35-5.51 (3 H, m, OH (3′), $POCH_2$); 5.59 (1 H, d, $^3J_{HH}$ = 5.56 Hz, OH (2′)); 5.70 (1 H, d br., $^3J_{HH}$ = 5.19 Hz, CH (1′)); 6.83 (2 H, s br., NH_2); 7.06 (0.5 H, d, $^3J_{HH}$ = 8.12 Hz, $CH_{aryl,a}$ (3)); 7.12 (0.5 H, d, $^3J_{HH}$ = 8.18 Hz, $CH_{aryl,b}$ (3)); 7.17 (1 H, m, CH_{aryl} (5)); 7.24 (1 H, m, CH_{aryl} (6)); 7.34 (1 H, m, CH_{aryl} (4)); 7.98 (0.5 H, s, CH_a (8)); 8.01 (0.5 H, s, CH_b (8)); 11.98 (1 H, s br., NH) ppm. ^{13}C-NMR (d_6-DMSO, 151 MHz): δ 67.76 (d, $^2J_{CP}$ = 5.51 Hz, C_a (5′)); 67.89 (d, $^2J_{CP}$ = 5.81 Hz, C_b (5′)); 68.37-68.48 (2x d, $POCH_2$); 69.83, 69.85 C (3′); 72.92, 72.94 C (2′); 82.09 (d, $^3J_{CP}$ = 7.03 Hz, C_a (4′)); 82.14 (d, $^3J_{CP}$ = 7.24 Hz, C_b (4′)); 86.75, 86.78 C (1′); 118.12-118.17 (2x d, C_{aryl} (3)); 120.89 (d , $^3J_{CP}$ = 9.90 Hz, $C_{aryl,a}$ (1)); 120.95 (d , $^3J_{CP}$ = 9.91 Hz, $C_{aryl,b}$ (1)); 124.40 C_{aryl} (5); 126.03, 126.04 C_{aryl} (6); 128.38 C (5); 129.73 C_{aryl} (4); 138.30, 138.33 C (8); 147.83 C (4), 149.38 (d, $^2J_{CP}$ = 6.88 Hz, C_{aryl} (2)); 153.06 C (2), 175.19 C (6) ppm. ^{31}P-NMR (d_6-DMSO, 162 MHz): δ -9.85, -9.82 ppm (2 diastereomers).

^{1}H-NMR (600 MHz, d$_6$-DMSO) of compound **10a**

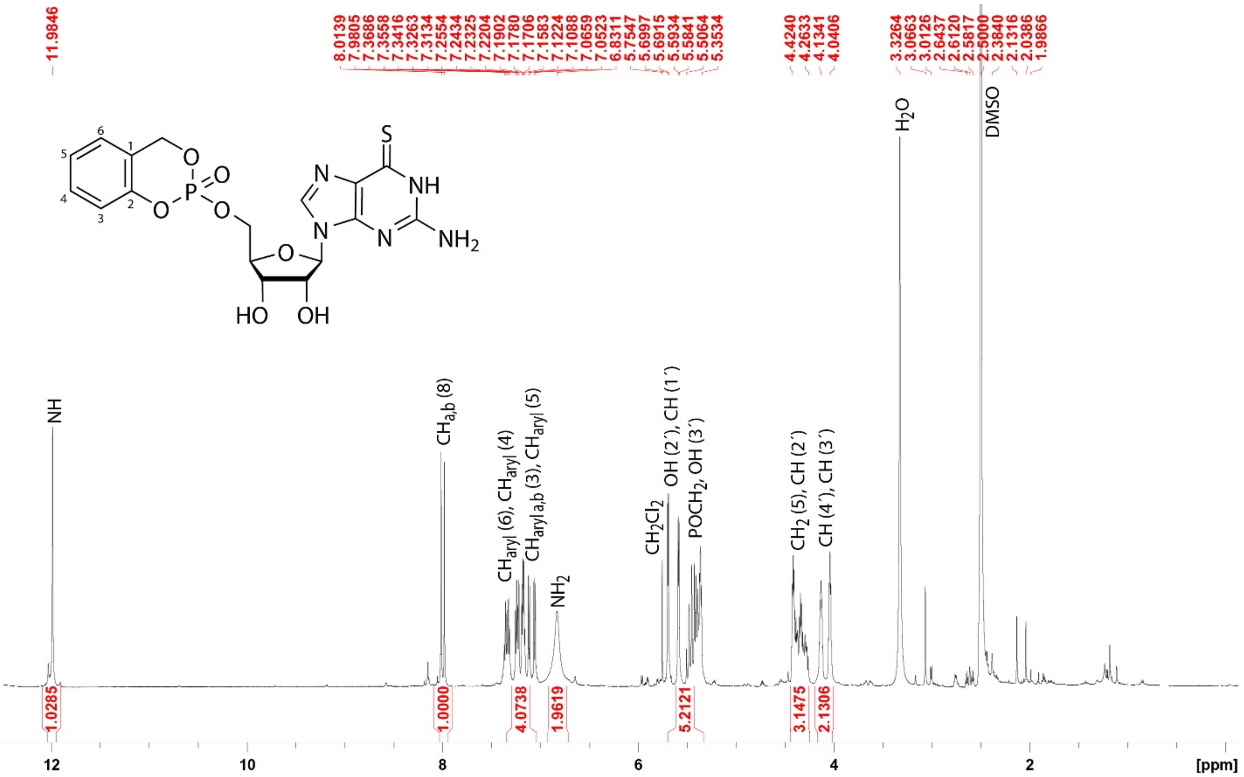

^{13}C-NMR (151 MHz, d$_6$-DMSO) of compound **10a**

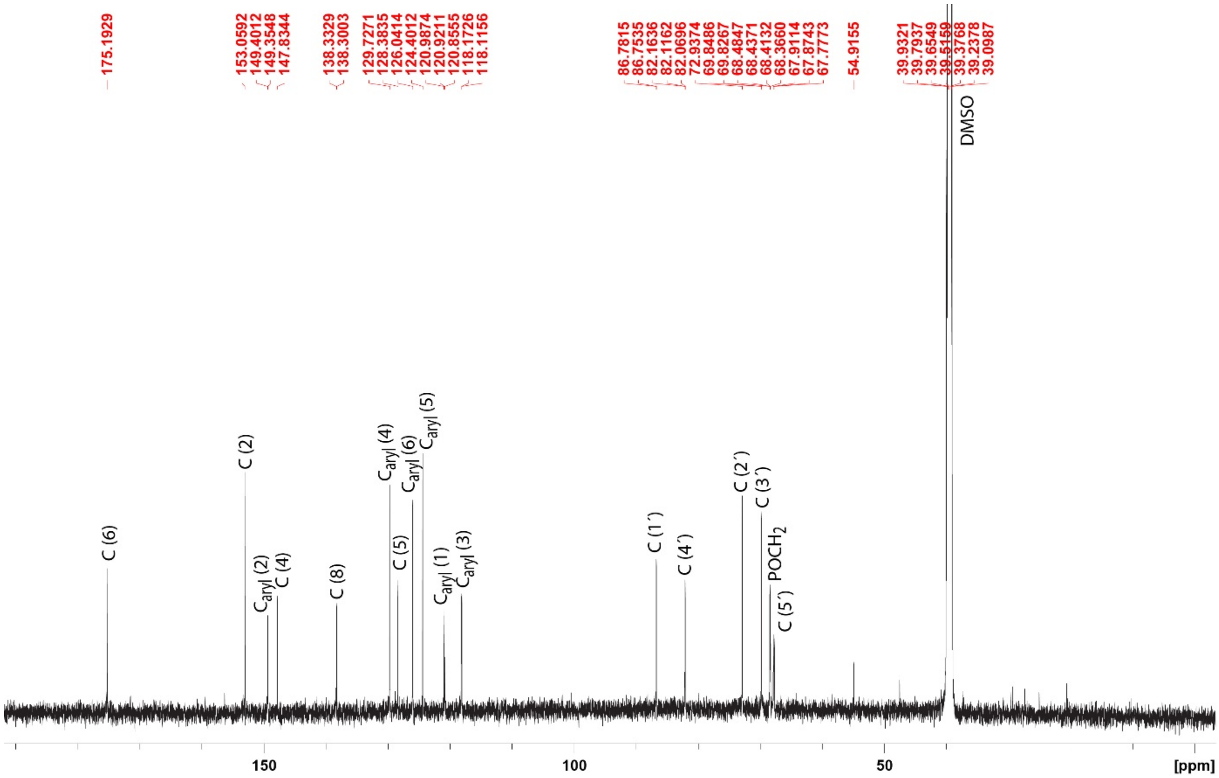

^{31}P-NMR (162 MHz, d_6-DMSO) of compound **10a**

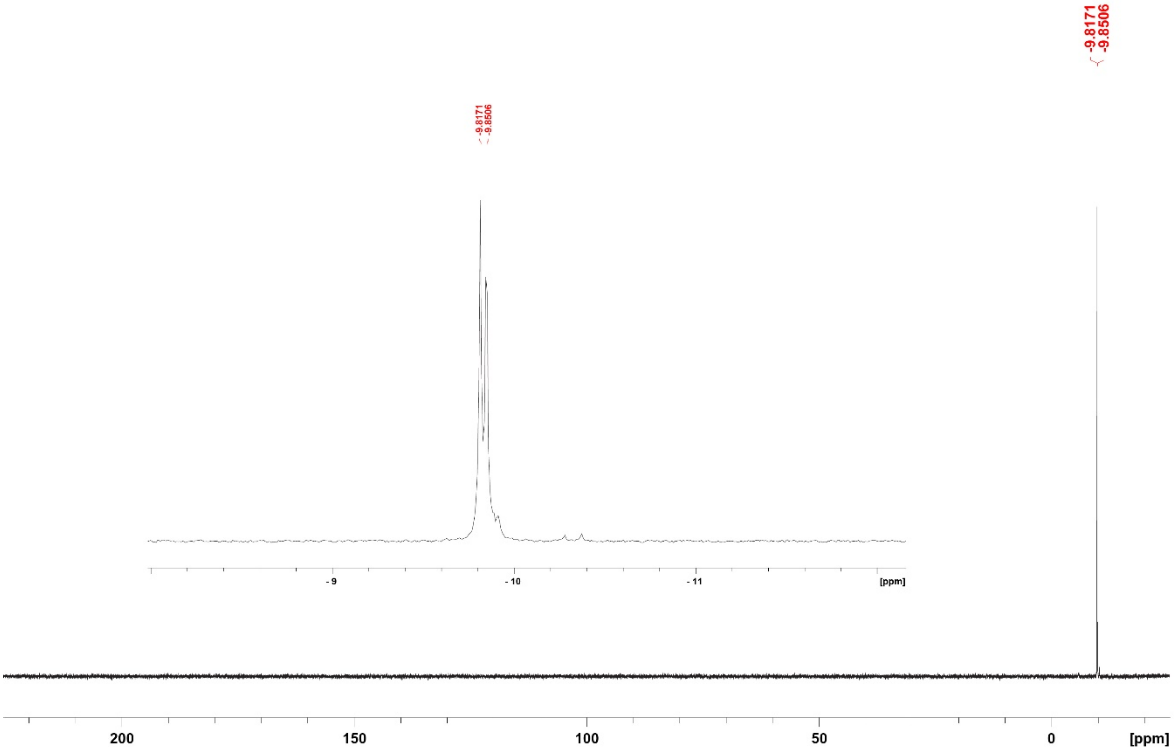

4.8. Synthesis of *cyclo*(5-chlorosaligenyl)-5′-*O*-(6-thioguanosinyl) phosphate (Cl*cyclo*Sal-6sGMP, 10b)[9]

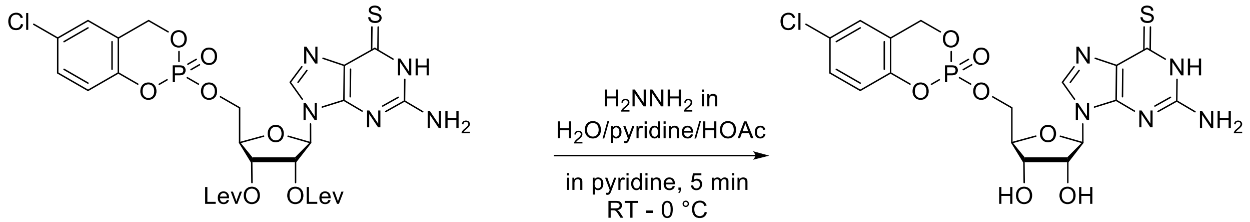

9b **10b**

Compound **9b** (52 mg, 0.074 mmol) was dissolved in pyridine (2.6 ml) and treated with hydrazine in a pyridine/ acetic acid- buffer (24- 26% H_2NNH_2 in H_2O/pyridine/acetic acid 2/4/3, 3.5 ml) and was stirred for 5 min. The reaction mixture was then cooled to 0 °C and ethyl acetate (13.0 ml) and water (13.0 ml) were added and stirred vigorously. The organic layer was washed with 5% sodium bicarbonate solution, and was dried over sodium sulfate. Solvent was evaporated and the crude product was coevaporated with toluene. The oily residue was treated with dichloromethane whereas compound **10b** precipitates as a white powder. Yield: 19 mg as white powder (51%). HR-ESI-MS (m/z): $[M+H]^+$ calculated for $[C_{17}H_{18}ClN_5O_7PS]^+$: 502.0348; found: 502.0361. ^{1}H-NMR (d_6-DMSO, 400 MHz): δ 4.03 (1 H, m, CH (4′)); 4.13 (1 H, m, CH (3′)); 4.31-4.42 (3 H, m, CH_2 (5′), CH (2′)); 5.37-5.50 (3 H, m, OH (3′), $POCH_2$); 5.59 (1 H, d, $^3J_{HH}$ = 4.20 Hz, OH (2′)); 5.69 (1 H, 2x d, $CH_{a,b}$ (1′)); 6.82 (2 H, s br., NH_2); 7.06 (0.5 H, d, $^3J_{HH}$ = 8.58 Hz, $CH_{aryl,a}$ (3)); 7.16 (0.5 H, d, $^3J_{HH}$ = 8.52 Hz, $CH_{aryl,b}$ (3)); 7.33-7.43 (2 H, m, CH_{aryl} (4), CH_{aryl} (6)); 8.02 (0.5 H, s, CH_a (8)); 8.03 (0.5 H, s, CH_b (8)); 11.979, 11.986 (1 H, s br., $NH_{a,b}$) ppm. ^{13}C-NMR (d_6-DMSO, 151 MHz): δ 67.82-68.11 (m, $POCH_2$, C (5′)); 69.82 C (3′); 72.90, 72.96 C (2′); 82.06 (d, $^3J_{CP}$ = 6.43 Hz, C_a (4′)); 82.10 (d, $^3J_{CP}$ = 6.56 Hz, C_b (4′)); 86.71, 86.80 C (1′); 120.01 (d, $^3J_{CP}$ = 8.96 Hz, $C_{aryl,\,a}$ (3)); 120.07 (d, $^3J_{CP}$ = 9.95 Hz, $C_{aryl,\,b}$ (3)); 120.87 (d br., $^3J_{CP}$ = 9.72 Hz, $C_{aryl\ a,b}$ (1)); 125.93 C_{aryl} (6); 128.15, 128.22 C_{aryl} (5); 128.37 C (5); 129.44, 129.55 C_{aryl} (4); 138.35, 138.36 $C_{a,b}$ (8); 147.83, 147.68 $C_{a,b}$ (4), 148.20 (2x d, C_{aryl} (2)); 153.07 C (2), 175.21 C (6) ppm. ^{31}P-NMR (d_6-DMSO, 162 MHz): δ -10.38, -10.28 ppm (2 diastereomers).

^{1}H-NMR (600 MHz, d_6-DMSO) of compound **10b**

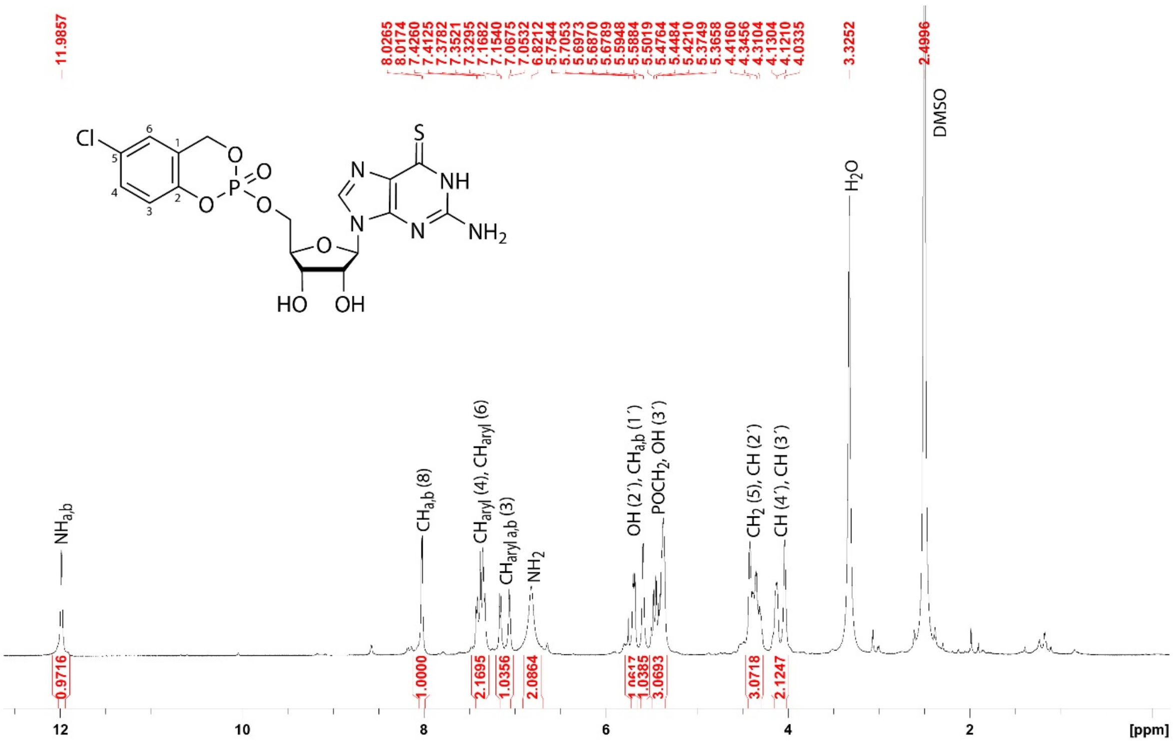

^{13}C-NMR (151 MHz, d_6-DMSO) of compound **10b**

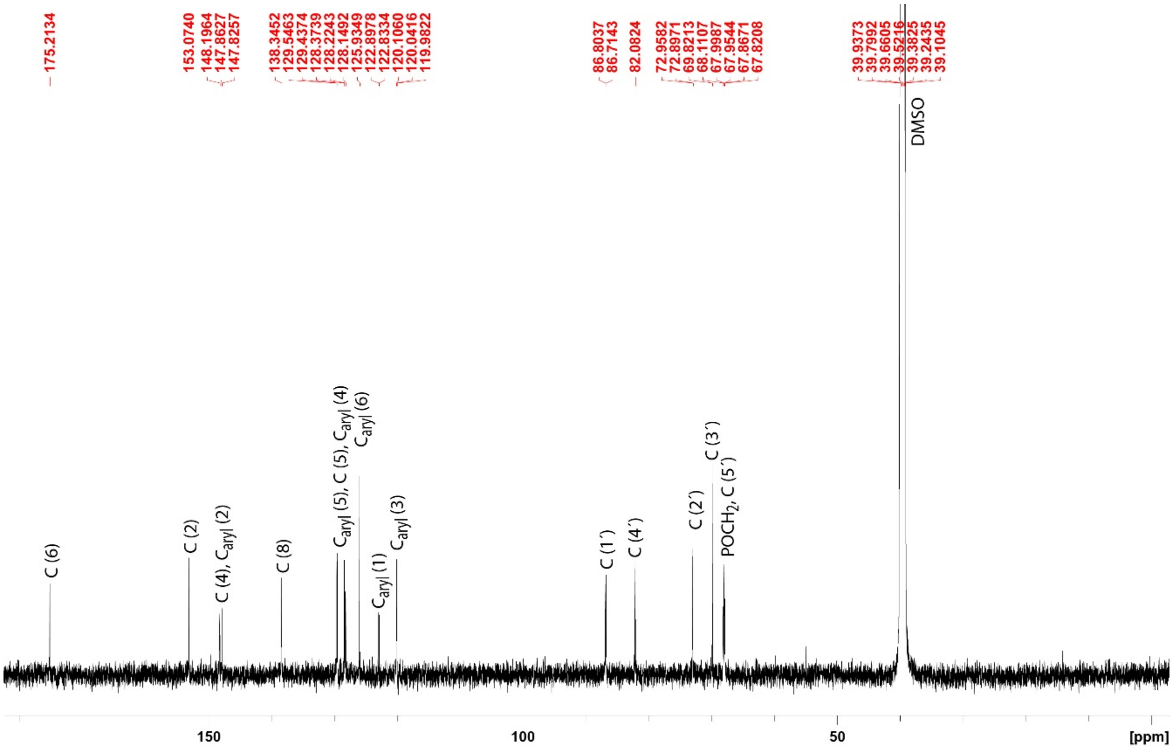

^{31}P-NMR (162 MHz, d_6-DMSO) of compound **10b**

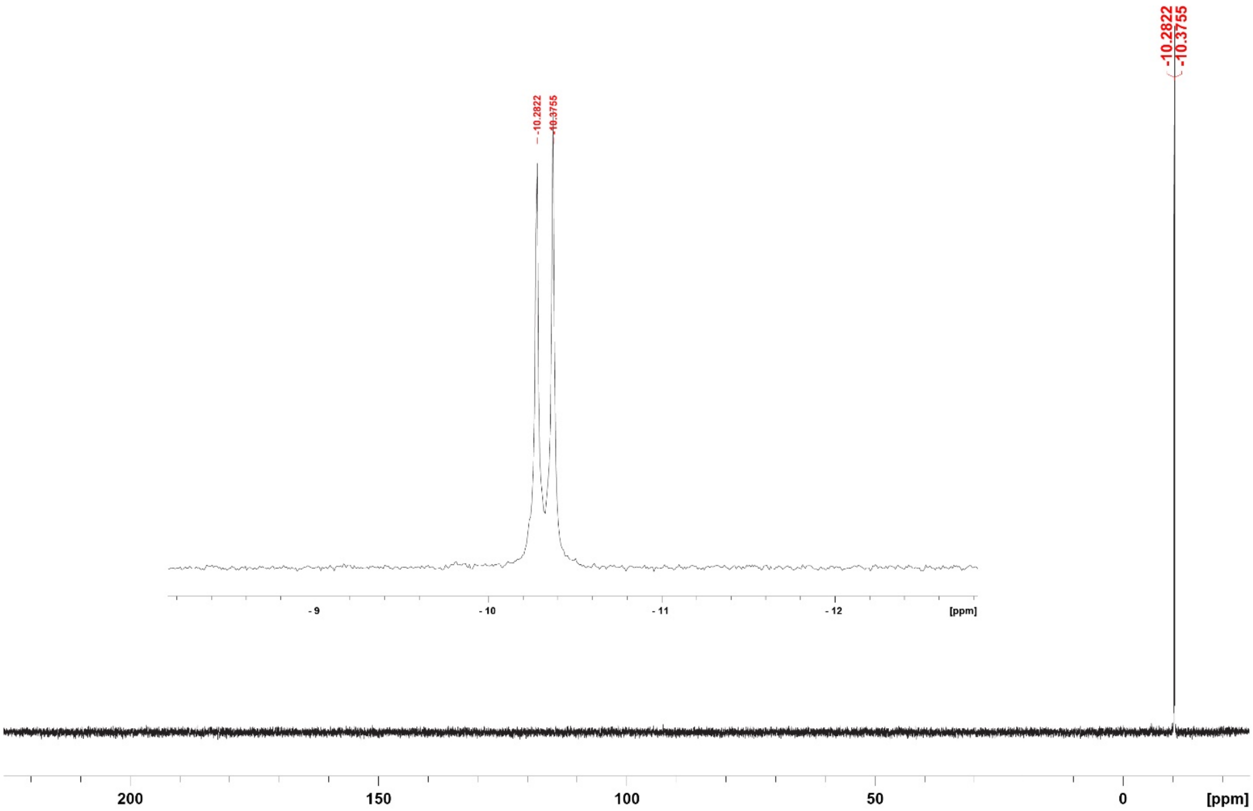

4.9. Synthesis of 2-ethylbutyl-2-(5′-*O*-(6-thioguanosineyl)(phenoxy)phosphoryl) alaninate (ProTide-6sGMP, 10c)[9,10]

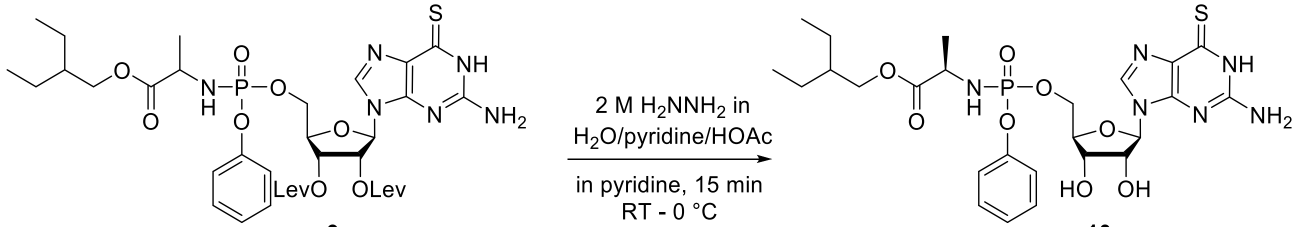

9c **10c**

Compound **9c** (41 mg, 0.052 mmol) was dissolved in pyridine (0.50 ml) and treated with 0.19 ml 2 M hydrazine buffer (hydrazine monohydrate/pyridine/acetic acid 1/5/4). After 10 minutes the solution was cooled to 0 °C and ethyl acetate (2.0 ml) as well as water (2.0 ml) were added and the reaction mixture was stirred vigorously for 5 minutes. The organic phase was separated, washed with 5% sodium bicarbonate solution and evaporated to dryness. The remnant was dissolved in a minimal amount of methanol and the product was precipitated by the addition of diethyl ether. The white powder was collected by centrifugation and was washed 4x with diethyl ether. Yield: 19 mg as white powder (61%). HR-ESI-MS (m/z): $[M+H]^+$ calculated for $[C_{25}H_{36}N_6O_8PS]^+$: 611.2047; found: 611.2043. ^{1}H-NMR (d_6-DMSO, 600 MHz): δ 0.80 (3 H, t, $^3J_{HH}$ = 7.44 Hz, CH_3 (Esther)); 0.81 (3 H, t, $^3J_{HH}$ = 7.08 Hz, CH_3 (Esther)); 1.19-1.24 (4 H, m, 2x CH_2 (Esther)); 1.26 (3 H, m, CH_3 (Ala)); 1.43 (1 H, m, CH (Esther)); 3.79-3.97 (3 H, m, CH (Ala), OCH_2 (Esther)); 4.05-4.26 (4 H, m, CH (4′), CH (3′), CH_2 (5′)); 4.42 (1 H, m, CH (2′)); 5.32 (0.5 H, d, $^3J_{HH}$ = 4.50 Hz, OH_a (3′)); 5.34 (0.5 H, d, $^3J_{HH}$ = 4.44 Hz, OH_b (3′)); 5.56 (1 H, d, $^3J_{HH}$ = 5.58 Hz, OH (2′)); 5.71 (0.5 H, d, $^3J_{HH}$ = 5.82 Hz, CH_a (1′)); 5.73 (0.5 H, d, $^3J_{HH}$ = 5.88 Hz, CH_b (1′)); 6.04 (1 H, q, $^2J_{HP}$ or $^3J_{HH}$ = 22.44 Hz, $^2J_{HP}$ or $^3J_{HH}$ = 10.56 Hz, NH (Ala)); 6.82 (2 H, s br., NH_2); 7.15-7.21, 7.32-7.37 (5 H, m, aromat. CH); 8.03 (0.5 H, s, CH_a (8)); 8.04 (0.5 H, s, CH_b (8)); 11.97 (1 H, s br., NH) ppm. ^{13}C-NMR (d_6-DMSO, 151 MHz): δ 10.75, 10.78 2x CH_3 (Ether); 19.70 (d, $^3J_{CP}$ = 7.33 Hz, $CH_{3,a}$ (Ala)); 19.84 (d, $^3J_{CP}$ = 6.57 Hz, $CH_{3,b}$ (Ala)); 22.52, 22.55 2x CH_2 (Ether); 40.06 CH Esther, 49.86 (d, $^2J_{CP}$ = 11.50 Hz, CH (Ala)); 65.92 (m, C (5′)); 66.12, 66.14 OCH_2 (Esther); 70.17 C (3′); 73.11, 73.18 CH (2′); 82.55-82.64 (2x d, (4′)); 86.43, 86.52 C (1′); 120.11 (d br., $^3J_{CP}$ = 4.47 Hz, aromat. C), 120.14 (d br., $^3J_{CP}$ = 4.35 Hz, aromat. C), 124.54, 124.55 aromat. C; 128.36, 128.38 C (5); 129.55, 129.60 aromat C; 138.19, 138.26 C (8); 147.95 C (4); 150.62-150.69 (2x d, aromat. C_q) 153.09 C (2); 173.18 (d, $^3J_{CP}$ = 5.07 Hz, $C{=}O_a$ (Ala)); 173.32 (d, $^3J_{CP}$ = 4.54 Hz, $C{=}O_b$ (Ala)); 175.20 C (4) ppm. ^{31}P-NMR (d_6-DMSO, 162 MHz): δ 3.79, 3.80 ppm (2 diastereomers).

^{1}H-NMR (600 MHz, $CDCl_3$) of compound **10c**

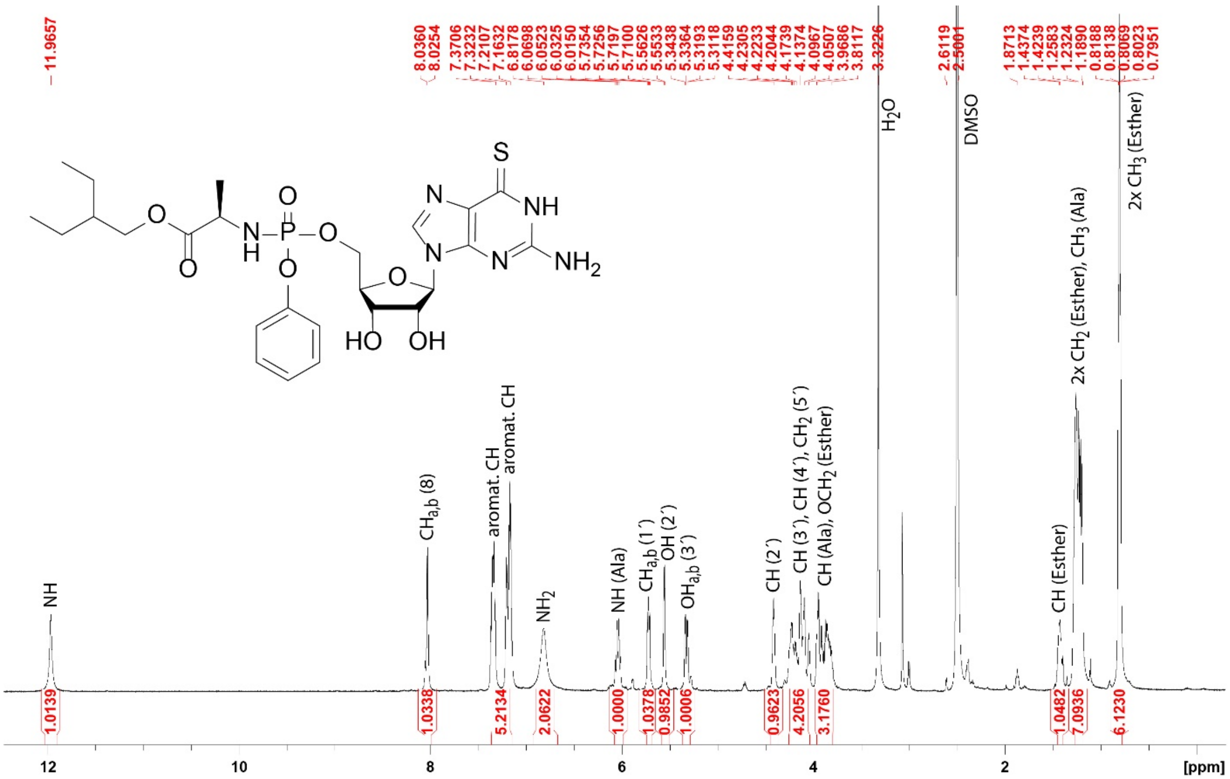

^{13}C-NMR (151 MHz, $CDCl_3$) of compound **10c**

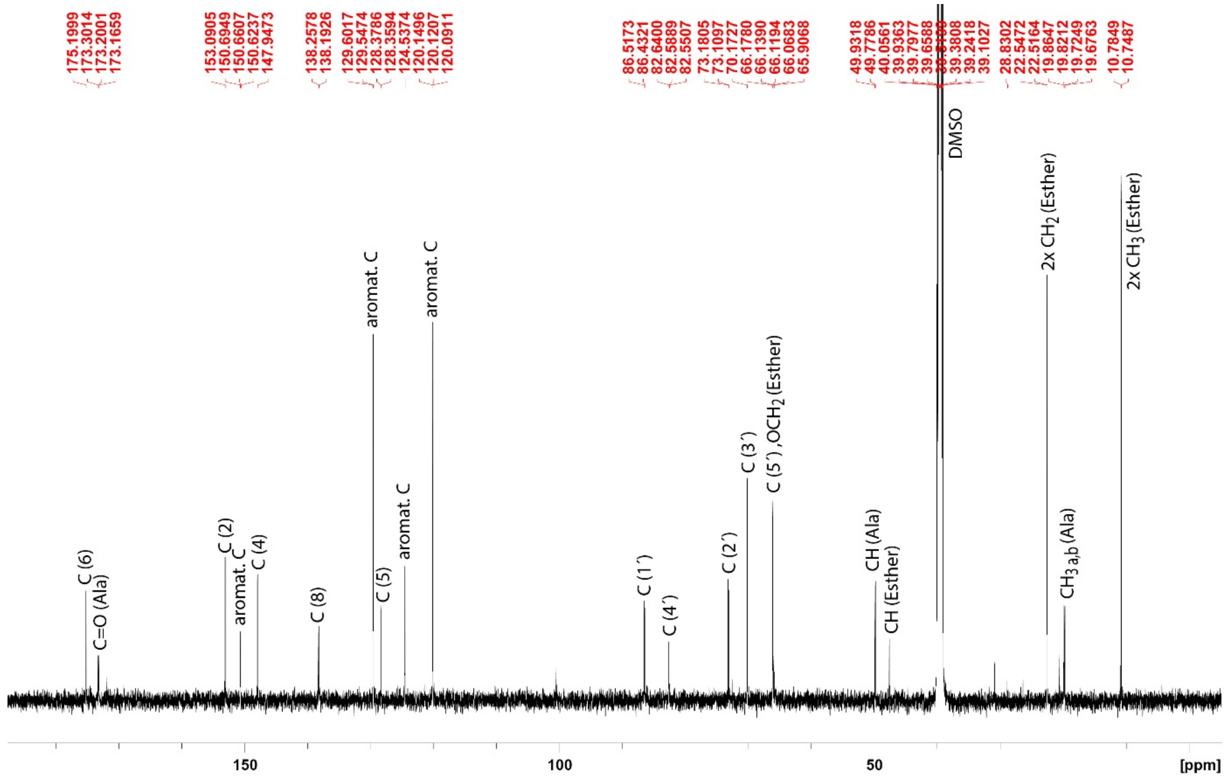

^{31}P-NMR (162 MHz, $CDCl_3$) of compound **10c**

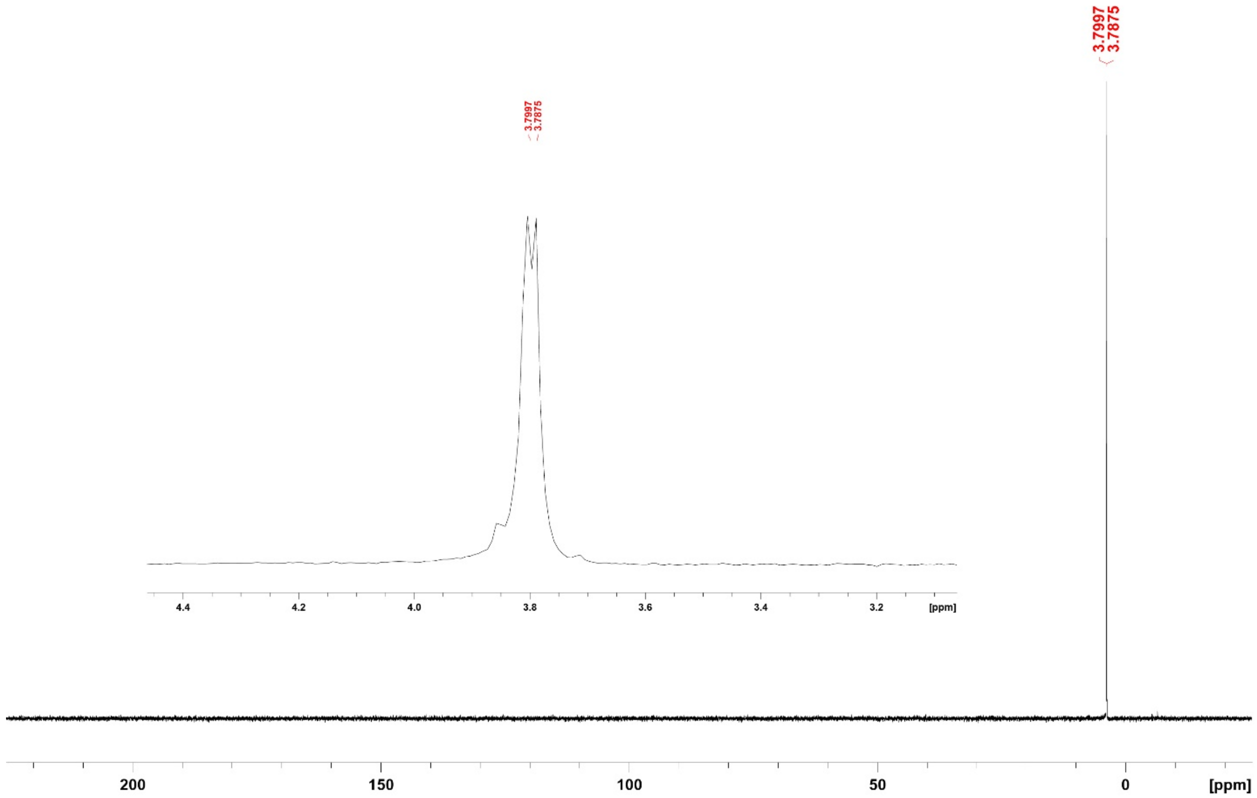

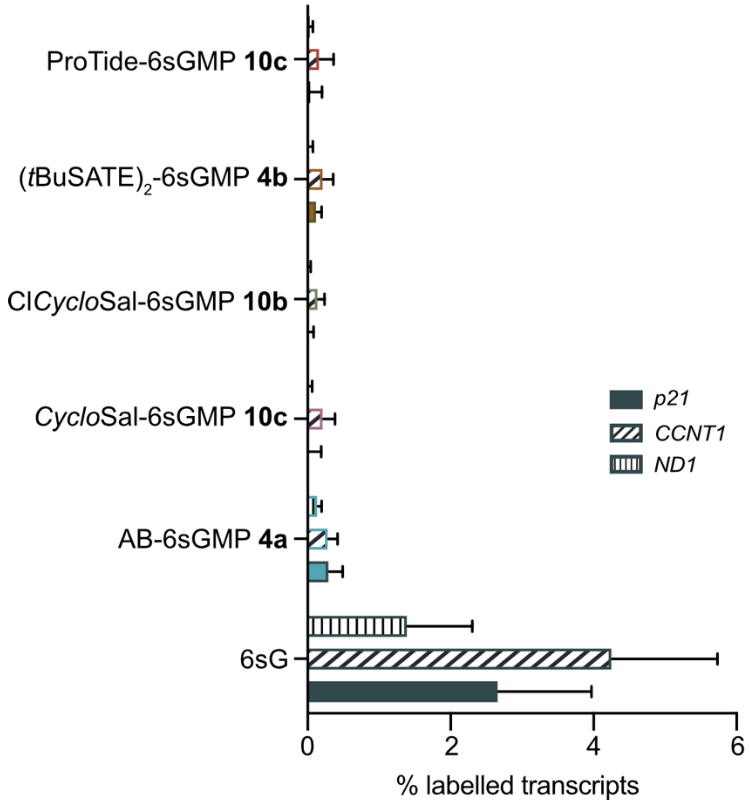

Figure S-1. Short term labeling of HEK293T cells with thioguanosine nucleotides. HEK293T cells were treated with 100 µM of the indicated 6sG prodrugs for 2 h followed by RNA extraction, TUC treatment and amplicon sequencing of *p21, CCNT1* and *ND1*. Labeling efficiency was calculated and corrected for random mutation using values derived from the DMSO control sample. Mean is shown, error bars represent SD (N=2, 3 replicates each).

Table S-1. Sequences of primers used for amplicon generation (TUC-seq; barcodes not shown) and RT-qPCR.

**TUC-seq primer**		
mRNA target	*Forward primer*	*Reverse primer*
p21	5′-CCCAAGCTCTACCTTCCC	5′-AAGGCAGAAGATGTAGAG
CCNT1	5′-CTGGCTTAAGTACCCAAAG	5′-GCATCCAGAGCTGAGGTG
ND1	5′-ACAACTACGCAAAGGCCC	5′-TGATGGCTAGGGTGACTT
**RT-qPCR primer**		
mRNA target	*Forward primer*	*Reverse primer*
GAPDH	5′-GGCTGAGAACGGGAAGCTTG	5′-AATGAGCCCCAGCCTTCTCC
HGPRT	5′-AGCCCTGGCGTCGTGATTAG	5′-AGCAAGACGTTCAGTCCTGTCC

7. References

[1] S. Moreno, M. Brunner, I. Delazer, D. Rieder, A. Lusser, R. Micura, Synthesis of 4-thiouridines with prodrug functionalization for RNA metabolic labeling. *RSC Chem. Biol*. **2022**, *3*, 447–455.

[2] C. Sheu, C.S. Foote, Deuterium spin probes of side-chain dynamics in proteins. 1. Measurement of five relaxation rates per deuteron in (13)C-labeled and fractionally (2)H-enriched proteins in solution. *J. Am. Chem. Soc.* **1995**, *117*, 6439–6442.

[3] A. Gupte, J.K. Buolamwini, Novel C2-purine position analogs of nitrobenzylmercaptopurine riboside as human equilibrative nucleoside transporter 1 inhibitors. *Bioorg. Med. Chem.* **2007**, *15*, 7726–7737.

[4] T. Kaur, A. Menon, A.L. Garner, Synthesis of 7-benzylguanosine cap-analogue conjugates for eIF4E targeted degradation. *Europ. J. Med. Chem.* **2019**, *166*, 339–350.

[5] T. Inde, S. Nishizawa, Y. Hattori, T. Kanamori, H. Yuasa, K. Seio, M. Sekine, A. Ohkubo, Synthesis of and triplex formation in oligonucleotides containing 2'-deoxy-6-thioxanthosine. *Bioorg. Med. Chem.* **2018**, *26*, 3785–3790.

[6] G. Macchione, S. Maza, M. Mar Kayser, J.L. de Paz, P.M. Nieto, Synthesis of Chondroitin Sulfate Oligosaccharides Using *N*-(Tetrachlorophthaloyl)- and *N*-(Trifluoroacetyl)galactosamine Building Blocks. *Eur. J. Org. Chem*. **2014**, *18*, 3868–3884.

[7] W. Shen, J.-S. Kim, P.E. Kish, J. Zhang, S. Mitchell, B.G. Gentry, J.M. Breitenbach, J.C. Drach, J. Hilfinger, Design and synthesis of vidarabine prodrugs as antiviral agents. *Bioorg. Med. Chem. Lett.* **2009**, *19*, 792–796.

[8] G. Painter, G. R. Blueming, A. de La Rosa, D. C. Liotta, Nucleotide and nucleoside therapeutic compositions and uses related thereto (2017), WO2017106710A1.

[9] C. Meier, A. Lomp, A. Meerbach, P. Wutzler, *Cyclo*Sal-BVDUMP pronucleotides: how to convert an antiviral-inactive nucleoside analogue into a bioactive compound against EBV. *J. Med. Chem.* **2002**, *45*, 5157–5172.

[10] W. Shen, J.-S. Kim, J. Hilfinger, Expedient total synthesis of triciribine and Its prodrugs *Synth. Commun.* **2012**, *42*, 358–374.
